# Transcriptomic and phenotypic convergence of neurodevelopmental disorder risk genes in vitro and in vivo

**DOI:** 10.1038/s41593-026-02247-7

**Published:** 2026-04-24

**Authors:** Meilin Fernandez Garcia, Kayla Retallick-Townsley, April Pruitt, Elizabeth A. Davidson, Novin Balafkan, Jonathan Warrell, Tzu-Chieh Huang, Alfred Kibowen, Zhiyuan Chu, Yi Dai, Sarah E. Fitzpatrick, Ran Meng, Annabel Sen, Sophie Cohen, Olivia Livoti, Suha Khan, Charlotte Becker, Andre Luiz Teles e Silva, Jenny Liu, Grace Dossou, Jen Cheung, Susanna Liu, Sadaf Ghorbani, P. J. Michael Deans, Marisa DeCiucis, Prashant Emani, Huanyao Gao, Hongying Shen, Mark Gerstein, Zuoheng Wang, Laura M. Huckins, Ellen J. Hoffman, Kristen Brennand

**Affiliations:** 1 Division of Molecular Psychiatry, Department of Psychiatry, Yale University School of Medicine, New Haven, CT USA; 2 Pamela Sklar Division of Psychiatric Genomics, Department of Genetics and Genomics, Icahn Institute of Genomics and Multiscale Biology, Nash Family Department of Neuroscience, Friedman Brain Institute, Icahn School of Medicine at Mount Sinai, New York, NY USA; 3 Interdepartmental Neuroscience Program, Yale School of Medicine, New Haven, CT USA; 4 Child Study Center, Yale University School of Medicine, New Haven, CT USA; 5 Mohn Research Centre for Regenerative Medicine, Haukeland University Hospital, Bergen, Norway; 6 Department of Molecular Biophysics & Biochemistry and Program in Computational Biology and Bioinformatics, Yale University, New Haven, CT USA; 7 BD2: Breakthrough Discoveries for Thriving with Bipolar Disorder, Easton, MD USA; 8 Department of Biostatistics, Yale University School of Public Health, New Haven, CT USA; 9 Medical Scientist Training Program, Yale School of Medicine, New Haven, CT USA; 10 Hospital Israelita Albert Einstein, São Paulo, Brazil; 11 Department of Cellular & Molecular Physiology, Yale School of Medicine, New Haven, CT USA; 12 Systems Biology Institute, Yale West Campus, West Haven, CT USA; 13 Department of Biomedical Informatics and Data Science, Yale School of Medicine, New Haven, CT USA; 14 Department of Neuroscience, Yale University School of Medicine, New Haven, CT USA; 15 Wu Tsai Institute, Yale University School of Medicine, New Haven, CT USA; 16 Department of Genetics, Yale University School of Medicine, New Haven, CT USA

**Keywords:** Functional genomics, Autism spectrum disorders, Molecular neuroscience

## Abstract

Diverse risk genes have been identified for neurodevelopmental disorders (NDDs), but how these genes converge on similar biological pathways in neurons, and thus give rise to similar phenotypes, is unclear. Here we apply a pooled CRISPR approach to successfully target 23 NDD loss-of-function genes with roles in chromatin biology and examine convergent effects on gene expression across human induced pluripotent stem cell-derived neural progenitor cells, glutamatergic neurons and GABAergic neurons. Points of convergence vary between these cell types, with the greatest number of convergent genes and strongest convergent networks in mature glutamatergic neurons, where they broadly represent synaptic, epigenetic and, unexpectedly, mitochondrial pathways. The most convergent networks were observed between NDD genes with shared biological annotations, clinical associations and co-expression patterns in human post-mortem brain. Drugs that were predicted to reverse convergent transcriptomic signatures and/or arousal and sensory processing behaviors ameliorated behavioral phenotypes in zebrafish NDD gene mutants. These results suggest that convergent effects of NDD risk genes could provide clinically useful insights.

## Abstract (web summary)

By studying 23 neurodevelopmental disorder genes across model systems and brain cell types, the authors uncovered shared downstream effects that converge on synaptic biology, epigenetic regulation and mitochondrial function.

## Main

Autism spectrum disorder (ASD) and related developmental delay (DD) are highly heritable^1^. The aggregate impact of common variants of small effect reflects most genetic risk^2^, but in as many as a quarter of cases, potentially damaging rare inherited and de novo mutations in risk genes are detected^3^. There is significant overlap between those genes affecting ASD^4^ and those more broadly affecting developmental^5^ and psychiatric^6,7^ disorders. Altogether, neurodevelopmental disorder (NDD) risk genes are typically expressed during cortical development^8^, particularly the excitatory and inhibitory lineages^4^, and broadly split between two functional classes: neuronal communication (for example, synaptic function) and gene expression regulation (for example, chromatin regulators and transcription factors). Over half of NDD genes have roles in gene expression regulation^4^, sharing substantial overlap in genomic binding sites in the brain^9^, and with targets enriched for NDD risk genes^10^. Yet, evidence to support the parsimonious explanation that regulatory NDD genes preferentially target synaptic NDD genes is lacking^4^. It remains unclear how disrupting NDD genes with distinct functions yields similar outcomes.

Many have proposed that diverse ASD genes have convergent downstream effects. NDD genes are co-expressed in the brain^11–13^, suggesting that they are regulated together and involved in related biological processes, and result in highly interconnected protein–protein interactomes^14,15^, indicating functional relationships between NDD proteins. Even as the number of NDD genes grows, risk genes continue to converge on a finite number of biological pathways, developmental stages, brain regions and cell types^16^. Disentangling these complex etiologies remains an outstanding challenge.

Given emerging evidence that epigenetic NDD genes have diverse and interconnected roles^17–19^, we tested the hypothesis that the nature of convergence is influenced by developmental and cell-type contexts. We report a pooled CRISPR-knockout (KO) strategy successfully targeting loss-of-function (LoF) mutations to 23 NDD genes, most with roles in chromatin biology, to examine effects on gene expression in induced neural progenitor cells (iNPCs), glutamatergic neurons and GABAergic neurons. We describe convergent networks that were distinct between cell types, strongest in neurons, where they were enriched in synaptic biology, epigenetic regulation and, unexpectedly, mitochondrial function. Machine learning tools allowed us to extend our analyses in silico across all known NDD genes, resolving how the degree of convergence between risk genes was influenced by clinical associations, biological function and co-expression patterns in the human post-mortem brain. Convergent analyses successfully predicted drugs capable of suppressing phenotypes in NDD gene zebrafish mutants, suggesting that analyses of convergent gene expression highlight behaviorally relevant pathways, and may in turn be useful in patient stratification or treatment development.

## Results

### A systematic comparison of NDD gene effects across neuronal cell types

**Fig. 1:**
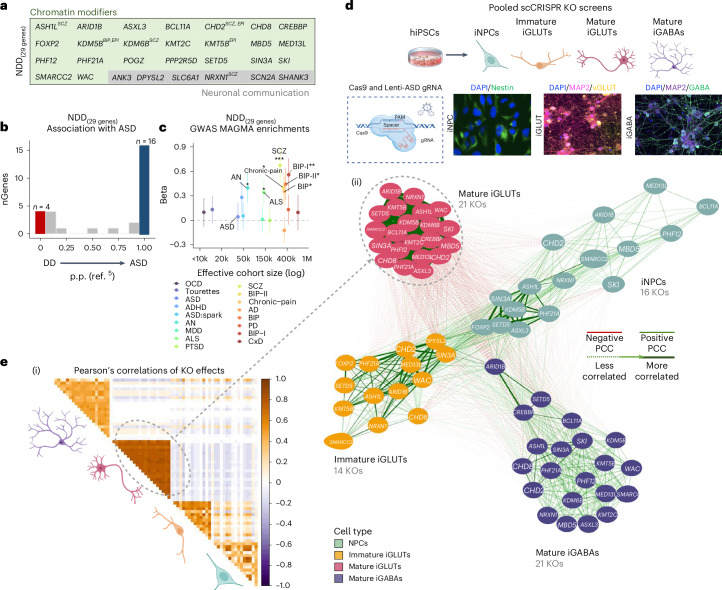
KO effects of 21 NDD risk genes are most strongly correlated in mature neurons. **a**, List of rare-variant target risk genes associated with NDDs separated by chromatin modifiers and neuronal communication genes. Bold gene names indicate strong associations with ASD based on ref. ^5^. Gene targets of rare variants associated with SCZ, epilepsy (EPI) and bipolar disorder (BIP) are annotated. **b**, Strength of association with ASD, as estimated by distribution of posterior probability (p.p.) scores from ref. ^5^; 4 of 29 NDD genes were more strongly associated with DD (blue; p.p. ≤ 0.1) while 16 of 29 were more strongly associated with ASD (red; p.p. ≥ 0.9). Further annotations of individual risk genes are shown in Supplementary Figs. 1 and 2. **c**, One-sided, positive MAGMA GSEA of targeted genes across GWAS for anorexia nervosa (AN), chronic pain, amyotrophic lateral sclerosis (ALS), SCZ, BIP, BIP-I (bipolar subtype 1) and BIP-II (bipolar subtype 2). FDR multiple testing correction was performed to adjust for multiple gene set comparisons: ^#^Nominal *P* < 0.05, *FDR < 0.05, **FDR < 0.01, ***FDR < 0.001. Error bars indicate the standard error of beta (the regression coefficient). **d**, Schematic of hiPSC-derived cell-type-specific scCRISPR-KO screen. Representative immunofluorescence for markers of NPCs (DAPI/Nestin), mature iGLUTs (DAPI/MAP2/vGLUT) and mature iGABAs (DAPI/MAP2/GABA). **e**, Transcriptomic impact of NDD gene KO represented as two-tailed Pearson’s correlation across nominally significant (*P* < 0.01) DEGs. (i) Pearson’s correlation matrix of log_2_FC DEGs across all NDDs and cell types. (ii) Cross-cell-type correlation network diagram (based on Pearson’s correlations) across NDD perturbations (number of NDD gene KO perturbations resolved indicated in parentheses); the mature iGLUT cluster was most dense, and the iNPC most sparse. Illustrations in **d** and **e** created in BioRender; Townsley, K. https://biorender.com/rvk1zn2 (2026). PCC, Pearson’s correlation coefficient. Source data

From 102 highly penetrant LoF gene mutations associated with NDD (previously described as 58 gene expression regulation, 24 neuronal communication and 20 other)^4^, we used gene ontology and primary literature to identify 21 epigenetic modifiers specifically involved in chromatin organization, rearrangement and modification (*ASH1L*, *ARID1B*, *ASXL3*, *BCL11A*, *CHD2*, *CHD8*, *CREBBP*, *PPP2R5D*, *KDM5B*, *KDM6B*, *KMT2C*, *KMT5B* (*SUV420H1*), *MBD5*, *MED13L*, *PHF12*, *PHF21A*, *SETD5*, *SIN3A*, *SKI*, *SMARCC2*, *WAC*), as well as two transcription factors with putative roles as chromatin regulators (*FOXP2*, *POGZ*). Three extensively studied synaptic genes (*NRXN1*, *SCN2A*, *SHANK3*) and three under-explored neuronal communication genes (*ANK3*, *DPYSL2*, *SLC6A1*) strongly associated with NDD were added (Supplementary Fig. 1a). Many of these 29 genes differed in relative frequency of LoF gene mutations between ASD (*n* = 16) and DD (*n* = 4)^5^, schizophrenia (SCZ)^20^ and epilepsy^21,22^ (Fig. 1a,b and Supplementary Fig. 1b), as well as general associations with genome-wide association studies (GWASs) for many neuropsychiatric disorders (MAGMA^23^) (Fig. 1c and Supplementary Fig. 1c), indicating a pleotropic effect consistent with the shared genetic liability across neuropsychiatric disorders^24^. iNPCs, iGLUTs and iGABAs (Supplementary Fig. 2a), as well as their in vivo fetal counterparts (Supplementary Fig. 2b), expressed all genes prioritized herein^25^.

Towards resolving whether regulatory genes confer continuous or distinct periods of susceptibility across neurodevelopment, we knocked out regulatory NDD genes in neural progenitor cells (NPCs) (SNaPs^26^, here termed iNPCs), immature and mature glutamatergic neurons (iGLUTs)^27^ and mature GABAergic neurons (iGABAs)^28^ (Fig. 1d). A pooled CRISPR approach (expanded CRISPR-compatible cellular indexing of transcriptomes and epitopes by sequencing (ECCITE-seq)^29^) combined direct detection of single guide RNAs and single-cell RNA sequencing (scRNA-seq) to compare LoF effects across 29 NDD genes. The CRISPR-KO library was generated from pre-validated guide RNAs (gRNAs) (three to four gRNAs per gene; Supplementary Table 1). Sequencing of the gRNA library confirmed the presence of gRNAs targeting 24 genes (*ANK3*, *ARID1B*, *ASH1L*, *ASXL3*, *BCL11A*, *CHD2*, *CHD8*, *DPYSL2*, *FOXP2*, *KMT5B* (*SUV420H1*), *KDM5B*, *KDM6B*, *KMT2C*, *MBD5*, *MED13L*, *NRXN1*, *PHF12*, *PHF21A*, *SCN2A*, *SETD5*, *SIN3A*, *SKI*, *SMARCC2*, *WAC*), but three (*DPYSL2*, *FOXP2*, *SCN2A*) were present at lower frequency (Supplementary Fig. 3b,c).

Control human induced pluripotent stem cells (hiPSCs) were induced to iNPCs, iGLUTs and iGABAs (Supplementary Fig. 3a), transduced first with lentiviral-Cas9v2 (Addgene, cat. no. 98291) and subsequently with the pooled lentiviral gRNA library 3 d before collection, at day 7 (iNPC and immature iGLUT), day 21 (iGLUT) and day 36 (iGABA) (experimental workflow, Supplementary Fig. 4a; computational workflow, Supplementary Fig. 4b; experimental validation of CRISPR editing efficiency, Supplementary Fig. 5). After filtering and quality control (Supplementary Fig. 4c–e), we resolved NDD transcriptomes for 118,436 single cells: 25,402 iNPC, 38,097 immature (day 7) iGLUT, 28,388 mature (day 21) iGLUT and 26,549 mature (day 36) iGABA. Because the original gene-expression-based clustering was driven by cellular heterogeneity, cell quality and sequencing lane effects (Supplementary Fig. 6a), independent of gRNA identity, we removed cells with high expression of subtype markers and adjusted for cellular heterogeneity (Supplementary Fig. 6b,c and Supplementary Tables 2 and 3). ‘Weighted-nearest neighbor’ (WNN) analysis assigned clusters based on both gRNA identity class and gene expression to ensure that cells assigned to a gRNA identity class demonstrated successful perturbation of the targeted NDD gene^30^. For those WNN clusters where most cells were assigned to a single KO target, the transcriptomic signatures were compared with nontargeting scramble control clusters. Altogether, 35,777 cells were used for downstream analyses: 12,107 iNPC, 3,171 immature iGLUT, 11,802 mature iGLUT and 8,697 mature iGABA. An average of 474 cells were assigned to each individual single guide RNA (757 iNPC, 227 immature iGLUT, 562 mature iGLUT and 414 mature iGABA), totaling 33,150 perturbed cells and 2,627 controls (882 iNPC, 90 immature iGLUT, 1,258 mature iGLUT and 397 mature iGABA). The gene expression patterns of nonperturbed iNPCs and iNeurons (>30% of all pooled cells) were significantly correlated with fetal brain cells and cortical adult neurons.

Successful perturbations (scCRISPR-KO) were identified for 23 NDD genes (Supplementary Figs. 6 and 7): 16 in iNPCs, 14 in immature iGLUT neurons and 21 in mature iGLUT and iGABA neurons (Supplementary Fig. 6). Nine NDD genes were perturbed in all four cell types (*ARID1B*, *ASH1L*, *CHD2*, *MED13L*, *NRXN1*, *PHF21A*, *SETD5*, *SIN3A*, *SMARCC2*; Supplementary Fig. 7a,b). For most NDD genes, KO in mature iGLUTs yielded the largest number of differentially expressed genes (DEGs, *P*_FDR_ < 0.05 (FDR, false discovery rate)) (Supplementary Fig. 7b), an effect that was not driven by differences in the extent of perturbation of the NDD gene itself between cell types (Supplementary Fig. 7c(i)). The transcriptomic effects of individual NDD genes cluster by cell type: the strongest NDD gene correlations are in mature iGLUTs; that is, all nominally significant (*P* < 0.01) log_2_fold change (FC) DEGs are most highly correlated with each other and least correlated with the other cell types, whether relative to all scramble control cells (Fig. 1e(i),(ii) and Supplementary Fig. 7c(ii)) or to random subsets of scramble control cells (Supplementary Fig. 8a,b). DEGs across individual NDDs shared significant gene ontology enrichments (Supplementary Fig. 8c), with mature iGLUTs frequently enriched for SCZ GWAS genes (12 of 21 NDD genes), and mature iGABAs for migraine GWAS genes (8 of 21) (Supplementary Fig. 9).

Unsurprisingly, given the greater within-cell-type correlations between NDD genes and the unique pathway enrichments across cell types, very few DEGs shared significance and direction of effect for the same NDD gene perturbation across all four cell types (FDR-adjusted *P*_meta_ < 0.05, Cochran’s heterogeneity *Q*-test *P*_Het_ > 0.05; computational workflow, Supplementary Fig. 10a); in fact, the only common DEG between cell types was frequently the targeted NDD gene itself. With a more relaxed statistical threshold (nominal *P* < 0.05), modest shared effects of individual NDD genes could be resolved across cell types. These effects rarely resulted in perturbation of the other NDD genes themselves (Supplementary Fig. 10b), showed very little overlap between NDD genes (Supplementary Fig. 10c) and showed no significant enrichments with psychiatric GWAS after multiple testing correction (Supplementary Fig. 10d).

### NDD gene KOs resulted in cell-type-specific convergent genes and networks that were strongest in glutamatergic neurons

**Fig. 2:**
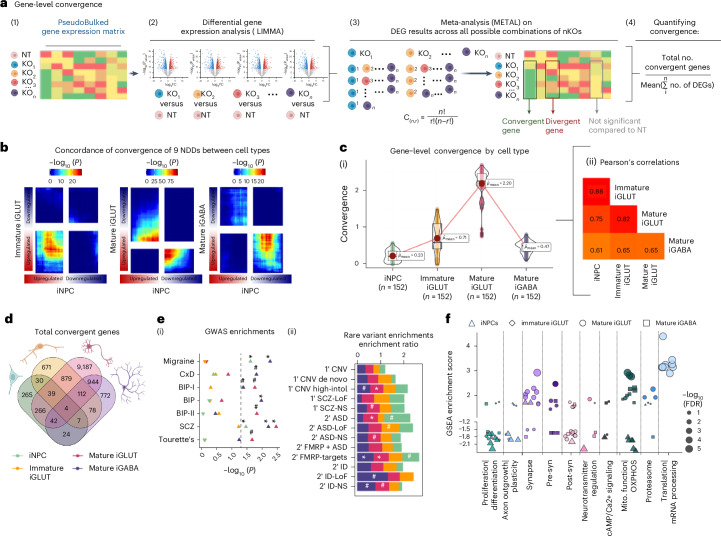
Gene-level convergence is greatest in mature glutamatergic neurons. In total, nine NDD genes showed evidence of KO across all four cell types: *ARID1B*, *ASH1L*, *CHD2*, *MED13L*, *NRXN1*, *PHF21A*, *SETD5*, *SIN3A*, *SMARCC2*. For these nine, ‘convergent genes’ are defined as those DEGs with significant and shared direction of effect across all NDD gene perturbations. **a**, Schematic explaining cell-type-specific convergence at the individual gene level via differential gene expression meta-analysis (FDR-adjusted *P*_meta_ < 0.05, Cochran’s heterogeneity *Q*-test *P*_Het_ > 0.05). **b**, Convergence across nine NDD genes is unique to each cell type, using rank–rank hypergeometric (RRHO) test to explore correlation of convergent genes shared across nine NDD perturbations (RRHO score = −log_10_ (*P* value) × sign(fold change)) between cell types. The top-right quadrant represents downregulated genes (meta-analysis *Z*-score > 0) for the *y*-axis and *x*-axis cell types. The bottom-left quadrant represents upregulated convergent genes (meta-analysis *Z*-score < 0) for the *y*-axis and *x*-axis cell types. Significance is represented by color, with red regions representing significantly convergent gene expression. **c**, (i) The average strength of convergence, measured as the ratio of convergent genes to the average number of DEGs across all 152 unique combinations of 2–5 genes from the nine NDD genes, was highest in iGLUTs. (ii) The magnitude of convergence between the same NDDs tested in different cell types was highly positively correlated (two-tailed Pearson’s correlation, Holm’s multiple testing correction was performed, *P*_holm_ < 2.2 × 10^−16^), with the strongest relationship between immature and mature iGLUTs. In the box plots, the median is represent by the line (center) and the mean as the red point. The lower and upper hinges correspond to the first and third quartiles (the 25th and 75th percentiles). The upper and lower whiskers extend up to 1.5 × interquartile range (IQR). All data points are plotted individually. **d**, Venn diagram representing the absolute overlap of cell-type-specific convergent genes shared across nine NDDs (regardless of whether convergent genes were perturbed in the same direction between cell types). **e**, (i) One-sided, positive MAGMA gene set enrichment −log_10_(*P* value) of cell-type**-**specific (color of points) convergence and GWAS-risk-associated genes with significance after multiple testing correction indicated as follows: ^#^unadjusted *P* ≤ 0.05, *FDR ≤ 0.05, **FDR < 0.01, ***FDR < 0.001. The direction of the triangles indicates a positive (upwards triangle) or negative (downwards triangle) enrichment beta. (ii) Over-representation analysis (ORA) enrichment ratios of cell-type-specific (color of bars) convergence and rare-variant target genes. Significance after multiple testing correction indicated as follows: ^#^unadjusted *P* ≤ 0.05, *FDR ≤ 0.05, **FDR < 0.01, ***FDR < 0.001. **f**, Two-sided GSEA identified downstream pathways involved in neural proliferation, neurite outgrowth, synaptic vesicle transport and mitochondrial function as cell-type-specific targets of convergent genes across nine NDDs. FDR multiple testing correction was performed. Results in the figure panel were filtered for pathways with nominal *P* < 0.05. Normalized GSEA enrichment scores represent the direction of enrichment based on the meta-analyzed *Z*-score for each convergent gene. Cell type is represented by shape and the size of each point represents the −log_10_(FDR). Illustrations in **a** created in BioRender; Townsley, K. https://biorender.com/efkzzf6 (2026). nKOs, number of KO genes tested for convergence; NT, XXX. Source data

‘Convergent genes’ (Fig. 2) are those DEGs with significant and shared direction of effect across all NDD gene perturbations (FDR-adjusted *P*_meta_ < 0.05, Cochran’s heterogeneity *Q*-test *P*_Het_ > 0.05)^31^ (computational workflow, Fig. 2a). Across the nine NDD genes perturbed in all four cell types (*ARID1B*, *ASH1L*, *CHD2*, *MED13L*, *NRXN1*, *PHF21A*, *SETD5*, *SIN3A*, *SMARCC2*), convergence was highly cell-type-specific (Fig. 2, Supplementary Fig. 11a–c and Supplementary Data 2). Although the strength of convergence correlated across cell types (Fig. 2c(ii)), it was greatest in mature iGLUTs (quantified as the ratio of convergent genes to the average number of DEGs across all 152 unique 2–5-gene combinations of these nine NDD genes) (Fig. 2c(i)). While the ‘top’ convergent gene was unique for each cell type (Supplementary Table 4), >50% of convergent genes in NPCs (52%), immature iGLUTs (57%) and mature iGABAs (56%) overlapped with convergent genes in mature iGLUTs; of note, shared convergent genes were not necessarily perturbed in the same direction between cell types (Fig. 2d). Mature iGLUTs had the greatest total number of convergent genes (11,473); however, immature iGLUTs had the highest ratio of convergent genes after correction for the number of DEGs across perturbations.

Convergent genes were enriched for SCZ GWAS loci (MAGMA^23^, FDR < 0.05) (Fig. 2e(i)), rare ASD and Fragile X Mental Retardation 1 protein (FMRP) target genes (FDR < 0.05) (Fig. 2e(ii)), and pathways involved in neurodevelopment, mitochondrial function and translational regulation (Supplementary Fig. 12). When tested again across the 21 NDD genes perturbed in both iGLUTs and iGABAs, mature iGLUTs again showed the largest absolute number of convergent genes (iGLUTs, 10,557, Supplementary Fig. 13a; iGABAs, 892, Supplementary Fig. 13b). Intriguingly, although convergent genes were highly cell-type-specific, those NDD gene combinations that were highly convergent in one cell type were likely to be convergent in others; in neurons, top convergent sets most frequently included *ARID1B*, *SETD5* and *NRXN1* (Supplementary Fig. 11d).

**Fig. 3:**
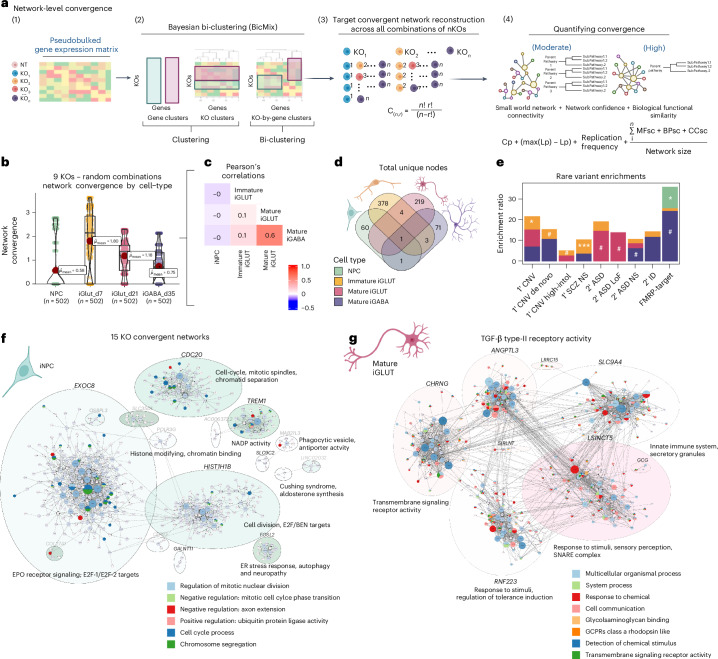
Network-level convergence resolves cell-type-specific and developmental-specific node genes. ‘Convergent networks’ are co-expressed genes that share similar expression patterns across NDD gene perturbations, here resolved for the nine NDD KOs resolved across all four cell types: *ARID1B*, *ASH1L*, *CHD2*, *MED13L*, *NRXN1*, *PHF21A*, *SETD5*, *SIN3A*, *SMARCC2*. **a**, Schematic explaining cell-type-specific convergence at the network level using Bayesian bi-clustering and unsupervised network reconstruction. **b**, Strength of network convergence across all random combinations of nine NDD KO perturbations by cell type. (i) The mean strength of network convergence is significantly different by cell type, with the highest convergence present in immature iGLUTs. The same KO combinations tested in one cell type may not resolve convergence in another cell type. Each point represents a resolved network, and its calculated convergence strength. Dots that represent the same combinations of KO perturbations, but tested in each cell type, are connected by a line. In the box plots, the median is represented by the line (center) and the mean as the red point. The lower and upper hinges correspond to the first and third quartiles (the 25th and 75th percentiles). The upper and lower whiskers extend from the hinge to the largest or smallest value up to 1.5 × IQR. All data points are plotted individually. **c**, Convergent network strength was most correlated between mature iGLUTs and iGABAs (two-tailed Pearson’s correlation test with Holm’s multiple testing correction; PCC = 0.6, *P*_Holm_ < 2.2 × 10^−16^). Convergent network strength in iNPCs was not correlated with network strength in neurons. **d**, Venn diagrams of the total number of unique node genes within convergent networks for each cell type. The lack of overlapping node genes between cell types (**d**), as well as the weak correlations of convergence strength between immature and mature cell types (**c**), suggest greater cell-type specificity in the magnitude of network-level convergence compared with gene-level convergence. **e**, One-sided enrichment ratios from ORA of cell-type-specific (color of bars) convergent node genes for rare-variant targets. FDR-based multiple testing correction was performed: ^#^unadjusted *P* ≤ 0.05, *FDR ≤ 0.05, **FDR < 0.01, ***FDR < 0.001. **f**,**g**, Representative cell-type-specific network plots for convergence across 15 genes (*ARID1B*, *ASH1L*, *ASXL3*, *BCL11A*, *KDM5B*, *CHD2*, *MBD5*, *MED13L*, *NRXN1*, *PHF12*, *PHF21A*, *SETD5*, *SIN3A*, *SKI*, *SMARRC2*) from iNPCs (**f**) and mature iGLUTs (**g**). Network genes were filtered for protein-coding genes, clustered and annotated based on the primary node gene for each cluster. GSEA of the networks identified unique functions by cell type. Convergent networks in iNPCs were enriched for pathways associated with neurogenesis (for example, cell cycle, cell division, EPO signaling), and in mature iGLUTs for pathways associated with synaptic function (transmembrane transport and receptor signaling, secretory vesicles, SNARE complex). Illustrations in **a** created in BioRender; Townsley, K. https://biorender.com/efkzzf6 (2026). Source data

Given that the biological impact of convergence is likely to be impacted by the strength of shared gene regulatory relationships and functions, we re-examined convergence within the framework of co-expression networks (Bayesian bi-clustering). ‘Convergent networks’ (Fig. 3) are co-expressed genes that share similar expression patterns across NDD gene perturbations^31^ (computational workflow, Fig. 3a). The network connectivity score (‘network convergence’) informs the strength and composition across cell types (that is, networks with more interconnectedness and containing genes with greater functional similarity have increased convergence). Convergent networks generated from the nine NDD genes perturbed in all four cell types (Fig. 3b) or across the 21 NDDs genes in both iGLUTs and iGABAs (Supplementary Fig. 13c) revealed the greatest convergent network strength in iGLUTs. Network-level convergence was weakly correlated between cell types (Fig. 3c); the number of convergent unique network nodes was greatest in iGLUTs, distinct across cell types (Fig. 3d, Supplementary Tables 5 and 6 and Supplementary Data 2) and significantly enriched for rare variants linked to SCZ and ASD (Fig. 3e and Supplementary Tables 5 and 6). Convergent networks in iNPCs highlighted pathways associated with neurogenesis (for example, cell cycle, cell division, EPO signaling) (Fig. 3f), while in mature iGLUTs they were enriched for synaptic function (transmembrane transport and receptor signaling, secretory vesicles, SNARE complex) (Fig. 3g).

### Convergent networks are strongest between NDD genes with shared co-expression patterns in the post-mortem brain, biological annotations (synaptic or epigenetic) and clinical outcomes (ASD or DD)

**Fig. 4:**
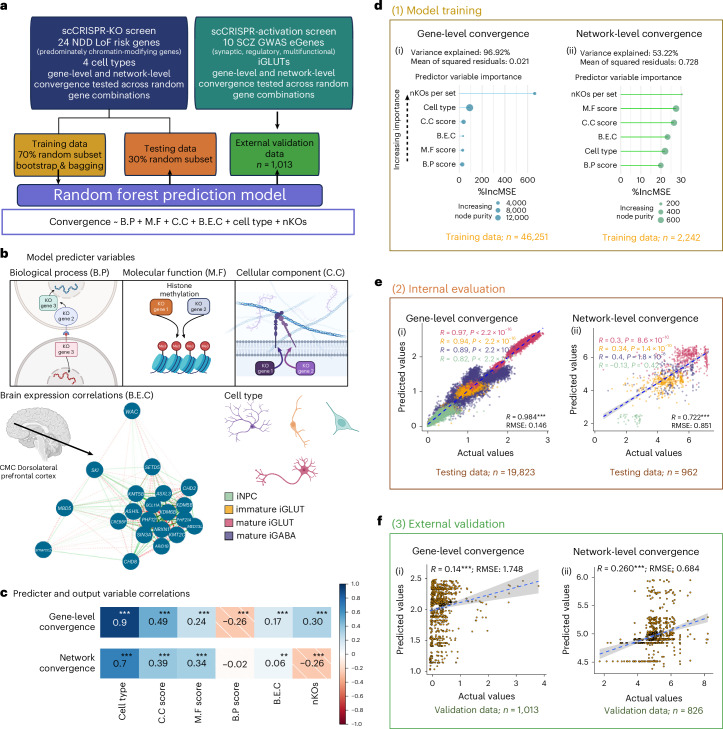
Functional similarity and brain co-expression between NDD genes predict gene-level and network-level convergence, with unique influences by cell type. **a**, Schematic for training random forest models for gene- and network-level convergence with external validation in an SCZ CRISPRa screen. **b**, Predictor variables included in the model include scores of functional similarity, DLPFC brain co-expression, cell type and the number of KOs (B.P score, semantic similarity of GO: Biological Process membership between KO genes; C.C score, semantic similarity of GO: Cellular Component membership between KO genes; M.F score, semantic similarity of GO: Molecular Functions membership between KO genes; B.E.C, DLPFC expression correlations between KO genes). **c**, Two-tailed Pearson’s correlations of predictor variables and gene-level and network-level convergence (**P_Bonferroni_ ≤ 0.01, ***P_Bonferroni_ ≤ 0.01). **d**, Functional similarity, brain co-expression, cell type and the number of KOs assayed strongly predicted gene-level convergence (97% variance explained by the model; mean of squared residuals = 0.02) and moderately predicted network-level convergence (53% variance explained; mean of squared residuals = 0.73). (i),(ii) Importance of each of the predictor variables was assessed by two metrics: the percentage mean increase in squared residuals (%IncMSE) and the increase in node purity. In the model, number of KO genes in a set is the most important predictor of convergence based on %IncMSE, but not node purity. However, the impact of nKOs on gene-level convergence is much stronger, likely an artifact of the method used for measuring convergence. For network-level convergence, each variable has an IncMSE of 20–30%. **e**, Internal evaluation of the model using 30% of the original data resulted in high concordance between convergence predicted by the model and the measured convergence. Predicted gene-level (i) (gene-level convergence: *n* = 19,823; two-tailed Pearson’s *R* = 0.984; *P*_Holm_ < 1 × 10^−150^; root mean squared error (RMSE) = 0.15) and network-level (ii) convergence (network-level convergence: *n* = 962; *ρ* = 0.722; *P*_Holm_ = 2.4 × 10^−149^; RMSE = 0.85) by the model strongly correlated with the measured convergence in the testing sets. Correlations of predicted versus accrual convergence values are color-coded by cell type with corresponding color-coded correlations and *P* values listed in the upper-right corners of the scatterplots. Lines represent the smooth conditional mean with 95% confidence bands. **f**, External validation of the random forest predication in an independent scCRISPRa screen of SCZ target genes (10 perturbations) predicted showed moderate, but significant, correlation between convergence by the model and the measured convergence. (i) Gene-level convergence (*n* = 1,013, Pearson’s *R* = 0.14, *P* = 1.1 × 10^−5^, RMSE = 1.748). (ii) Network-level convergence (*n* = 826, Pearson’s *R* = 0.26, *P* = 2.9 × 10^−14^, RMSE = 0.68). Lines represent the smooth conditional mean with 95% confidence bands. Illustrations in **b** created in BioRender; Townsley, K. https://biorender.com/5xlpxkm and https://biorender.com/rvk1zn2 (2026). Source data

To resolve the extent to which functional similarity and co-expression patterns between NDD genes predicted convergence, we trained a prediction model (random forest linear regression)^32^ using 70% of our data, evaluated it using 30% of our data and validated in an external dataset^31^ (computational workflow, Fig. 4a; model predictor variables, Fig. 6b; more information, Supplementary Figs. 14 and 15). Cell type, brain co-expression (dorsolateral prefrontal cortex (DLPFC)) and functional similarity (that is, gene ontology) correlate with convergence (Fig. 4c) and well-predicted gene-level convergence (97% variance explained; root mean squared error (RMSE) = 0.021) (Fig. 4d(i)) and moderately predicted network-level convergence (53% variance explained; RMSE = 0.73) (Fig. 4d(ii)). Our trained model accurately predicted gene-level (Pearson’s *R* = 0.998, *P* < 0.001, RMSE = 0.15) (Fig. 4e(i) and Supplementary Fig. 15c) and network-level convergence in our testing set (*R* = 0.72, *P* < 2.2 × 10^−16^, RMSE = 0.85) (Fig. 4e(ii) and Supplementary Fig. 15d), and performed moderately well in predicting network-level convergence (*R* = 0.26, *P* < 0.001, RMSE = 0.68) (Fig. 4f(ii) and Supplementary Fig. 15e(ii)) and to a lesser extent gene-level convergence (*R* = 0.14, *P* < 0.001, RMSE = 1.75) (Fig. 4f(i) and Supplementary Fig. 15e(i)) in the external dataset.

To query whether convergence reflected clinical associations to ASD or DD, we again quantified convergence as the ratio of convergent genes to the average number of DEGs (Fig. 2e), here across all (2–5-gene) combinations of all NDD genes perturbed in each cell type (for example, 27,824 unique combinations of 21 NDD genes in iGLUTs and iGABAs; Supplementary Fig. 16a). Convergence, both gene-level (Supplementary Fig. 16a,c) and network-level (Supplementary Fig. 16b,d), was greater between genes with stronger associations to ASD compared with DD^5^, particularly in mature neurons (Supplementary Fig. 16e,f). Yet, this analysis was limited by the relatively small numbers of predominantly ASD (*n* = 16) and DD (*n* = 4) included in our dataset (Fig. 1b).

**Fig. 5:**
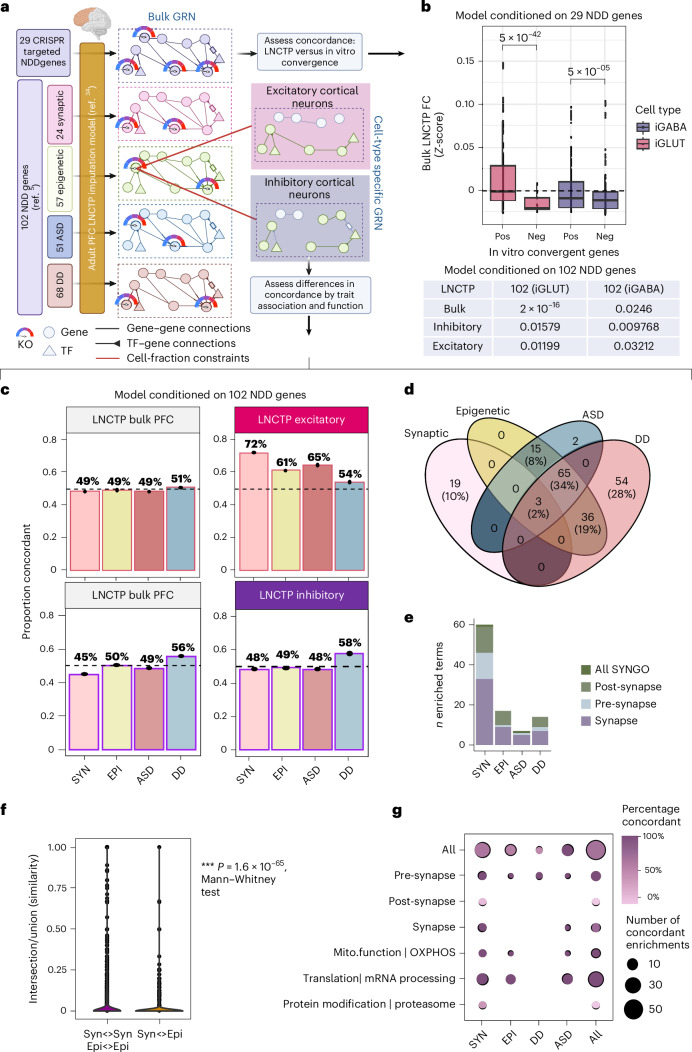
LNCTP predicts effects of convergent genes in silico*.* **a**, LNCTP imputation and perturbation model: an energy-based network model is trained to impute bulk and cell-type-specific expression data in the PFC over a population of post-mortem individuals from PsychENCODE using a panel of 1,325 genes and embedded cell-type-specific GRNs (LNCTP in silico model); a chosen gene is then perturbed by fixing its expression, and the effects on other genes are predicted by the model; in silico category-specific convergent genes are then identified by analyzing the FCs across subjects (LNCTP simulating perturbations). **b**, A perturbation-conditioned version of the LNCTP model applied negative perturbation to mimic the effect of the 29 CRISPR-KOs and predict downstream gene expression changes in the adult PFC. Predicted in silico log FCs for the in vitro positive and negative convergent genes across the 29 CRISPR perturbations, in bulk, excitatory and inhibitory neuron networks evaluated if in vitro convergence was replicated in adult PFC prediction models (LNCTP simulating perturbations, two-tailed *t*-test *P* values shown). In the box plots, center represents median, hinges represent 1st and 3rd quartiles. The upper and lower whiskers extend up to 1.5 × IQR. All data points are plotted individually. **c**, A perturbation-conditioned version of the LNCTP model applied a negative perturbation to mimic 102 NDD KOs and evaluate whether convergence measured across 29 NDDs in vitro remained convergent in a larger set. Proportion of genes showing same direction FCs in in silico and in vitro perturbations across classes of perturbation and cell type (left), and the intersection of convergent in silico genes across classes of perturbation (LNCTP in silico convergent genes, synaptic–epigenetic genes reduced and ASD–DD genes enriched, *P* < 1 × 10^−3^, two-tailed hypergeometric test). **d**, Venn diagram of in silico convergent genes across all categories by clinical (ASD versus DD) or functional (synaptic versus epigenetic) annotation. **e**, Number of terms enriched for convergent genes across all categories for 102 in silico perturbations. **f**, Semantic distance of pairs of enriched terms within or between sets determined by synaptic and epigenetic convergent gene rankings (LNCTP semantic distance test, two-tailed Mann–Whitney test). **g**, Percentage of concordant genes in each perturbation and ontology category within the leading-edge enriched genes (LNCTP in silico convergent genes). Illustrations in **a** created in BioRender; Townsley, K. https://biorender.com/6jv3bge (2026). TF, transcription factor. Source data

To extend our comparisons of convergence across larger sets of NDD genes, particularly those clinically defined as predominantly ASD or DD genes^5^, or those with biologically annotated synaptic or epigenetic roles^4^, we asked whether it was possible to train a machine learning model to predict cell-type-specific impacts of CRISPR-KO of all 102 NDD genes^4^. An integrative linear network of cell-type phenotypes (LNCTP) model, previously trained on >2.8 million nuclei from the prefrontal cortex (PFC) across 388 individuals, accurately imputed single-cell expression following simulated perturbations^33^. By conditioning the LNCTP model on reduced expression of the 29 NDD genes (Fig. 5a), we resolved downstream gene expression changes within three in silico post-mortem brain network models (bulk PFC tissue, excitatory neurons only and inhibitory neurons only), and compared these changes with convergent genes identified by in vitro scCRISPR-KO; the LNCTP model better replicated experimental iGLUT data (Fig. 5b).

Expanding in silico LNCTP model predictions of downstream gene expression changes to all 102 NDD genes (Fig. 5c–g and Supplementary Fig. 17) demonstrated greatest concordance with in vitro convergence in excitatory neurons, consistent with our findings that convergence was greater in iGLUTs (Figs. 2c and 3d). This concordance was more pronounced for synaptic NDD genes (*n* = 24) relative to regulatory genes (*n* = 58) (Fig. 5c). Predominantly ASD genes (*n* = 50) had greater concordance with convergence in excitatory neurons (Fig. 5c), whereas predominantly DD genes (*n* = 40) were moreso in inhibitory neurons (Fig. 5c). Overall, across functional or clinical categories, despite limited overlap in specific convergent genes (Fig. 5d) and terms (Fig. 5e,f), there was overall enrichment for synaptic, epigenetic and mitochondrial biology (Fig. 5g), consistent with in vitro scCRISPR-KO (Fig. 2f).

### Convergent genes and networks in glutamatergic neurons targeted synaptic, epigenetic and mitochondrial biology

**Fig. 6:**
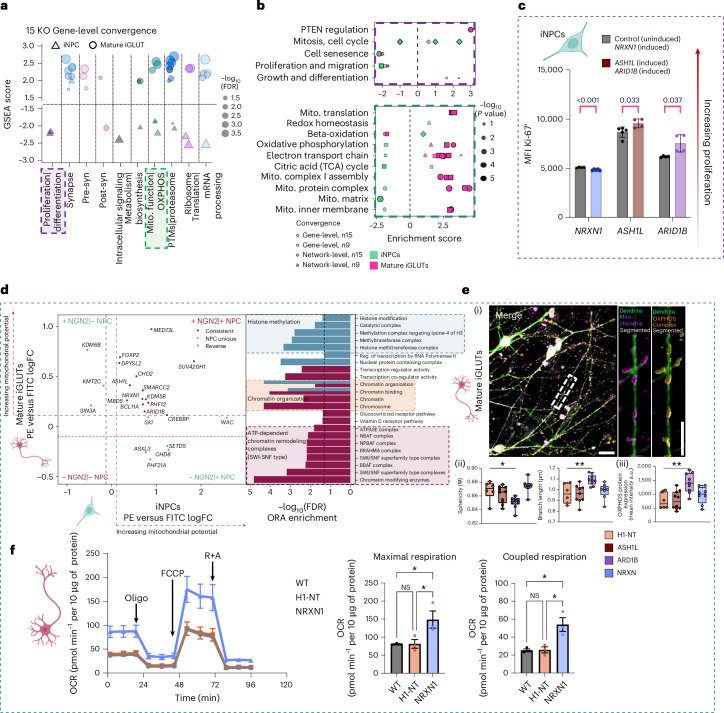
NDD KOs converge on mitochondrial function. **a**, Two-sided GSEA identified downstream pathways involved in neurogenesis, neurite outgrowth, synaptic biology and mitochondrial function as cell-type-specific targets of convergent genes across 15 NDD KOs (*ARID1B*, *ASH1L*, *ASXL3*, *BCL11A*, *KDM5B*, *CHD2*, *MBD5*, *MED13L*, *NRXN1*, *PHF12*, *PHF21A*, *SETD5*, *SIN3A*, *SKI*, *SMARRC2*) in iNPCs and mature iGLUTs. FDR-based multiple testing correction was performed and results were filtered for pathways with nominal *P* < 0.05 for plotting. Normalized GSEA scores represent the direction of enrichment based on the meta-analyzed *Z*-score for each convergent gene. Cell type is represented by shape and the size of each point represents the −log_10_(FDR). **b**, Summary of network- and gene-level pathway enrichments (two-sided GSEA, FDR-corrected *P* values for enrichment tests are reported in Supplementary Data 1) (from Figs. 2 and 3) for shared effects of nine and 15 NDD KOs in iNPCs and mature iGLUTs. **c**, Proliferation assessment of NPCs using Ki-67 median fluorescence intensity (MFI) measured with flow cytometry, WT (purple, no iCas9 induction) versus KO (green, doxycycline to induce iCas9). Ki-67 MFI was compared between uninduced and induced conditions for each gene using two-sided unpaired *t*-tests with Welch’s correction, followed by Benjamini–Hochberg FDR correction (*Q* = 5%). Bars show mean ± s.e.m. with individual biological replicates overlaid (*NRXN1*: *n* = 4 uninduced, *n* = 5 induced; *ASH1L*: *n* = 5 uninduced, *n* = 4 induced; *ARID1B*: *n* = 4 induced). LoF of target genes significantly decreased Ki-67 for *NRXN1* (mean difference −229.8; *P* = 0.0004; *Q* = 0.0008) and increased Ki-67 for *ASH1L* (difference 887.0; *P* = 0.0319; *Q* = 0.0335) and *ARID1B* (difference 1,350; *P* = 0.0535; *Q* = 0.0374). **d**, Scatter plot of gRNA log_2_FC (high- (PE-high) and low- (FITC-high) mitochondrial inner membrane potential (Δψm)-sensitive dye JC-1 membrane potential fractions) in NPCs (*x* axis) and mature iGLUT neurons (*y* axis), with points colored by enrichment category (shared NPC and iGLUT in red; distinct between NPC and iGLUT in blue). Right, bar chart of −log_10_(FDR) for over-represented gene sets in the gene KOs enriched in both lineages. **e**, (i) High resolution, high-throughput microscopy of mitochondrial morphology (scale bar: 10 μm): an isolated dendrite labeled with a dendritic marker (MAP2), mitochondrial marker (TOMM20) and marker of the OXPHOS complex (Total OXPHOS) (scale bar: 5 μm). (ii) Effect of ASH1L-KO (*n* = 10, 9), ARID1B-KO (*n* = 9) and NRXN1-KO (*n* = 8) on mitochondrial sphericity (H1-NT versus ARID1B, adjusted *P* = 0.0213) and branch length (H1-NT versus ARID1B, adjusted *P* = 0.0081) independent of changes in mitochondrial volume and surface area (Supplementary Figs. 20 and 21) compared with H1-NT (nontargeting control, *n* = 6). (iii) Effect of ASH1L-KO, ARID1B-KO and NRXN1-KO on average fluorescence intensity (H1-NT versus ARID1B, adjusted *P* = 0.0024) of OXPHOS proteins compared with H1-NT. Each data point indicates one well of a 96-well plate, representing hundreds of μm^2^ of neuronal area and tens of thousands of individual mitochondria (statistical analysis comparing KO with control was performed using a one-way ANOVA comparing H1-NT versus other groups (ASH1L, ARIDIB, NRXN1) with Šidák’s multiple comparisons, *adjusted *P* < 0.05, ** adjusted *P* < 0.01). **f**, Effect of NRXN1-KO on maximal respiration (WT versus H-NT: *P* = 0.9995; WT versus NRXN1: *P* = 0.0466; H-NT versus NRXN1: *P* = 0.0483) and coupled respiration (WT versus H-NT: *P* = 0.9973; WT versus NRXN: *P* = 0.0140; H-NT versus NRXN1: *P* = 0.0152) in iGLUTs. Data are presented as mean ± s.e.m. Statistical analysis was performed using one-way ANOVA. Each data point in the temporal plot represents the mean and s.e.m. across a 24-well Seahorse assay plate. The experiment was independently replicated twice, with the bar graph showing three wells from one representative replicate. The center of the box plots represents the median, the bounds the 25th and 75th percentiles, and the whiskers the minimum and maximum values of that group. NS, not significant; OCR, oxygen consumption rate; oligo, oligomycin; R+A, rotenone and antimycin A. Source data

Convergent genes and networks revealed cell-type-specific disease (Fig. 2e) and functional enrichments (Figs. 2f, 5g and 6a,b), many consistent with established NDD etiology in neurogenesis^18,34–41^ and synaptic biology^14,15,42,43^. For example, iNPCs were significantly enriched for pathways involved in proliferation and differentiation, whereas mature iGLUTs showed unique enrichments in neuronal communication (for example, pre-synaptic function) and regulation of gene expression (for example, messenger RNA processing and protein translation). Unexpectedly, both mature iGLUT and iGABA neurons were enriched for mitochondrial biology (for example, oxidative phosphorylation: mature iGLUTs: NES = 2.8, *P* < 2.2 × 10^−16^, FDR < 0.001; mature iGABAs: NES = 1.67, *P* = 0.023, FDR < 0.05).

To functionally validate the impact of LoF mutations on convergent pathways, we selected five high-confidence NDD genes (*KMT5B*, *NRXN1*, *CHD8*, *ASH1L*, *ARID1B*) representing distinct functional clusters (for example, chromatin remodelers, synaptic genes) for initial testing. We utilized a well-established doxycycline-inducible Cas9 system (iCas9)^44^ that allows for a robust comparison of gRNA-transduced iCas9 ‘induced’ (KO) cells with matched gRNA- and iCas9-transduced cells lacking expression of iCas9 (no doxycycline) to serve as an ‘uninduced’ baseline control. Five of five NDD genes (*KMT5B*, *NRXN1*, *CHD8*, *ASH1L*, *ARID1B*) tested in iCas9 NPCs (CD184^+^/CD133^+^ NPCs) in arrayed format revealed one or more effects on proliferation (Ki-67; Fig. 6c and Supplementary Fig. 18a), neurogenesis (NPCs: CD184^+^/CD133^+^/CD271^−^; neurons: CD184^−^/CD44^−^/CD24^+^; Supplementary Fig. 18b) and/or gliogenesis (astrocytes: CD184^+^/CD44^+^; Supplementary Fig. 18c) that varied between genes. Likewise, a pooled CRISPR analysis in iCas9 cortical organoids confirmed effects on neurogenesis, again with variable effects between NDD genes (Supplementary Fig. 19).

To assess how loss of NDD-associated genes affects mitochondrial function, we performed a pooled CRISPR-KO screen using a nearly identical library (same backbone, guide density and control set) in the H1-iCas9 line. Transduced cells were differentiated into NPCs and iGLUTs by day 21, stained with the mitochondrial inner membrane potential-sensitive dye JC-1 and sorted by fluorescence-activated cell sorting (FACS) into high- (PE-high) and low- (FITC-high) membrane potential fractions, following amplicon sequencing to quantify gRNA representation in each fraction (Fig. 6d). Of the 15 KOs, ten resulted in elevated mitochondrial membrane potential in both NPCs and iGLUTs, and the remaining five caused cell-type-specific impacts on mitochondrial membrane potential. Pathway enrichment of the ten NDD genes that increased mitochondrial membrane potential revealed a convergence on chromatin remodeling complexes, microRNAs and transcription factors.

Given their strong phenotypes and unexpected enrichment of mitochondrial pathways, we selected three NDD KOs (*ASH1L*, *ARID1B*, *NRXN1*) for further testing of mitochondrial effects in arrayed format, using a platform with the ability to resolve dose-dependent changes in mitochondrial fragmentation following pharmacological insults (Supplementary Fig. 20). By high content imaging, we analyzed and quantified 1 × 10^4^ mitochondria per genotype, with morphological measurements taken for mitochondrial (TOMM20-positive) volume, surface area and sphericity (roundness) as well as total OXPHOS complex, within neuronal dendrites (MAP2-positive) of mature (day 21) iGLUTs. Among the three NDD KOs, *ARID1B* resulted in increased mitochondrial networking (indicated by decreased mitochondrial sphericity and increased branch length; one-way analysis of variance (ANOVA), Šidák’s, adjusted *P* = 0.0213 and *P* = 0.0081, respectively) concomitant with increased levels of OXPHOS proteins (one-way ANOVA, Šidák’s, adjusted *P* = 0.0024) (Fig. 6e and Supplementary Fig. 21a), overall consistent with increased mitochondrial efficiency. Second, we tested oxygen consumption using the Seahorse Cell Mito Stress test. NRXN1 KO resulted in increased coupled and maximal respiration in iGLUTs (one-way ANOVA, *P* < 0.05; Fig. 6f); increased mitochondrial reliance, in the absence of fused mitochondria, with elevated OXPHOS protein levels points to a possible metabolic overload due to reduced mitochondrial efficiency (Fig. 6e). In contrast, ARID1B and ASH1L KOs did not show significant changes in these Seahorse parameters (Supplementary Fig. 21b,c). Taken together, both ARID1B and NRXN1 KO neurons show evidence of increased mitochondrial activity, ARID1B-KO through enhanced fusion and elevated expression of OXPHOS complexes, and NRXN1 KO by increasing OXPHOS activity to meet ATP demands. As observed for neurogenesis in iNPCs, single-gene KO iGLUTs confirmed convergent effects on mitochondrial biology, finding distinct but related phenotypes between NDD genes.

### Pharmacological targeting of convergent genes reversed behavioral phenotypes in mutant zebrafish

**Fig. 7:**
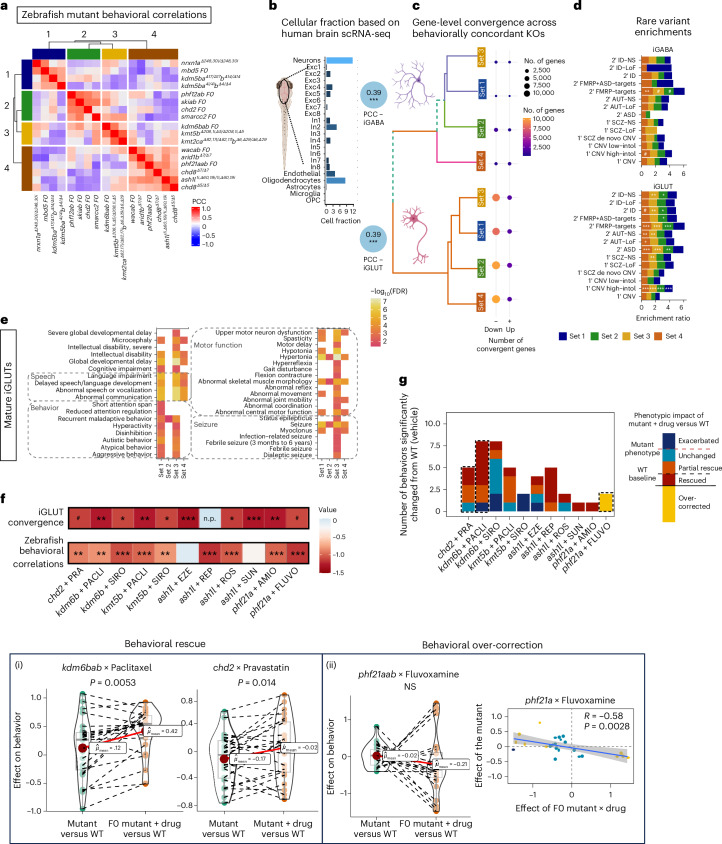
NDD gene mutants with shared behavioral phenotypes in zebrafish resolve unique and cell-type-specific gene-level convergent signatures and are rescued by predicted medications. **a**, NDD risk genes uniquely cluster based on sleep–wake/visual-startle behavioral responses in zebrafish mutants. set 1: *nrxn1a*, *mbd5*, *kdm5bab;* set 2: *phf12ab*, *skiab*, *chd2*, *smarcc2*; set 3: *kdm6bab*, *kmt5b*, *kmt2cab*; set 4: *wacab*, *arid1b*, *phf21aab*, *chd8*, *ash1l*. **b**, Gene expression in human mature iGLUTs and iGABAs correlates with expression in the zebrafish brain. Cellular deconvolution of WT larval zebrafish brain expression based on adult human single-cell brain reference identifying neurons as the largest proportion of cells in the fish brain. Gene expression in WT zebrafish brain significantly positively correlates with gene expression of mature iGLUTs (two-tailed Pearson’s correlation with Holm’s multiple testing correction; *R* = 0.39, *P*_Holm_ < 0.001) and iGABAs (*R* = 0.39, *P*_Holm_ < 0.001). **c**, For each of the four behaviorally defined sets, gene-level convergence (DEGs with significant and shared direction of effect across all NDD genes within each of the four sets (*P* value-based DEG meta-analysis (METAL), FDR-adjusted *P*_meta_ < 0.05, Cochran’s heterogeneity *Q*-test *P*_Het_ > 0.05)) is largely nonoverlapping between mature iGLUTs and iGABAs, with unique enrichments for common psychiatric risk gene targets. Numbers of convergent genes that are upregulated (+) or downregulated (−) for each NDD set are indicated. **d**, In both iGABAs and iGLUTs, all four behavioral sets were enriched for FMRP targets. Gene targets of neurodevelopmental rare variants were significantly enriched for convergent signatures only in mature iGLUTs; behavioral set 4 uniquely significantly enriched for secondary targets of ASD LoF variants and set 3 uniquely enriched for primary targets of SCZ nonsynonymous variants. **e**, In iGLUTs, NDD-related behaviors were enriched only in sets 1 and 3, with enrichments for language, speech and intellectual delays in sets 1, 3 and 4. All sets were enriched for seizure and hypertonia. **f**, Potential ‘rescue’ drugs for these four phenotypic groups were selected from enrichment scores using CMap and filtered for drugs included in a screen of 376 compounds for behavioral effects in zebrafish. Top candidates that were significantly negatively enriched for iGLUT convergence from CMap and negatively correlated with mutant behavioral features were tested in mutant lines representative of sets 2–4. n.p. indicates that the drug repaglinide was not present in the CMap dataset. Mutant × drug combinations were as follows: *chd2*^*Δ7/Δ7*^ × pravastatin; *kdm6bab* F0 × paclitaxel; *kdm6bab* F0 × sirolimus; *kmt5b*^Δ208,1i, Δ5/Δ208,1i, Δ5^ × paclitaxel; *kmt5b*^Δ208,1i, Δ5/Δ208,1i, Δ5^ × sirolimus; *ash1l*^1i, Δ60,19i/ 1i, Δ60,19i^ × ezetimibe; *ash1l*^1i, Δ60,19i/ 1i, Δ60,19i^ × repaglinide; *ash1l*^1i, Δ60,19i/ 1i, Δ60,19i^ × rosuvastatin; *ash1l*^1i, Δ60,19i/ 1i, Δ60,19i^ × sunitinib; *phf21aab* F0 × amiodarone; *phf21aab* F0 × fluvoxamine. **g**, For behaviors that were significantly different between mutant + DMSO and WT + DMSO (*P* < 0.06), we characterized the effect of the mutant × drug on behavior as exacerbated (**a**) (significant effect mutant + drug-versus-WT > significant effect mutant-versus-WT), unchanged (**b**) (significant effect mutant + drug-versus-WT = significant effect mutant-versus-WT), partial rescue (**c**) (significant effect mutant + drug-versus-WT < effect mutant-versus-WT), rescued (**d**) (significant effect mutant-versus-WT, no significant effect mutant + drug-versus-WT) or over-corrected (**e**) (mutant + drug-versus-WT opposite direction of significant effect mutant-versus-WT). All drugs reversed at least one dysregulated behavior except for sirolimus in *kmt5b*. (i) Comparison of the magnitude of effect (beta, *n* = 24 parameters) on behavior between the mutant + DMSO compared with mutant + drug groups shows rescue of select behavioral features in *kdm6b* and *chd2* mutants by paclitaxel (Shapiro–Wilk’s normality: *W* = 0.94301, *P* = 0.02121; two-sided Wilcoxon signed rank test statistic = 52, *P* = 0.0053, *n* = 24) and pravastatin (Shapiro–Wilk’s normality: *W* = 0.97587, *P* = 0.4203, two-sided paired Welch’s *t-*statistic = −3.533, *P* = 0.01394, *n* = 24), respectively. (ii) The *phf21a* mutant phenotype was strongly opposed by fluvoxamine (left: normality statistic = 0.93744, *P* = 0.01295; two-sided Wilcoxon signed rank test statistic = 196, *P* = 0.19; right: two-tailed Pearson’s correlation = −0.58, *P* = 0.0028, *n* = 24). In the box plots, the median (black line) and mean (red point) are shown. The lower and upper hinges correspond to 1st and 3rd quartiles. Upper and lower whiskers extend from the hinge to the largest or smallest value up to 1.5 × IQR. All data points are plotted individually. Illustrations in **b** created in BioRender; Townsley, K. https://biorender.com/rvk1zn2 (2026). AMIO, amiodarone; EZE, ezetimibe; FLUVO, fluvoxamine; PACLI, paclitaxel; PRA, pravastatin; REP, repaglinide; ROS, rosuvastatin; SIRO, sirolimus; SUN, sunitinib. Source data

By design, in vitro models substantially limit the complexity of the observed impact of NDD genes, lacking higher circuit-level effects. Towards applying molecular convergence in vitro to explore the mechanisms of phenotypic convergence in vivo, the convergence of sets of NDD genes was next explored on the basis of shared behavioral effects in zebrafish mutants (Fig. 7 and Supplementary Tables 7 and 8). A comprehensive in vivo high-throughput, automated behavioral analysis in larval zebrafish^41^ revealed clear stratification of NDD genes based on basic arousal and sensory processing behaviors (Fig. 7a and Supplementary Fig. 22). Given that zebrafish brain gene expression was significantly correlated with in vitro human-derived mature neurons (Fig. 7b and Supplementary Fig. 23), we asked whether behavioral stratification of NDD mutants in larval zebrafish can be attributed to molecular convergence. For 15 NDD genes for which we have matched behavioral and molecular analyses, zebrafish stable mutant lines and CRISPR F0 mutants were clustered based on 24 sleep–wake and visual-startle parameters, yielding four distinct clusters of genes: set 1 (*nrxn1a*, *mbd5*, *kdm5bab*), set 2 (*phf12ab*, *skiab*, *chd2*, *smarcc2*), set 3 (*kdm6bab*, *kmt5b*, *kmt2cab*) and set 4 (*wacab*, *arid1b*, *phf21aab*, *chd8*, *ash1l*) (Fig. 7a and Supplementary Data 3). Gene-level convergence between NDD genes in these sets was distinct (Supplementary Table 9), largely nonoverlapping between cell types and stronger in mature iGLUTs than mature iGABAs (Fig. 7c). Across behavioral sets, rare ASD, SCZ and intellectual disability LoF genes were enriched primarily in iGLUTs, with all sets converging on FMRP targets, highly intolerant copy number variants (CNVs) and ASD variants (Fig. 7d). Phenotypes related to DD, behavior and motor function showed unique enrichments by set, predominately in the iGLUTs, whereas all sets were enriched for seizure, hypertonia and abnormal skeletal muscle morphology (Fig. 7e). Candidate drugs predicted to reverse convergent genes (that is, drugs with anti-correlating transcriptomic signatures) in iGLUTs and iGABAs were prioritized from the 520 Connectivity Map (CMap)^45^ drugs with matched clinical and experimental zebrafish data. Top enriched drugs included antidepressants, antipsychotics and statins (Supplementary Data 2 and Supplementary Fig. 24a). Whereas some drugs were broadly predicted to reverse convergent signatures in three out of four NDD gene sets (for example, the antipsychotic perphenazine), others uniquely targeted specific sets (for example, naltrexone in set 2 iGLUTs, sirolimus in set 3 iGLUTs and valsartan in set 3 iGABAs). Sets 3 and 4 showed the greatest number of CMap enrichments. By considering existing pharmacological effects of the top drugs on zebrafish behavior^46^, some of the predicted drug reversers were shown to oppose effects on NDD-related phenotypes in zebrafish (Supplementary Fig. 24b). Yet, the direction of effect predicted based on transcriptomic convergence in human neurons did not always align with anti-correlating behavioral effects in zebrafish (for example, moxifloxacin, perphenazine).

The top negatively enriched drugs for iGLUT convergence from CMap and anti-correlating drugs predicted from a pharmaco-behavioral screen of 376 drugs in larval zebrafish^46^ were empirically tested in representative mutants from sets 2–4, which showed the strongest CMap enrichments (Fig. 7f). We determined whether the phenotypic impact of mutant × drug combinations led to partial rescue, rescue, over-correction or exacerbation of the mutant phenotype across significant arousal and startle behavioral parameters (Fig. 7g). Ten out of 11 drugs rescued at least one dysregulated behavioral parameter (Fig. 7g and Supplementary Fig. 24c–e). Paclitaxel robustly rescued behavioral parameters in *kdm6bab* F0 mutants and pravastatin partially and completely rescued select parameters in *chd2*^*Δ7/Δ7*^ mutants (Fig. 7g(i)), including nighttime sleep bouts in *kdm6bab* F0 mutants and responses to lights-ON stimuli in *chd2*^*Δ7/Δ7*^ mutants (Supplementary Fig. 24f(i),(ii)). Interestingly, we also observed over-correction of the *phf21aab* F0 mutant phenotype by fluvoxamine (Fig. 7g(ii)), such as increased sleep bouts that were significantly decreased following fluvoxamine treatment (Supplementary Fig. 24f(iii)). Taken together, in vivo behavioral profiling of NDD genes in zebrafish overlaps with in vitro-defined convergent networks and identifies pharmacological suppressors of specific behavioral phenotypes.

## Discussion

To investigate common pathways affected by loss of NDD risk genes, we targeted 23 NDD genes with roles predominantly in gene regulation using a pooled CRISPR-KO strategy. Transcriptomic convergence across NDD risk genes varied between the cell types of the brain, resolving more convergent targets and stronger convergent networks in mature glutamatergic neurons, where they were enriched not just for pathways with well-established links to ASD etiology (for example, gene regulation, synaptic biology), but also mitochondrial function^47^. Machine learning tools extended the analyses in silico to all known NDD genes, recapitulating observed enrichments for regulatory NDD genes, yet predicting even greater convergence for synaptic NDD genes, and unexpectedly suggesting that predominantly ASD genes converge in excitatory neurons whereas predominantly DD genes converge in inhibitory neurons. Finally, drugs predicted to reverse convergent signatures suppressed behavioral phenotypes in NDD gene mutant zebrafish.

While downstream effects of epigenetic NDD genes unexpectedly targeted mitochondrial genes in neurons, in fact, 5% of NDD cases meet diagnostic criteria for classic mitochondrial disorders^48^. Mitochondrial DNA mutations^49,50^, haplotypes^51^ and heteroplasmy^49,52^ have all been associated with NDD. Not only do mitochondrial mutations cause synaptic and behavioral phenotypes^53^, but multiple lines of human and animal evidence link NDDs to mitochondrial deficits and oxidative stress^54–59^, with neuronal and/or behavioral phenotypes reversed by antioxidant treatment^55,57–59^.

Perturbations of the same NDD genes resulted in different convergent networks across cell types. For example, KOs of NDD genes in human NPCs^18,34^, cerebral organoids^36–38^ and developing mouse^39^, tadpole^40^ and zebrafish^41^ brains reveal overlapping alterations in neurogenesis and developmental dynamics. Indeed, both regulatory and synaptic genes impact proliferation and patterning of progenitors (for example, *ARID1B*^60^, *CHD8*^36^, *NRXN1*^61^, *SYNGAP1*^62^), excitatory transmission by glutamatergic neurons (for example, *CHD8*^63,64^, *NRXN1*^65^, *SHANK3*^66^, *SYNGAP1*^67^) and inhibitory transmission by GABAergic neurons (for example, *ARID1B*^68^, *CHD8*^63^, *NRXN1*^69^, *SHANK3*^70^). Many NDD genes seem to have broad roles outside their annotated function; for example, some chromatin regulators (for example, *CHD8, CHD2* and *POGZ*) localize to microtubules in the centrosome^17^, mitotic spindle^18^ and cilia^19^. This observation connects the pleiotropic nature of many NDD genes and pathophysiological evidence linking multiple cell types and distinct cellular functions to NDD.

What explains phenotypic convergence between NDD genes with distinct annotated functions? The strength of convergence was most highly correlated to common clinical associations, biological annotations and co-expression patterns in the post-mortem brain. Critically, these factors are inter-dependent. ASD and DD risk genes differentially map onto human developmental brain co-expression networks^11^, with those most strongly implicated in DD enriched for expression in progenitor cells and immature neurons, and those in ASD in mature neurons^5^. Indeed, cellular identities and biological pathways are captured by patterns of gene co-expression^71,72^. Transcriptomic and epigenomic analyses of post-mortem brain from NDD cases likewise indicate convergent molecular signatures^73^ and subtypes of NDD^74^. Thus, we posit that shared clinical and phenotypic effects of distinct NDD genes in fact reflect the patterns of co-expression in the developing brain.

Personalized medicine seeks to tailor treatments to individual patients^75^; for example, patients with cancer^76^ and monogenic disease^77^ with specific genetic mutations receive targeted treatments. Previous efforts to classify genes that predict NDD clinical features or treatment response applied gene ontology^4,5^ or differential neurodevelopmental KO effects in vitro^34^ or in vivo^40^. Here we proposed to stratify risk genes based on convergent molecular impacts in human neurons. Our overarching hypothesis, in doing so, was that by resolving shared downstream gene targets between multiple NDD genes, we might inform a precision-medicine-based approach that did not necessarily need to target risk genes one-at-a-time. Although convergent networks did not predict behavioral stratification of zebrafish mutants, they did inform drug prediction, with 10 out of 11 drugs tested found to ameliorate at least one mutant behavioral phenotype in vivo. This ability to reverse, rather than prevent, a behavioral phenotype indicates that targeting convergent networks in post-mitotic neurons may represent a clinically actionable neurodevelopmental window that persists through symptom onset. The extent to which convergent downstream targets, whether associated with risk or resilience, can be manipulated to prevent or ameliorate NDD signatures and phenotypes warrants future investigation.

Although rare LoF NDD gene mutations tend to confer large effects in the individuals who carry them, the small effects of common variants account for much of the genetic risk for NDD at the population level^2^. The differences in expressivity and incomplete penetrance of high-effect-size rare variants are frequently attributed to diversity across polygenic backgrounds^78^; in vitro, NDD gene effects are indeed influenced by the individual genomic context^36^. In psychiatry, common genetic variants are more associated with cross-disorder behavioral dimensions^79^ and rare variants with co-occurring intellectual disability^80^. Common risk variants interact with rare mutations to determine individual-level liability in ASD^81^, DD^82^, SCZ^83,84^ and epilepsy^85^. Our results, highlighting that convergence downstream of NDD gene effects is enriched for cross-disorder GWAS variants and rare LoF genes, inform pleiotropy of genetic risk for psychiatric disorders. Moving forward, we argue that it is critical that empirical functional genomic studies systematically consider the impact of common and rare variants together, including screening the impact of LoF genes in hiPSC lines derived from donors with high and low polygenic risk scores^86^. Intriguingly, even susceptibility to environmental risk factors for NDD (for example, valproic acid) seems to be mediated by genetic background^87^. Deeper phenotypic characterization of NDD effects across donors will be critical in determining how complex genetic (or environmental) interactions shape cellular phenotypes, circuit function and human behavior in the clinic.

In the post-mortem brain, NDD gene signatures are not just associated with downregulation of co-expression modules involving synaptic signaling^88^, but also with upregulation of microglial and astrocyte gene modules^89,90^. The extent to which increased neuroimmune activity in NDD is a response to cellular or environmental sources of inflammation, or indicative of a role for glia cells in risk, is unclear; evidence supports both possibilities. Consistent with a model of maternal immune activation during neurodevelopment^91^, glucocorticoids and inflammatory cytokines perturb the expression of psychiatric risk genes^92,93^, altering the regulatory activity of psychiatric risk loci^94^ and interfering with neuronal maturation in brain organoids^95^. Yet, in vivo analysis of NDD genes in zebrafish revealed global increases in microglia^41^ and in vitro screening in human microglia uncovered roles in endocytosis and uptake of synaptic material^96^. Indeed, given the reciprocal relationships between neuronal activity and glial function, epigenetic state and gene expression^97–99^, it seems probable that both cell-autonomous and non-cell-autonomous effects underlie and/or exacerbate NDD gene effects.

In summary, we demonstrate that convergent effects of NDD risk genes vary between cell types. Our analyses suggest that clinical convergence between regulatory and synaptic genes in the etiology of NDD is driven more so by co-expression patterns of risk genes than by direct regulation of epigenetic genes on synaptic targets. If the convergence of multifold risk genes on a smaller number of shared molecular pathways indeed explains how genetically heterogeneous mutations result in similar clinical features, then genetic stratification of cases will inform novel therapeutic targets. We predict that such individualized points of therapeutic intervention may be most effective when targeting mature glutamatergic neurons, which not only harbor the strongest convergent effects but also represent a therapeutic window that is actionable after diagnosis.

## Methods

### Statement of ethics

Yale University Institutional Review Board waived ethical approval for this work. Ethical approval was not required because the hiPSC lines, lacking association with any identifying information and widely accessible from a public repository, are thus not considered to be human subject research. Post-mortem brain data are similarly lacking identifiable information and are not considered human subject research.

All procedures involving zebrafish were conducted in accordance with Institutional Animal Care and Use Committee (IACUC; protocol no. 2024-20054) regulatory standards at Yale University.

### Generation of neural cells

Informed consent was obtained at the National Institute of Mental Health (NIMH), under the review of their Internal Review Board. hiPSC work was reviewed by the Internal Review Board of the Icahn School of Medicine at Mount Sinai as well as by the Embryonic Stem Cell Research Oversight Committee at the Icahn School of Medicine at Mount Sinai and Yale University. Fibroblasts were genotyped by IlluminaOmni 2.5 bead chip genotyping^100,101^, PsychChip^102^ and exome sequencing^102^; hiPSCs^102^ were validated by G-banded karyotyping (Wicell Cytogenetics) and genome stability monitored by Infinium Global Screening Array v3.0 (lllumina); single nucleotide polymorphism (SNP) genotype was inferred from all RNA sequencing (RNA-seq) data using the Sequenom SURESelect Clinical Research Exome and Sure Select V5 SNP lists to confirm that neuron identity matched donor. All hiPSCs are available at the Rutgers University Cell and DNA Repository (study 160) NIMH Repository and Genomic Resource is in the process of transferring operations from Rutgers University to Coriell Institute for Medical Research. For urgent requests, please contact NIMH.genomics.resources@mail.nih.gov.

Control hiPSCs were cultured in StemFlex media (Gibco, cat. no. A3349401) supplemented with Antibiotic-Antimycotic (Gibco, cat. no. 15240062) on Geltrex-coated plates (Gibco, cat. no. A1413302). Cells were passaged at 80–90% confluence with 5 mM EDTA (Life Technologies, cat. no. 15575-020) for 3 min at room temperature. EDTA was aspirated and cells dissociated in fresh StemFlex media. Media was replaced every 48–72 h for 4–7 d until the next passage.

Transient transcription factor overexpression from stable clonal hiPSCs was used to induce control hiPSCs to iNPCs (here SNaPs)^26^, iGLUTs^27^ and iGABAs^28^. iNPCs are rapidly generated by 48-h induction with *NGN2*^26,103^. iGLUTs are induced via transient overexpression of *NGN2*, and are >95% glutamatergic neurons, robustly express excitatory genes and show spontaneous excitatory synaptic activity by 3–4 weeks in vitro^25,27,61,65,66,104–110^. iGABA neurons are induced via transient overexpression of *ASCL1* and *DLX2*, and are >95% GABAergic neurons, robustly express inhibitory genes and show spontaneous inhibitory synaptic activity by 5–6 weeks^28,69,106,111,112^. iNPCs, iGLUTs and iGABAs express most NDD genes, including all genes prioritized herein^25^.

We transduced hiPSCs from two control donors (553-3, karyotypic XY; 3182-3, karyotypic XX) with lentiviral *pUBIQ-rtTA* (Addgene, cat. no. 20342) and *tetO-NGN2-eGFP-NeoR* (Addgene, cat. no. 99378) for iNPCs and iGLUTs, or *pUBIQ-rtTA* (Addgene, cat. no. 20342), *tetO-ASCL1-PuroR* (Addgene, cat. no. 97329) and *tetO-DLX2-HygroR* (Addgene, cat. no. 97330) for iGABAs. Following transduction by spinfection at 1,000*g* for 1 h at 37 °C, hiPSCs were subjected to 48-h antibiotic selection (1 mg ml^−1^ neomycin G418 (Thermo, cat. no. 10131027), 0.5 µg ml^−1^ puromycin (Thermo, cat. no. A1113803) and/or 250 µg ml^−1^ hygromycin (Thermo, cat. no. 10687010), and then clonalized by expansion from single colonies. Ultimately, clonal and inducible iNPC/iGLUT 3182-3-clone5 (XX) and iGABA 553-3-clone34 (XY) hiPSCs were validated for lentiviral genome integration by PCR, doxycycline-induced transcription factor expression by quantitative PCR, and robust and consistent neuronal induction confirmed by RNA-seq and immunocytochemistry for relevant cell-type markers. Analyses throughout reflect data from iGLUT 3182-3-clone5 (iNPC, day 7 iGLUT and day 21 iGLUT) and iGABA 553-3-clone34 (day 36 iGABA).

#### iNPCs

At 0 days in vitro (DIV0), 3182-3-clone5 hiPSCs were dissociated and plated at 1.5 × 10^6^ cells per well onto Geltrex-coated six-well plates (1:250 dilution coating) in SNaP Induction Media (DIV0): DMEM/F12 with Glutamax (ThermoFisher, cat. no. 11320082), Glucose (0.3% v/v), N2 Supplement (1:100, ThermoFisher, cat. no. 17502048), doxycycline (2 μg ml^−1^; Sigma-Aldrich, cat. no. D9891), LDN-193189 (200 nM; Stemgent, cat. no. 04-0074), SB431542 (10 μM; Tocris, cat. no. 1614) and XAV939 (2 μM; Stemgent, cat. no. 04-00046) supplemented with 25 ng ml^−1^ Chroma I ROCK2 Inhibitor. After 24 h, DIV2, cells were fed with Selection Media: DMEM/F12 with Glutamax, Glucose (0.3% v/v), N2 Supplement (1:100), doxycycline (2 μg ml^−1^), Geneticin (0.5 mg ml^−1^; Thermofisher, cat. no. 10131035), LDN-193189 (100 nM), SB431542 (5 μM) and XAV939 (1 μM). At 48 h post induction (DIV2), SNaPs were dissociated with Accutase for 10 min at 37 °C, quenched in DMEM, pelleted at 800*g* for 5 min and replated at 1.5 × 10^6^ cells per well onto Geltrex-coated six-well plates in SNaP Selection Media supplemented with Geneticin (0.5 mg ml^−1^). After 16–18 h (DIV3), medium was switched to SNaP maintenance Medium: DMEM/F12 with Glutamax, Penn/Strep (1:100), MEM-NEAA (1:100; Life Technologies, cat. no. 10370088), B27 minus Vitamin A (1:50; Life Technologies, cat. no. 12587010), N2 Supplement (1:100; Life Technologies, cat. no. 17502048), recombinant human EGF (10 ng ml^−1^; R&D Systems, cat. no. 236-EG-200), recombinant human basic FGF (10 ng ml^−1^; Life Technologies, cat. no. 13256029), Geneticin (0.5 mg ml^−1^) and Chroman I (25 ng ml^−1^). Cells were fed every 48 h with SNaP maintenance medium lacking Chroman I and Geneticin. Cells were dissociated and seeded weekly at a density of 1.25–1.5 × 10^6^ cells per well onto Geltrex-coated six-well plates until NPC morphology was observed and persistent. Cells were expanded and cryofrozen.

#### DIV7 iGLUTs

The 3182-3-clone5 iNPCs were thawed and seeded at 1 × 10^6^ cells per well onto Geltrex-coated 12-well plates. *NGN2* expression was induced with doxycycline (2 μg ml^−1^) for 24 h (DIV0) with antibiotic selection for 48 h (DIV1-3) in SNaP maintenance medium. At DIV4, SNaPs were dissociated with Accutase, switched into Neuronal Medium (Brainphys (Stemcell, cat. no. 05790), Glutamax (1:100), Sodium Pyruvate (1 mM), Anti-Anti (1:100), N2 (1:100), B27 without vitamin A (1:50), BDNF (20 ng ml^−1^; R&D, cat. no. 248-BD-025), GDNF (20 ng ml^−1^; R&D, cat. no. 212), dibutyryl cAMP (500 μg ml^−1^; Sigma, cat. no. D0627), L-ascorbic acid (200 μM; Sigma, cat. no. A4403), Natural Mouse Laminin (1.2 μg ml^−1^; Thermofisher, cat. no. 23017015)) and seeded in Geltrex-coated (1:120 dilution coating) 12-well plates. Medium was changed every 24 h until DIV7 collection.

#### D21 iGLUTs

hiPSCs were collected in Accutase (Innovative Cell Technologies, cat. no. AT-104) for 5 min at 37 °C, dissociated into a single-cell suspension, quenched in DMEM, pelleted via centrifugation for 5 min at 1,000*g*, resuspended in StemFlex containing 25 ng ml^−1^ Chroma I ROCK2 Inhibitor and 2.0 μg ml^−1^ doxycycline (DIV0), seeded 1 × 10^6^ cells per well onto Geltrex-coated six-well plates (1:250 dilution coating) and incubated overnight at 37 °C. The next day, DIV1, hiPSCs were subjected to 48-h antibiotic selection by medium replacement with Induction Media: DMEM/F12 (Thermofisher, cat. no. 10565018), Glutamax (1:100; Thermofisher, cat. no. 10565018), N2 (1:100; Thermofisher, cat. no. 17502048), B27 without vitamin A (1:50; Thermofisher, cat. no. 12587010), Antibiotic-Antimycotic (1:100) with 1.0 μg ml^−1^ doxycycline and 0.5 mg ml^−1^ Geneticin. At DIV3, cells were treated with 4.0 μM cytosine-β-D-arabinofuranoside hydrochloride (Ara-C) and 1.0 μg ml^−1^ doxycycline to arrest proliferation and eliminate non-neuronal cells in the culture. At DIV4, immature neurons were dissociated with Accutase and 5 units per ml of DNAse I at 37 °C for 7–10 min, quenched in DMEM, centrifuged for 5 min at 400*g*, resuspended in 25 ng ml^−1^ Chroma I ROCK2 Inhibitor, 1.0 μg ml^−1^ doxycycline and 4.0 μM Ara-C, and switched to Neuron Medium: Brainphys (Stemcell, cat. no. 05790), Glutamax (1:100), Sodium Pyruvate (1 mM), Anti-Anti (1:100), N2 (1:100), B27 without vitamin A (1:50), BDNF (20 ng ml^−1^; R&D, cat. no. 248-BD-025), GDNF (20 ng ml^−1^; R&D, cat. no. 212), dibutyryl cAMP (500 μg ml^−1^; Sigma, cat. no. D0627), L-ascorbic acid (200 μM; Sigma, cat. no. A4403), Natural Mouse Laminin (1.2 μg ml^−1^; Thermofisher, cat. no. 23017015), and seeded at 7 × 10^5^ cells per well onto Geltrex-coated (1:60 dilution coating) 12-well plates and incubated overnight at 37 °C. The next day, DIV6, Chroman I was removed from culture and Ara-C lowered to 2.0 μM with a full Neuronal Medium change. At DIV7, a full Neuronal Medium change was performed to remove doxycycline and Ara-C from culture, to allow for antibiotic-resistant genes silencing. From DIV7 onwards, half Neuronal Medium changes were performed every 72–96 h until mature DIV21 for collection.

#### DIV36 iGABAs

hiPSCs were collected in Accutase (Innovative Cell Technologies, cat. no. AT-104) for 5 min at 37 °C, dissociated into a single-cell suspension, quenched in DMEM, pelleted via centrifugation for 5 min at 1,000*g*, resuspended in StemFlex containing 25 ng ml^−1^ Chroma I ROCK2 Inhibitor and 2.0 μg ml^−1^ doxycycline (DIV0), seeded at 1.5–2 × 10^6^ cells per well onto Geltrex-coated six-well plates (1:250 dilution coating) and incubated overnight at 37 °C. The next day, DIV1, hiPSCs were subjected to 48-h antibiotic selection by medium replacement with Induction Media: DMEM/F12 (Thermofisher, cat. no. 10565018), Glutamax (1:100; Thermofisher, cat. no. 10565018), N2 (1:100; Thermofisher, cat. no. 17502048), B27 without vitamin A (1:50; Thermofisher, cat. no. 12587010), Antibiotic-Antimycotic (1:100) with 1.0 μg ml^−1^ doxycycline, 1.0 μg ml^−1^ puromycin (Sigma, cat. no. P7255) and 250 μg ml^−1^ hygromycin (Sigma, cat. no. 10687010). At DIV3, cells were treated with 4.0 μM Ara-C and 1.0 μg ml^−1^ doxycycline to arrest proliferation and eliminate non-neuronal cells in the culture. At DIV5, immature neurons were dissociated with Accutase and 5 units per ml of DNAse I at 37 °C for 7–10 min, quenched in DMEM, centrifuged for 5 min at 400*g*, resuspended in 25 ng ml^−1^ Chroma I ROCK2 Inhibitor, 1.0 μg ml^−1^ doxycycline and 4.0 μM Ara-C, and switched to Neuron Medium: Brainphys (Stemcell, cat. no. 05790), Glutamax (1:100), Sodium Pyruvate (1 mM), Anti-Anti (1:100), N2 (1:100), B27 without vitamin A (1:50), BDNF (20 ng ml^−1^; R&D, cat. no. 248-BD-025), GDNF (20 ng ml^−1^; R&D, cat. no. 212), dibutyryl cAMP (500 μg ml^−1^; Sigma, cat. no. D0627), L-ascorbic acid (200 μM; Sigma, cat. no. A4403) and Natural Mouse Laminin (1.2 μg ml^−1^; Thermofisher, cat. no. 23017015), and seeded at 7 × 10^5^ cells per well onto Geltrex-coated (1:60 dilution coating) 12-well plates and incubated overnight at 37 °C. The next day, DIV6, Chroman I was removed from culture and Ara-C lowered to 2.0 μM with a full Neuronal Medium change. At DIV7, a full Neuronal Medium change was performed to remove doxycycline and Ara-C from culture, to allow for antibiotic-resistant genes silencing. From DIV7 onwards, half Neuronal Medium changes were performed every 72–96 h until mature DIV36 for collection.

### CRISPR-KO gRNA library design (Thermofisher) and validation

From the 102 highly penetrant LoF gene mutations associated with ASD (58 gene expression regulation, 24 neuronal communication genes, 9 cytoskeletal genes and 11 multifunction genes)^4^, gene ontology and primary literature research identified 26 epigenetic modifiers specifically involved in chromatin organization, rearrangement and modification. ASD gene expression (RNA-seq reads per kilobase million (RPKM) in iGLUTs) was plotted against significance of ASD association (transmission and de novo association analysis (TADA) FDR values), to ensure selection of genes with the highest expression and highest clinical association. Gene expression was confirmed across development in the brain (BrainSpan^113^), and in bulk RNA-seq and scRNA-seq. Twenty-one epigenetic modifiers (*ASH1L*, *ASXL3*, *ARID1B*, *CHD2*, *CHD8*, *CREBBP*, *KDM5B*, *KDM6B*, *KMT2C*, *KMT5B*, *MBD5*, *MED13L*, *PHF12*, *PHF21A*, *POGZ*, *PPP2R5D*, *SETD5*, *SIN3A*, *SKI*, *SMARCC2*, *WAC*) as well as two transcription factors with putative roles as chromatin regulators (*FOXP2*, *BCL11A*) were selected. Gene regulatory transcription factors, general transcription factors and DNA replication genes were excluded. Three extensively studied synaptic genes (*NRXN1*, *SCN2A*, *SHANK3*) with roles in ASD were included as positive controls and three under-explored genes for ASD roles in neuronal communication genes (*ANK3*, *DPYSL2*, *SLC6A1*) were also included in the library.

Individual DNA samples from glycerol stocks of Invitrogen LentiArray Human CRISPR Library gRNAs-PuroR (ThermoFisher, cat. no. A31949) (3–4 individual gRNAs per gene, Supplementary Table 1) were prepared using GeneJET Plasmid Miniprep Kit (K0503) and pooled at an equimolar ratio and a fivefold ratio of scramble control gRNA plasmid. Library quality was confirmed by restriction enzyme digest (10x Cutsart NEB) and agarose gel purification using QIAquick Gel Extraction Kit (cat. no. 28706) to check library purity, followed by MiSeq for gRNA count distribution. Based on the abundance of gRNAs from MiSeq, four NDD gene targets were highly unlikely to be resolved in the final experiments (*POGZ*, *PP2R5D*, *SHANK3*, *SLC6A1*) and three with low abundance were less likely to be resolved (*SCNA2*, *FOXP2*, *DYPSL2*).

Lentiviral Cas9v2-HygroR (Addgene, cat. no. 98291) and pooled LentiArray-gRNA-PuroR CRISPR-KO Library were packaged as high-titer lentiviruses (Boston Children’s Hospital Viral Core) and experimentally titrated in each cell type. The highest viable multiplicity of infection (MOI) was used for Cas9v2 and MOI < 0.5 for the lentivirus gRNA pool library.

#### CRISPR and gRNA delivery

Lentiviral Cas9v2-HygroR (Addgene, cat. no. 98291) transduction of iNPCs, day 4 (iGLUTs) or day 5 (iGABAs), occurred via spinfection (1 h at 1,000*g*) and was followed by 72 h of hygromycin (250 μg ml^−1^) (except for iGABAs, which express inducible hygromycin resistance at this stage). Pooled Invitrogen LentiArray Human gRNA-PuroR CRISPR-KO Library gRNAs (ThermoFisher, cat. no. A31949) (MOI 0.3–0.5) were transduced via spinfection 3 d before collection (for example, day 4 for day 7 iGLUTs, day 18 for day 21 iGLUTs, day 33 for day 36 iGABAs), with fresh medium containing puromycin (1 μg ml^−1^) added 16–24 h post transduction of gRNAs. For mature iGLUTs and iGABAs, as doxycycline was removed from medium at DIV7, and by DIV18 neurons had lost transcription factor-linked antibiotic resistance, at 24 h post transduction (DIV19 or DIV34) puromycin (1 μg ml^−1^) and hygromycin (250 μg ml^−1^) were added to media for 48-h of antibiotic selection before collection.

### Dissociation of different neural cell types to single cells for scRNA-seq assays

Cells were dissociated 72 h post gRNA library delivery for single-cell sequencing, as iNPCs, DIV7 and DIV21 iGLUTs, or DIV36 iGABAs, as follows:

iNPCs and DIV7 iGLUTs were dissociated in Accutase for 5 min at 37 °C, washed with DMEM/10%FBS, centrifuged at 1,000*g* for 5 min, gently resuspended and counted.

DIV21 iGLUTs and DIV36 iGABAs were dissociated with papain. Papain was pre-warmed (39 °C) for 30 min in HBSS (ThermoFisher, cat. no. 14025076), HEPES (10 mM, pH 7.5), EDTA (0.5 mM) and papain (0.84 mg ml^−1^; Worthington-Biochem, cat. no. LS003127). The cells were washed with PBS-EDTA (0.5 mM) and 300 μl of papain solution and 5 units of DNAse I were added per well of a 12-well plate and incubated at 37 °C for 10–15 min, 125 rpm. Dissociation was quenched with DMEM-10%FBS. Detached neurons were broken by gentle manual pipetting, pelleted at 600*g* for 5 min, resuspended in DMEM-10%FBS, filtered through a cell strainer and counted and submitted for 10X sequencing.

Cells were loaded into 10X in four lanes per cell type, targeting 20,000 cells per lane for a total of ~80,000 targeted cells per cell type. scRNA-seq was performed at Yale Genomics Core with the 10X single-cell 5′ v2 HT with CRISPR barcode kit.

### Bulk RNA-seq and CRISPR editing efficiency evaluation

The H1 hESC line with iCas9 (NIHhESC-10-0043), generously provided by the Huangfu Lab, was used to assess the editing efficiency of the gRNAs^44,114^ and to conduct the mitochondrial pooled and arrayed experiments. NPCs were generated using the dual SMAD inhibition approach per the STEMdiff SMADi Neural Induction Kit protocol (STEMCell Technologies, cat. no. 08581).

To validate gene KO, NPCs were transduced with LV particles carrying four gRNAs per target gene. After 48 h of selection with 1 µg ml^−1^ puromycin, Cas9 expression was induced by adding dox at 2 µg ml^−1^ for 72 h. Following induction, cells were collected for bulk RNA-seq. Total RNA was extracted using TRIzol reagent (Invitrogen). PolyA RNA-seq library preparation and sequencing were conducted at the Yale Center for Genomic Analysis (YCGA). Raw fastq files were quality-checked (FastQC; RRID:SCR_014583), then mapped to human genome reference hg38 (STAR^115^). gRNA-targeted loci for each sample were extracted (SAMtools^116^; RRID:SCR_002105). Variation/small insertion/deletion at the site of interest and mutation efficiency at corresponding loci were called (CrispRVariants R package^117^), after excluding possible germline variants from Cas9-noninduced samples.

NDD gene effects were resolved either (1) relative to a nontargeting control in the context of iCas9, owing to administration of doxycycline (for example, mitochondrial assays), or (2) across isogenic conditions expressing targeting gRNA, with the comparison being whether iCas9 expression was induced by administration of doxycycline (for example, replication assays).

### Proliferation and neurogenesis analysis

For proliferation analysis using Ki-67, NPCs were seeded into 24-well plates and either treated with doxycycline (induced) to activate Cas9 or left untreated (uninduced). The cells were cultured for 7 d, representing approximately three NPC generations. On day 7, cells were collected, and ~1 × 10^6^ cells were stained with Ki-67-FITC (cat. no. 130-117-803, Miltenyi Biotec) using the Foxp3/Transcription Factor Staining Buffer Set (cat. no. 00-5523, Invitrogen), following the manufacturer’s protocol.

To evaluate the effects of gene KOs on neurogenesis and gliogenesis, transduced NPC-iCas9 lines were spontaneously differentiated into human cortical neurons and glial cells. Briefly, 1 × 10^6^ cells were seeded in GelTrex-coated (1:5) six-well plates and cultured in complete neuronal media containing BrainPhys Neuronal Medium, Glutamax (100X), Sodium Pyruvate (100 mM), B27 (-RA) supplement (×50), N2 (×100), Anti-Anti (×100), Natural Mouse Laminin (1 mg ml^−1^), dbcAMP (500 mg ml^−1^), L-ascorbic acid (200 µM), BDNF (20 µg ml^−1^) and GDNF (20 µg ml^−1^). Medium was refreshed every 3 d. On day 25, cells were collected and stained for FACS analysis using surface markers previously described^118^ to differentiate NPCs (CD184^+^/CD44^−^/CD24^+^), neurons (CD184^−^/CD44^−^/CD24^+^) and glia (CD184^+^/CD44^+^). CD271, a marker for mesenchymal stem cells, was excluded from the original panel as NPCs were pre-purified via FACS using CD133^+^/CD184^+^/CD271^−^ markers before differentiation. A minimum of 50,000 cells per gate were acquired using a BD LSRFortessa Cell Analyzer at the Yale Flow Cytometry Core. Flow cytometry data were analyzed (FlowJo v10.10 Software; RRID:SCR_008520; BD Life Sciences).

The full protocol is available from protocols.io via 10.17504/protocols.io.j8nlkyew1g5r/v1.

All statistical analyses for flow cytometry assessment were conducted (GraphPad Prism version 9.5.1 (528) for macOS; RRID:SCR_002798; GraphPad Software). Each well was treated as an independent replicate. Differences between KO (induced) and control (uninduced) groups were assessed by comparing the median fluorescence intensity of the target fluorophore using an unpaired *t*-test with Welch correction to account for individual group variance. Multiple comparisons were corrected using the FDR method with a two-stage step-up procedure (Benjamini, Krieger and Yekutieli) at an FDR threshold of 5%.

### FACS analysis of mitochondrial membrane potential and CRISPR screen read-out via amplicon sequencing

For our mitochondrial assays, we used a nearly identical library (same backbone, guide density and control set) screened exclusively in the H1 inducible Cas9 (H1-iCas9) hPSC line. Mitochondrial inner membrane potential was measured in H1-iCas9, following differentiation to NPCs or iGlut on day 21. Cells were collected, counted and aliquoted at 1 × 10^6^ cells per sample. JC-1 dye (MitoProbe JC-1 Assay Kit; Invitrogen, cat. no. M34152) was dissolved in DMSO at a stock concentration of 200 µM and added to each sample to achieve a final concentration of 2 µM, then incubated for 30 min at 37 °C in 5% CO_2_. A 50 µM CCCP control was included to induce complete mitochondrial depolarization. After staining, cells were washed once in their respective culture medium, resuspended in FACS buffer (Invitrogen eBioscience Staining Buffer, cat. no. 00422226) and analyzed immediately on a ThermoFisher ‘Bigfoot’ spectral cell sorter using 488-nm excitation with 525/50-nm (FITC) and 585/40-nm (PE) emission filters. Debris and doublets were excluded by forward/side scatter gating, and CCCP-treated samples were used to define FITC and PE gates. Approximately 1 × 10^6^ events per sample were recorded. Cells were then pelleted (300*g*, 5 min) and genomic DNA extracted using the Qiagen DNeasy Blood & Tissue Kit (cat. no. 69504).

Unique molecular identifier (UMI)-tagged amplicon libraries were generated in three PCR steps. In PCR-1, genomic DNA was amplified with Platinum II Hot-Start PCR Master Mix (Invitrogen, cat. no. 14000012) and UMI-containing primers (Forward: 5′-ACACTCTTTCCCTACACGACGCTCTTCCGATCTACGTGACGTAGAAAGTAATAATTTCTTGGGT-3′; Reverse: 5′-GTGACTGGAGTTCAGACGTGTGCTCTTCCGATCTN(25)NNNNNNNNNACTCGGTGCCACTTTTTCAA-3′) under the following conditions: 94 °C for 2 min; 4–6 cycles of 98 °C for 5 s, 60 °C for 15 s, 60 °C for 30 s. The resulting ~180-base pair (bp) products were purified and concentrated using the Zymo DNA Clean & Concentrator-5 kit (cat. no. D4013) and eluted in 10 µl of nuclease-free water (ThermoFisher, cat. no. AM9938). In PCR-2, purified product was amplified with adaptor primers (Forward: 5′-ACACTCTTTCCCTACACGACGCTCTTCCGATCT-3′; Reverse: 5′-GTGACTGGAGTTCAGACGTGTGCTCTTCCGATCT-3′) for 22 cycles under identical cycling conditions in a ~20-µl reaction. A seven-cycle indexing PCR (PCR-3) was performed by the sequencing facility at YCGA before sequencing. Final libraries were sequenced on an Illumina NovaSeq platform (paired-end 150 bp, 5 million reads per sample).

Flanking sequences on both sides of each gRNA were trimmed using BBDuk, and reads were then mapped to gRNA reference sequences and counted (MAGeCK^119^; RRID:SCR_025016). Raw counts for each gRNA were normalized to counts of scrambled gRNA. Abundance of each target gene was then calculated by summing of all gRNAs targeting that gene. The log_2_-transformed FCs of gRNA-target abundance were compared between PE-high samples and FITC-high samples.

The full protocol is available from protocols.io via 10.17504/protocols.io.rm7vz98brgx1/v1.

### Immunostaining

Cells were fixed with fixative solution (4% sucrose and 4% paraformaldehyde prepared in Dulbecco’s PBS (DPBS)) for 10 min at room temperature. Following this, cells were washed twice with DPBS and incubated in blocking solution (2% normal donkey serum prepared in DPBS) supplemented with 0.1% Triton for 2 h at room temperature. After this, cells were incubated overnight at 4 °C in the primary antibody solution prepared in blocking solution. Cells were washed three times with DPBS, incubated at room temperature in secondary antibody prepared in blocking solution, then washed three times with DPBS. In the second wash, cells were incubated in DBPS supplemented with DAPI (Sigma D9542,1 μg ml^−1^) for 2 min at room temperature.

**Table:** 

Antibody	Species	Vendor	Cat. no.	RRID	Dilution
Anti-MAP2	Chicken	Invitrogen, Abcam	PA1-10005, ab5392	AB_1076848, AB_2138153	1:1,000
Anti-Nestin	Rabbit	Millipore	ABD69	AB_2744681	1:200
Anti-vGLUT1	Rabbit	Synaptic Systems	135-303	AB_887875	1:200
Anti-GABA	Rabbit	Sigma-Aldrich	A2052	AB_477652	1:200
TOMM20	Mouse	Santa Cruz Biotechnology	sc-17764	AB_628381	1:200
Total OXPHOS	Rabbit	Abcam	AB-317270	not available	1:500
Anti-mouse	Donkey	Jackson ImmunoResearch	715-605-151	AB_2340863	1:500
Anti-rabbit	Donkey	Jackson ImmunoResearch	711-545-152	AB_2313584	1:500
Anti-chicken	Donkey	Jackson ImmunoResearch	715-605-150, 703-545-155	AB_2340862, AB_2340375	1:500

Fixed cultures were acquired using a DragonFly Confocal Dual Spinning Disk confocal microscope, at ×60 magnification and 1.4 numerical aperture. All images were acquired with a fixed laser intensity and exposure time across experimental conditions. Four images were acquired per well, and 4–10 wells were acquired per experimental condition. Each well represents a biological replicate and statistical data point. Therefore, each replicate represents hundreds of μm^2^ of neuronal area and tens of thousands of individual mitochondria.

Mitochondrial morphology features were determined using the Surface module of Imaris 10.2 (RRID:SCR_007370). Likewise, OXPHOS complex features were determined using the Surface module of Imaris 10.2. The Volume, Area and Sphericity features of the Surface module were selected for analysis. Mitochondrial networking features were determined using published, open-source methods^120^. A one-way ANOVA with a Šidák’s multiple comparisons test was performed on data with GraphPad Prism 10.

To validate robustness and sensitivity of the microscopy assay, we treated day 14 iGluts overnight with carbonyl cyanide 4-(trifluoromethoxy) phenylhydrazone (FCCP) (Sigma-Aldrich, cat. no. SML2959) at 5 μM, 10 μΜ and 50 μΜ doses. Following this, we conducted the immunostaining, mitochondrial structural analysis and statistical analyses outlined above.

### Seahorse XF Mito Stress Test

Day 5 iGLUTs were plated at 1.65 × 10^5^ cells per well in XF24 microplates (Agilent, cat. no. 100777-004) and cultured to day 21. At 1 h before measurement, growth medium was removed, leaving 50 µl per well, and replaced with 1 ml of pre‑warmed Seahorse XF DMEM (Agilent, cat. no. 103575-100) supplemented with 25 mM glucose (Agilent, cat. no. 103577-100) and 0.23 mM pyruvate (Agilent, cat. no. 103578-100). Plates were equilibrated for 1 h at 37 °C in a non‑CO_2_ incubator. Immediately before the assay, the medium was replaced with 500 µl of fresh assay buffer. Oxygen‑consumption rate was recorded on a SeahorseXFe24 Analyzer (Agilent) using the standard Mito Stress Test. The program consisted of three sequential injections—1.5 µM oligomycin (Sigma-Aldrich, cat. no. 75351), 1.5 µM FCCP (Sigma-Aldrich, cat. no. C2920) and a mix of 0.5 µM rotenone (Sigma-Aldrich, cat. no. R8875) + 0.5 µM antimycin A (Sigma-Aldrich, cat. no. A8674)—separated by four measurement phases (baseline plus post‑injection 1–3). Each phase comprised three cycles of 3 min of mixing, 2 min of waiting and 3 min of measurement. After the assay, cells were lysed using M-PER Mammalian Protein Extraction Reagent (ThermoFisher, cat. no. 78501) supplemented with cOmplete Mini Protease Inhibitor Cocktail (Sigma-Aldrich, cat. no. 11836153001) and PhosSTOP (Sigma-Aldrich, cat. no. 4906845001), according to the manufacturer’s instructions. Total protein concentrations were determined using the Pierce Dilution-Free Rapid Gold BCA Protein Assay (ThermoFisher, cat. no. A55860), and oxygen‑consumption rate values were normalized to total protein content.

### CRISPR organoid assays

H1-hESC-iCas9 cells were transduced with a pooled gRNA library containing four gRNAs per target gene, with 20% of the library comprising nontargeting gRNAs. Following selection with 1 µg ml^−1^ puromycin, the established cell line was used to generate cortical organoids following a well-established protocol^121^ with slight modifications. In brief, embryoid bodies were generated using AggreWell plates (Stemcell Technologies) according to the manufacturer’s instructions. Once formed, embryoid bodies were transferred to ultralow-attachment 10-cm plates (Corning) for further culture. Patterning was initiated using StemFlex base media (A3349401, cat. no. Gibco) supplemented with 100 nM LDN-193189 (x) and 10 µM SB431542 (x). The medium was refreshed daily. Organoids were cultured on an orbital shaker at 53 rpm for the duration of the protocol. On day 6, the patterning medium was replaced with growth media: Neurobasal A medium (cat. no. 10888022, Gibco), 1 × GlutaMAX (cat. no. 35050061, Gibco) and 1 × B27 (cat. no. 12587010, Gibco), supplemented with 20 ng ml^−1^ FGF (PeproTech) and 20 ng ml^−1^ EGF (PeproTech). On day 14, Cas9 expression was induced by treating the organoids with 2 µg ml^−1^ doxycycline (Sigma-Aldrich) for 72 h. From day 25, FGF and EGF were replaced with 20 ng ml^−1^ BDNF (PeproTech) and 20 ng ml^−1^ NT-3 (PeproTech). Media changes were performed every other day. Starting from day 42, organoids were maintained in growth medium without additional supplements. Medium was refreshed 2–3 times per week.

The organoids were maintained in culture for ~80 d, at which point five organoids from three biological replicates were collected for DNA extraction using the DNeasy Blood & Tissue Kit (cat. no. 69504, Qiagen). Extracted DNA was subjected to PCR amplicon sequencing with UMIs using a three-step PCR protocol. In the first step (PCR-1), UMI-containing primers (5′-ACACTCTTTCCCTACACGACGCTCTTCCGATCTACGTGACGTAGAAAGTAATAATTTCTTGGGT-3′ and 5′-GTGACTGGAGTTCAGACGTGTGCTCTTCCGATCTN(25252525)NNNNNNNNNACTCGGTGCCACTTTTTCAA-3′) were used for four cycles. PCR-2 utilized adaptor primers (5′-ACACTCTTTCCCTACACGACGCTCTTCCGATCT-3′ and 5′-GTGACTGGAGTTCAGACGTGTGCTCTTCCGATCT-3′) for 22 cycles. PCR-3, performed by the sequencing facility, added sample-specific indexing in seven additional cycles. The prepared libraries were sequenced on a NovaSeq platform with paired-end 150-bp reads, generating 10 million reads per sample, at the YCGA.

Fragments amplified by PCR were sequenced on a NovaSeq 6000 sequencer paired-end at 150 bp with ~10 million reads per sample. Flanking sequences on both side of gRNAs were trimmed using BBDuk, and reads were then mapped to gRNA reference sequences and counted using the MAGeCK package^119^. Raw counts for each gRNA were normalized to counts of scrambled gRNA. Abundances of each gRNA-target gene were then calculated by sum of all gRNAs targeting that gene after excluding gRNAs with low KO efficiency (<5%). Average log_2_-transformed FCs of gRNA-target abundance were compared between doxycycline-induced versus uninduced samples in day 77 samples.

### Analysis of single-cell CRISPR-KO screens in NPCs, DIV7 and DIV21 iGLUTs, and DIV36 iGABAs

mRNA sequencing reads were mapped to the GRCh38 reference genome using the Cellranger Software (RRID:SCR_017344). To generate count matrices for GDO (gRNA) libraries, the kallisto indexing and tag extraction (kite) workflow was used. Count matrices were used as input into the R/Seurat package^122^ (v.5.1.0; RRID:SCR_007322) to perform downstream analyses, including quality control, normalization, cell clustering, GDO demultiplexing and covariate regression^29,123^. CRISPR-screen experiments in each cell type were processed independently. Within each cell type, ~100–80,000 cells were sequenced across four lanes. gRNA and RNA UMI feature counts were filtered removing the top and bottom deciles of cells based on distribution of counts in each cell type. The percentages of all the counts belonging to the mitochondrial, ribosomal and hemoglobin genes calculated using Seurat::PercentageFeatureSet were filtered with cell-type-specific thresholds, given the relatively high proportion of mitochondrial genes expressed in neurons. Mitochondrial, ribosomal and hemoglobin genes as well as MALAT1 were removed (^RP[SL][[:digit:]]|^RPLP[[:digit:]]|^RPSA|^HB[AEGQ][[:digit:]]|^HB[ABDMQ]|^MT-|^MALAT1$). Lowly expressed genes, those that had at fewer than two read counts in 90% of samples, were also removed. Hashtag and guide-tag raw counts were normalized using centered log ratio transformation, where counts were divided by the geometric mean of the corresponding tag across cells and log-transformed. gRNA demultiplexing was performed using the Seurat::MULTIseqDemux function for each lane individually and then counts were merged across lanes (Supplementary Fig. 3b). In NPCs, 94,363 cells were retained after filtering and removal of negatively assigned cells, with 62.7% classified as doublets and 37.3% classified as singlets. In DIV7 and DIV21 iGLUTs, 57,685 and 31,473 cells were retained, with 34% and 9.8% doublets and 66% and 90.2% singlets, respectively. In DIV35 iGABAs, 64,462 cells were retained, with 48.3% doublets and 51.7% singlets. For all downstream analyses, only cells with ‘singlet’ gRNA classification were used (26,549–38,097 cells per experiment) (Supplementary Fig. 4c–e). The number of singlet cells by gRNA per cell type shown in Supplementary Fig. 6a,b.

### Cell-type-specific population heterogeneity correction

Gene-expression-based clustering was largely driven by cellular heterogeneity, cell quality and sequencing lane effects. gRNA identity was not correlated with these covariates (Supplementary Fig. 7), so we adjusted for transcriptomic variability arising from cellular heterogeneity by applying maturity and cellular subtype scores across both perturbed and nonperturbed cells. First, variation related to cell-cycle phase of individual cells was accounted for by assigning cell-cycle scores using Seurat::CellCycleScoring, which uses a list of cell-cycle markers^124^ to segregate by markers of G2/M phase and markers of S phase. Second, to address variance due to cellular heterogeneity within a single experiment, we adapted the method applied by Seurat::CellCycleScoring to calculate a ‘Maturity.Score’ and ‘Subtype.Score’ for each cell based on cellular subtype (more variable in mature GABAergic neurons) and developmental time-point-specific markers (mora variable in NPCs and immature iGLUTs) (Supplementary Tables 2 and 3). Cells with outlier maturity scores and subtype scores were removed from downstream analyses. RNA UMI count data were then normalized and log-transformed, and the percentages of mitochondrial, hemoglobulin and ribosomal genes (markers of cell quality), lane, cell-cycle scores (Phase) and maturity scores regressed out using Seurat::SCTransform. The scaled residuals of this model represent a ‘corrected’ expression matrix, which was used for all downstream analyses.

Although demultiplexing assigned the correct guide identity to each cell, to remove ‘false positives’ whereby gRNAs were assigned but gene expression was unperturbed, the transcriptomes of gRNA clusters were evaluated relative to scramble gRNAs, ensuring that cells assigned to a guide-tag identity class demonstrated successful perturbation of the targeted NDD gene. To remove subsequent ‘false negatives’, whereby a successful CRISPR-KO may not result in significant downregulation of the targeted gene^29^ yet still achieve an overall transcriptomic profile distinct from scramble populations, we performed WNN analysis to assign clusters based on both guide-tag identity class and gene expression^30^. To identify successfully perturbed cells, the transcriptomes of gRNA clusters were compared with scramble-gRNA control clusters by differential gene expression analysis (Wilcoxon rank sum), comparing each cluster with all other clusters. Nontargeting WNN clusters and KO gRNA WNN clusters were filtered by setting a quantile base average expression threshold of target genes based on the distribution of target gene average expression across all other clusters. Clusters were the collapsed by gRNA identity; gRNAs with less than 75 cells were removed from analysis. These cells were then used for downstream differential gene-expression analyses^125^. For each cell type individually, single-cell gene expression matrices were Pseudobulked using scuttle::aggregateAcrossCells function across lanes (four pseudo-bulk samples per perturbation) and lowly expressed genes were removed (leaving 18,000–22,000 genes), followed by edgeR/limma differential gene expression analysis (RRID:SCR_010943). Concordance between Wilcoxon rank sum differential gene expression analysis using single-cell data and limma:voom using Pseudobulked data was assessed for each gene.

Altogether, Wilcoxon rank sum was applied to measure NDD gene knockdown from single-cell DEG analysis. Given the concordance between the DEG results using single-cell Wilcoxon and pseudo-bulk limma:voom (Supplementary Fig. 6c), all main figures and all Supplementary figures thereafter applied pseudobulked data analyzed with limma.

To validate whether the high correlation within-cell types was due to exactly the same scramble control cells, we re-performed DEG analyses using random selection of a subset of scramble cells for each cell type (Supplementary Fig. 8). Briefly, for each gene, 50% (if number of pseudobulked sample cells >50) or 80% (if number of pseudobulked sample cells <50) of scramble cells were randomly selected using the sample function from R. DEG analyses were then performed as described above using the limma/dreamlet package between KOs and a subset of scrambles different among genes. The process was repeated three times to avoid random selection bias and the median of each gene logFC was used as the final logFC. Average overlap of random scramble cells across different genes is ~50%.

### Meta-analysis of gene expression across perturbations^31^

Across NDD KOs, DEGs were meta-analyzed (METAL^126^), and ‘convergent’ genes were defined as those with significant and shared direction of effect across all NDD gene perturbations and with nonsignificant heterogeneity (FDR-adjusted *P*_meta_ < 0.05, Cochran’s heterogeneity *Q*-test *P*_Het_ > 0.05). To test convergence between NDD KOs, meta-analyses were performed across all possible combinations of 2–5 KO perturbations with and without sub-setting for those shared across cell types (>40,000 combinations across cell types) (Supplementary Data 1).

### Bayesian bi-clustering to identify convergent networks^31^

Across NDD KOs, convergent networks were generated by Bayesian bi-clustering^127^ and undirected gene co-expression network reconstruction from the NDD KOs. Not constrained by statistical cut-offs, and able to capture the effect of more lowly expressed genes, convergent networks may be a more sensitive measure of convergence. Networks were built based on bi-clustering (BicMix)^128^ using log_2_CPM (counts per million) expression data from all the replicates across each of the NDD gene sets and scramble gRNA jointly. We performed 40 runs of BicMix with the default parameters on these data and the output from iteration 400 of the variational Expectation-Maximization algorithm was used. Target Specific Network reconstruction^128^ was performed to identify convergent networks across all possible combinations of the nine NDD gene KO perturbations shared across cell types (*n* = 502 combinations per cell type) and randomly sampled combinations of 2–21 KO perturbations without sub-setting for those shared across cell types (*n* = 1,400–2,300 combinations).

### Influence of functional similarity on convergence degree

To test the influence of functional similarity and brain co-expression between KOs on convergence and compare the degree of convergence between the same KOs in different cell types, we established two methods for defining and measuring convergence. First, gene-level convergence using meta-analysis as described above, with the strength of convergence for each set defined as ratio of convergent genes to the average number of DEGs.

$$\mathrm{Gene}\,\mathrm{level}\,\mathrm{convergence}=\frac{n\mathrm{Convergent}\,\mathrm{genes}}{\mathrm{Mean}(\displaystyle {\sum }_{1}^{N}n\mathrm{DEGs})}$$

Second, network-level convergence based on undirected network reconstruction from Bayesian bi-clustering as described above. Bi-clustering identifies co-expressed genes shared across the downstream transcriptomic impacts of any given set of KO perturbations; thus, the resolved networks are the transcriptomic similarities between distinct perturbations (convergence). We calculated the ‘degree of convergence’ for each network based on a previously described metric^31^. Briefly, convergence scores are based on: (1) network connectivity, as defined by the sum of the clustering coefficient (Cp) and the difference in average length path (Lp) from the maximum average length path resolved across all possible sets [(max)Lp-Lp]; (2) similarity of network genes based on biological pathway membership scored by taking the sum of the mean semantic similarity (semsim) scores^129^ between all genes in the network; and (3) minimum percentage duplication rate across 40 runs. Duplication thresholds are network-dependent and a metric of confidence in the connections.

$$\begin{array}{l}\mathrm{Network}\,\mathrm{level}\,\mathrm{convergence}=\,\mathrm{Cp}+[(\mathrm{Lp})\,-\mathrm{Lp}]\\ +\mathrm{mean}\left(\mathop{\sum }\limits_{1}^{N}\mathrm{MF}\,\mathrm{semsim}+\mathrm{BP}\,\mathrm{semsim}+\mathrm{CC}\,\mathrm{semsim}\right)\\ +n\mathrm{Duplication}\,\mathrm{runs}/n\mathrm{Total}\,\mathrm{runs}\end{array}$$

Functionally, similarity scores across the NDD KO genes represented in each set were calculated using: (1) Gene Ontology Semantic Similarity Scores: the average semantic similarity score based on Gene Ontology pathway membership within Biological Pathway (BP), Cellular Component (CC) and Molecular Function (MF) between NDD genes in a set^129^; and (2) brain expression correlation score: based on the strength of the correlation in NDD gene expression in the Common Mind Consortium (CMC) (*n* = 991 after quality control) post-mortem DLPFC gene expression data.

We performed Pearson’s correlation analysis (Holm’s-adjusted *P*) on similarity scores and the degree of network convergence to determine the influence of the similarity of the initial KO genes on downstream convergence. We compared the average strength of convergence across cell types using a parametric Welch’s *F*-test and pairwise Games–Howell test.

### Enrichment analysis of convergence for risk loci using MAGMA

We intersected cross-cell-type perturbation-specific and cross-perturbation cell-type-specific gene-level convergence with genetic risk of psychiatric and neurological disorders/traits (attention-deficit/hyperactivity disorder^130^, anorexia nervosa^131^, ASD^2^, alcohol dependence^132^, bipolar disorder^133^, cannabis use disorder^134^, major depressive disorder^135^, obsessive-compulsive disorder^136^, post-traumatic stress disorder^137^, SCZ^138^, Cross disorder^139^, Alzheimer disease^140^, Parkinson disease^141^, amyotrophic lateral sclerosis^142^, Tourette’s^143^, migraine^144^, chronic pain^145^ and neurotic personality traits^146^, GWAS summary statistics) using multi-marker analysis of genomic annotation (MAGMA)^23^. SNPs were mapped to genes based on the corresponding build files for each GWAS summary dataset using the default method, snp-wise = mean (a test of the mean SNP association). A competitive gene set analysis was then used to test enrichment in genetic risk for a disorder across gene sets with an FDR < 0.05.

To test whether observed effects were due to the differential size of the gene sets for each GWAS or owing to the fact that DEGs are more likely to include neural genes, which are more likely to be associated with brain disorder, GWAS sets were filtered for genes expressed in each cell type before enrichment testing, and enrichment tests were performed after randomly down-sampling GWAS gene sets to 100, 250, 500, 750 and 1,000 genes (Supplementary Fig. 9), and performed ten times within each set size (that is, 50 tests for each GWAS).

### Over-representation analysis, functional enrichment annotation and biological theme comparison of convergence

To identify pathway enrichments unique to individual KOs, convergent genes and convergent networks based on zebrafish behavioral subgroups (see the zebrafish methods below), we performed biological theme comparison and gene set enrichment analysis (GSEA) (ClusterProfiler^147^; RRID:SCR_016884). Using FUMAGWAS: GENE2FUNC, the 102 ASD genes were functionally annotated and an over-representation gene-set analysis for each convergent gene set was performed^148^. Using WebGestalt (WEB-based GEne SeT AnaLysis Toolkit^149^; RRID:SCR_006786), over-representation analysis was performed on all convergent gene sets against publicly available gene set lists GeneOntology, KEGG, DisGenNet and Human Phenotype Ontology, and a curated gene list of rare-variant targets associated with ASD, SCZ and ID^25^.

### Random forest prediction model of convergence strength

To determine how well functional similarity between KOs can predict gene-level and network-level convergence, we trained a random forest model^32^ (randomForest package in R; RRID:SCR_015718) for each type of convergence, evaluated the model in an independent internal dataset and validated the model in an external CRISPRa activation screen^31^. Data from randomly tested gene combinations (2–5 KO sets at the gene level and 2–10 KO sets at the network level) tested across cell types were randomly down-sampled into a training set (70%) and a testing set (30%)—all with comparable proportions of data by cell type. The random forest model was trained with bootstrap aggregation using CC, MF and BP semantic similarity scores, brain expression correlation, number of genes and cell type as predictors. The random forest linear regression model was evaluated in the testing data by comparing actual values with predicted values, estimating the root mean squared error and performing Pearson’s correlations. Predictor models were validated using an external dataset of ten CRISPR-activation perturbations of SCZ common variant target genes with multifunctional annotations broadly grouped as signaling/cell communication (*CALN1*, *NAGA*, *FES*, *CLCN3*, *PLCL1*) and epigenetic/regulatory (*SF3B1*, *TMEM219*, *UBE2Q2L*, *ZNF804A*, *ZNF823*)^31^, and assessed based the root mean squared error and Pearson’s correlation between actual and predicted convergence strength.

### LNCTP in silico model

To investigate the perturbation of ASD genes in silico, we adapted the LNCTP model^33^ to predict the effects of changes in gene expression in the PFC, across neuronal and non-neuronal cell types. The LNCTP is defined as an energy model representing the joint distribution of a collection of phenotypes of interest conditioned on the genotype. Since we are interested primarily in the effects of gene expression perturbations on the expression of other genes, we use only the imputation segment of the LNCTP model (excluding the prediction of higher-order phenotypes and cell–cell interactions).

The probabilistic model for the imputation-based LNCTP may be expressed as:

1 $$\begin{array}{ll}{P}_{\mathrm{LNCTP}}({x}_{i},|,{z}_{i}) & =\exp (-E({x}_{i},|,{z}_{i}))E({x}_{i},|,{z}_{i})\\ & ={x}_{i0\cdot }^{T}J{x}_{i0\cdot }+\mathop{\sum }\limits_{g}{x}_{i0g}^{T}b({z}_{i},{\beta }_{g})+\mathop{\sum }\limits_{c}({x}_{ic\cdot }^{T}{J}_{c}{x}_{ic\cdot }+{x}_{ic\cdot }^{T}{b}_{c})\\ & +\lambda \mathop{\sum }\limits_{g}{({x}_{i0g}-f{({z}_{i})}^{T}{x}_{i,1\ldots C,g})}^{2}\end{array}$$

Here ${z}_{i}$ represents the genotype of individual *i*, and ${x}_{i}$ represents bulk and cell-type-specific gene expression from individual *i*. We further index the gene expression by $C$ cell types (which are here: excitatory neurons, inhibitory neurons, oligodendrocytes, astrocytes, oligodendrocyte precursor cells, endothelial cells and microglia), which will be denoted ${x}_{1},\,{x}_{2},\,\ldots {x}_{C}$, and we will use ${x}_{0}$ to denote the bulk expression. The variables ${f}_{1\ldots C}$ represent the estimated cell fractions in the bulk observations (predicted from the genotype, $z$). The parameters of the model are $\theta =\{{\beta }_{1\ldots G},\,{J}_{0\ldots C}\}$ and $\lambda$ acts as a hyperparameter. The parameters ${\beta }_{1\ldots G}$ and ${J}_{0\ldots C}$ reflect the gene-specific expression biases and pairwise interactions, respectively, whose nonzero elements are determined by the sparsity structure arising from expression quantitative trait loci (eQTLs) and gene regulatory network (GRN) linkages, respectively; the nonzero elements of ${J}_{c}$ occur only between genes connected in the GRN of cell type $c$.

Further details on the training of the model in Eq. (1) can be found in ref. ^33^; here, we outline the specific differences in the training for the purposes of our analysis. As in ref. ^33^, we used genetics and expression data from post-mortem PFC samples from the PsychENCODE consortium. However, we group together samples from all higher-order phenotypes during training (control, SCZ, bipolar disorder and ASD), and split the data into three partitions of size 760, 100 and 100 for training, validation and testing, respectively (each including samples from all higher-order phenotypes). Further, we include all 29 CRISPR targeted genes, 102 NDD genes^5^, transcription factors^33^ and neuropsychiatric transcriptome-wide association analysis (TWAS)-selected genes^33^, and the top 100 up- and downregulated CRISPR convergent genes in iGLUT and iGABA cells (400 genes in total), in the model, generating 1,325 genes in total. The expression quantitative trait loci (eQTL) and GRN linkages from PsychENCODE are then restricted to this subset of genes.

### LNCTP simulating perturbations

To perform perturbations in this model corresponding to the 29 CRISPR targeted genes, we use the following perturbation-conditioned version of the LNCTP model:

2 $$\begin{array}{l}{P}_{\mathrm{LNCTP}}({x}_{i,{\rm{\neg }}({c}^{\ast },{g}^{\ast })},|,{z}_{i},{x}_{i,{c}^{\ast },{g}^{\ast }}=\{k,-k\})\\ =\exp ({x}_{i,{\rm{\neg }}({c}^{\ast },{g}^{\ast })},|,{z}_{i},{x}_{i,{c}^{\ast },{g}^{\ast }}=\{k,-k\})\\ E({x}_{i,{\rm{\neg }}({c}^{\ast },{g}^{\ast })},|,{z}_{i},{x}_{i,{c}^{\ast },{g}^{\ast }}=\{k,-k\})={x}_{i0\cdot }^{T}J{x}_{i0\cdot }\\ +\mathop{\sum }\limits_{g}{x}_{i0g}^{T}b({z}_{i},{\beta }_{g})+\mathop{\sum }\limits_{c}({x}_{ic\cdot }^{T}{J}_{c}{x}_{ic\cdot }+{x}_{ic\cdot }^{T}{b}_{c})\\ +\lambda \mathop{\sum }\limits_{g}{({x}_{i0g}-f{({z}_{i})}^{T}{x}_{i,1\ldots C,g})}^{2}+K\delta ({x}_{i,{c}^{\ast },{g}^{\ast }}=\{k,-k\})\end{array}$$

where $({c}^{* },\,{g}^{* })$ denotes the perturbed gene and cell type, whose expression is set to $k$ or $-k$, $\delta (a)$ is a delta function whose value is 0 if expression $a$ is true, and 1 otherwise, and $K$ is an arbitrarily large value. We perturb each of the CRISPR targeted genes in turn in the bulk network, using $k=2$, and applying a negative perturbation to mimic the effect of the CRISPR perturbation. We note that, since the model is trained on *Z*-scored log-normalized expression counts, this corresponds to introducing a large negative FC to the selected gene. The in silico-predicted log FCs per individual across all genes (per cell type) are then calculated by comparing the expected values before and after perturbation:

3 $${\Delta }_{i,c,g}={{\mathbb{E}}}_{{P}_{{\rm{LNCTP}}}(.,|,{z}_{i})}[{x}_{i,c,g}]-{{\mathbb{E}}}_{{P}_{{\rm{LNCTP}}}(.,|,{z}_{i},{x}_{i,{c}^{\ast },{g}^{\ast }}=\{k,-k\})}[{x}_{i,c,g}]$$

and the final predicted log FCs are calculated by taking the expectation across individuals. We use the sampling approach in ref. ^33^ to evaluate the expectations in Eq. (3).

To perform perturbations across all 102 NDD genes, for efficiency, we learn a reduced model by removing the dependency on ${z}_{i}$ in Eq. (1). We sample cell-type-specific expression values for each individual from the full model, and then fit the reduced model by refitting the model parameters to maximize the likelihood of the full data vectors (consisting of the original bulk and sampled cell-specific expression vectors for each individual). Perturbations are performed in the reduced model as in Eq. (2) and FCs are calculated as in Eq. (3), while removing the dependency on ${z}_{i}$ and the *i* subscripts, respectively.

### LNCTP in silico convergent genes

To identify in silico convergent genes for a set of perturbations, $S=\left\{\left({c}_{1}^{* },{g}_{1}^{* }\right),\ldots ,\left({c}_{N}^{* },{g}_{N}^{* }\right)\right\}$, we calculate ${\Delta }_{c,g}$ using Eq. (3) for each perturbation, writing ${\Delta }_{c,g}^{{c}^{* },{g}^{* }}$ for the log FC to $\left(c,g\right)$ generated by applying perturbation $\left({c}^{* },{g}^{* }\right)$, and ${\Delta }_{c,g}^{S}$ for the vector of log FCs created by applying all perturbations in $S$. Then, the set of in silico convergent genes for $S$ is found by selecting those for which ${P}_{{\rm{sign}}}({\Delta }_{c,g}^{S}[|{\Delta }_{c,g}^{S}|\ge \tau ]) < 0.1$, where ${P}_{{\rm{sign}}}(.)$ is the *P* value from a two-tailed one-sample sign-test, and juxtaposition represents element-wise multiplication of vectors. The threshold $\tau$ is introduced to reduce noise from perturbations that are estimated to generate small log FCs, and throughout we set $\tau =0.3$.

For the comparison of in silico convergent genes derived from different perturbation sets $S$, we apply two-sided hypergeometric tests to the gene sets defined as above (using all 1,325 genes in our model as the background set). For GSEA of convergent genes derived from $S$, we apply clusterprofiler^147^ to the full set of genes in our model, ranked by ${P}_{{\rm{sign}}}({\Delta }_{c,g}^{S}[|{\Delta }_{c,g}^{S}|\ge \tau ])$ as defined above.

### LNCTP semantic distance test

To test the semantic distance between enriched terms for two sets of perturbations, ${S}_{1}$ and ${S}_{2}$, we generate the set of enriched terms ${T}_{1}$ and ${T}_{2}$ by applying GSEA to each set as described above (using Benjamini–Höchberg correction and an FDR threshold of 0.2 to select enriched terms ${T}_{1}$ and ${T}_{2}$). We then calculate the similarity between terms ${t}_{1}$ and ${t}_{2}$ by evaluating $s\left({t}_{1},{t}_{2}\right)=\left|G\left({t}_{1}\right)\cap G\left({t}_{2}\right)\right|/\left|G\left({t}_{1}\right)\cup G\left({t}_{2}\right)\right|$, where $G\left(t\right)$ denotes the set of genes occurring in the leading edge of term $t$. We test for a significant semantic distance between ${S}_{1}$ and ${S}_{2}$ by evaluating $s\left({t}_{1},{t}_{2}\right)$ between all pairs ${t}_{1}\in {S}_{1}$, ${t}_{2}\in {S}_{2}$, versus all pairs ${t}_{1}\in {S}_{1}$, ${t}_{2}\in {S}_{1}$ and ${t}_{1}\in {S}_{2}$, ${t}_{2}\in {S}_{2}$, and applying a one-sided rank-sum test for the smaller similarity in the former pairs versus the latter.

### Transcriptional correlations between hiPSC-derived neural cells, fetal and adult brain cell types and the zebrafish brain

We compared wild-type (WT) zebrafish brain expression with gene expression in our hiPSC-derived models and with sign-cell expression data for the fetal and adult PFC (PsychENCODE^150,151^: http://resource.psychencode.org/Datasets/Derived/SC_Decomp/DER-20_Single_cell_expression_processed_TPM.tsv). We first filtered zebrafish gene names and converted them to the appropriate *Homo sapiens* orthologs using the R package orthogene (v3.2.1^152^); genes without matched orthologs were dropped from both species. Pseudo-bulk expression data from scramble control cells were used as the baseline expression across NPCs, day 7 iGLUTs, day 21 iGLUTs and day 36 iGABAs. Pearson’s correlation coefficients between in vitro cells, fetal and adult post-mortem brain cells and zebrafish brain were calculated and a Bonferroni correction applied.

### Zebrafish

All procedures involving zebrafish were conducted in accordance with IACUC (protocol no. 2024-20054) regulatory standards at Yale University. Zebrafish larvae were raised at 28 °C on a 14-h:10-h light:dark cycle. Larvae were grown in 150-mm Petri dishes in blue water (0.3 g l^−1^ Instant Ocean, 1 mg l^−1^ methylene blue, pH 7.0) at a density of 60–80 larvae per dish. Behavioral assays were conducted in zebrafish larvae at 5–7 days post fertilization (dpf). At these developmental stages, sex is not yet determined.

### Zebrafish mutant generation

We performed automated, high-throughput, quantitative behavioral profiling of larval zebrafish to measure arousal and sensorimotor processing as a read-out of circuit-level deficits resulting from gene perturbation^41^. We quantified 24 parameters across sleep–wake activity and visual-startle responses in 18 stable homozygous mutant or F0 mosaic crispant lines for 15 NDD genes (Supplementary Tables 7 and 8). Stable zebrafish lines were generated by our lab (*arid1b*^*Δ7/Δ7*^, *chd2*^*Δ7/Δ7*^, *chd8*^*Δ7/Δ7*^, *chd8*^*Δ5/Δ5*^, *kdm5ba*^*Δ17/Δ17*^*b*^*Δ14/Δ14*^, *kdm5ba*^*4i/4i*^*b*^*Δ4/Δ4*^)^41^ or provided as a generous gift from the Thyme lab (*ash1l*^*1i,Δ60,19i/1i,Δ60,19i*^, *kmt5b*^*Δ208,1i,Δ5/Δ208,1i,Δ5*^, kmt2ca^*Δ82,17i/Δ82,17i*^*b*^*Δ6,Δ29/Δ6,Δ29*^, *nrxn1a*^*Δ218/Δ218*^)^153,154^.

kmt5b uab320/+: ZDB-ALT-220315-26

kmt2ca uab318/+: ZDB-ALT-220315-24

kmt2cb uab319/+: ZDB-ALT-220315-25

ash1l uab305/+: ZDB-ALT-220315-11

chd8 ya509/+: ZDB-ALT-240514-1

chd8 ya510/+: ZDB-ALT-240514-2

kdm5ba ya523/+: ZDB-ALT-240514-15

kdm5bb ya525/+: ZDB-ALT-240514-17

kdm5ba ya524/+: ZDB-ALT-240514-16

kdm5bb ya 526/+: ZDB-ALT-240515-1

nrxn1a uab403/+: ZDB-ALT-220720-20

F0 crispants for the following genes were generated as in ref. ^155^: *chd2*, *kdm6bab*, *mbd5*, *phf12ab*, *phf21aab*, *skiab*, *smarcc2*, *wacab*. Briefly, we designed two CRISPR crRNAs per allele, prioritizing early exons for targeting. CRISPR RNPs were assembled individually and then combined before injection at the one-cell stage. The number of scrambled guides injected into the control group was matched to the number of CRISPR guides used for the experimental group. Injected embryos were raised to 5 dpf at which point the behavioral assays (described below) were conducted. We identified unique behavioral fingerprints for each NDD gene mutant, revealing convergent and divergent phenotypes across mutants (Supplementary Fig. 22b). To classify convergent behavioral subgroups that may share circuit-level functions, we performed correlation analyses with hierarchical clustering across mutants. We identified four distinct subgroups of NDD genes with highly correlated behavioral features (Fig. 7a).

### Behavioral assays

Larvae were placed into individual wells of a 96-well plate (cat. no. 7701-1651; Whatman) containing 650 μl of standard embryo water (0.3 g l^−1^ Instant Ocean, 1 mg l^−1^ methylene blue, pH 7.0) per well within a Zebrabox (ViewPoint Life Sciences). Locomotion was quantified with an automated video-tracking system (Zebrabox and ZebraLab software). The visual-startle assay (VSR) was conducted at 5 dpf (ref. ^41^). Briefly, to assess larval responses to lights-off stimuli (VSR-OFF), larvae were acclimated to white light for 1 h, and baseline activity was tracked for 30 min followed by five 1-s dark flashes with intermittent white light for 29 s. To evaluate larval responsivity to lights-on stimuli (VSR-ON), the assay was reversed, where larvae were acclimated to darkness for 1 h, and baseline activity was tracked for 30 min followed by five 1-s white light flashes with intermittent darkness for 29 s. For VSR-OFF and VSR-ON, six behavioral parameters were quantified using custom MATLAB code^41^ (available from GitHub via https://github.com/ehoffmanlab/Weinschutz-Mendes-et-al-2023-behavior; 10.5281/zenodo.7644898(ref. ^156^)): (1) average intensity of all startle responses; (2) average post-stimulus activity; (3) average activity after first stimulus; (4) stimulus versus post-stimulus activity; (5) intensity of responses to the first stimulus; (6) intensity of responses to the final stimulus. To assess arousal responses in daytime and nighttime, the sleep–wake paradigm was conducted between 5 and 7 dpf, following the VSR-OFF and VSR-ON assays. During a 14-h:10-h white light:darkness cycle, larvae activity and sleep patterns were tracked within the Zebrabox and analyzed with custom MATLAB code^41,157,158^ (available from GitHub via (https://github.com/JRihel/Sleep-Analysis/tree/Sleep-Analysis-Code; 10.5281/zenodo.7644073 (ref. ^159^)). Six behavioral parameters were quantified for daytime and nighttime: (1) total activity; (2) total sleep; (3) waking activity; (4) rest bouts; (5) sleep length; (6) sleep latency. Across VSR-OFF, VSR-ON and sleep–wake assays, we analyzed 24 parameters. For stable mutant lines, behavioral assays were performed blind to genotype using sibling- or cousin-matched larvae from heterozygous in-crosses with genotyping performed after each experiment. Experiments were repeated at least in duplicate for each line. Our analysis focused on homozygous mutants, which typically exhibit more robust phenotypes than heterozygotes. For CRISPR F0 mutants, experiments were performed once with experimenters aware of which larvae were injected with scrambled versus gene-targeting RNPs. Larval movement was quantified blind to genotype using commercial tracking software as described above. Larvae were genotyped after each experiment to confirm the presence of on-target mutations. Baseline behavioral screening sample sizes are as follows: *kmt5b* WT *n* = 62, HOM *n* = 71; *mbd5* scrambled *n* = 39, crispant *n* = 56; *kdm5bab* 4i:del4 WT *n* = 42, HOM *n* = 55; *kdm5bab* del17:del14 *n* = 68, HOM *n* = 69; *phf12ab* scrambled *n* = 32, crispant *n* = 15; *skiab* scrambled *n* = 45, crispant *n* = 33; *chd2* scrambled *n* = 43, crispant *n* = 40; *smarcc2* scrambled *n* = 36, crispant *n* = 35; *kdm6bab* scrambled *n* = 40, crispant *n* = 34; *kmt2cab* WT *n* = 38, HOM *n* = 20; *wacab* scrambled *n* = 32, crispant *n* = 32; *arid1b* WT *n* = 81, HOM *n* = 81; *phf21a* scrambled *n* = 52, crispant *n* = 44; *chd8* del7 WT *n* = 180, HOM *n* = 164; *chd8* del5 WT *n* = 143, HOM *n* = 167; *ash1l* WT *n* = 19, HOM *n* = 23; *nrxn1a* WT *n* = 24 HOM *n* = 28. Based on our power analyses for baseline behavioral experiments, we estimated that a sample size of 28–41 fish per group provides 80% power to detect a 20–30% change in behavior (ANOVA, α set at 0.05).

### Behavioral analysis

Linear mixed models (LMMs) were used to compare phenotypes of each behavioral parameter between homozygous mutant versus WT or crispant versus scramble-injected fish for each gene of interest. Variations of behavioral phenotypes across experiments were accounted for by including the date of the experiment as a random effect in LMM. Hierarchical clustering analysis was performed to cluster mutants and behavioral parameters based on signed −log_10_-transformed *P* values from LMM, where sign indicates direction of the difference in behavioral phenotype when comparing stable mutant with WT or crispant with scrambled-injected. Pearson’s correlation analysis was used to assess correlations between mutants based on the difference in the 24 parameters. Difference was evaluated using signed −log_10_-transformed *P* values. For the behavioral data, we assumed the data distribution to be normal but not of equal variance. We did not formally test the normality assumption.

### Drug prioritization based on zebrafish pharmaco–behavioral profiles

NDD gene-associated mutant and crispant behavioral phenotypes were compared with a dataset of 376 US Food and Drug Administration-approved drugs that were screened for their behavioral effects in larval zebrafish using the visual-startle and sleep–wake assays described above^46^. These drugs have a significant effect on at least two behavioral parameters (LMM, *P* < 0.05/3, corrected for three behavioral assays). Pearson’s correlation analysis was used to identify drugs that significantly correlate (correlation > 0.5, *P* < 0.05, *t*-statistic) or anti-correlate (correlation < −0.5, *P* < 0.05, *t*-statistic) with mutant behavioral signatures (Supplementary Data 2 and 3).

### Drug prioritization based on perturbation signature reversal in LiNCs neuronal cell lines

To identify drugs that could reverse cell-type-specific convergence across different KOs, we used the Query tool from The Broad Institute’s CMap Server^45^. Briefly, the tool computes weighted enrichment scores (WTCS) between the query set and each signature in the CMap LiNCs gene expression data (dose, time, drug, cell line), normalizes the WTCS by dividing by the signed mean within each perturbation (NCS) and computes FDR as fraction of ‘null signatures’ (DMSO) where the absolute NCS exceeds reference signature. We prioritized drugs that were negatively enriched for convergent signatures specifically in neuronal cells (either neurons or NPCs with NCS ≤ −1.00, FDR ≤ 0.05) and filtered for drugs that had clinical data in humans and paired behavioral phenotyping in zebrafish (Supplementary Data 2).

### Targeted drug rescue of behavioral phenotypes in zebrafish

For mutant × drug experiments, larval activity was monitored from 5–7 dpf using the behavioral assays described above. Individual WT zebrafish larvae were added to each well of a 96-well plate containing 650 μl of standard embryo water. A 5 mM stock solution of each compound dissolved in DMSO or DMSO alone (control) was pipetted directly into each well after which the visual-startle and sleep–wake assays were performed. Drugs were tested at a final concentration of 10 μM (0.2% DMSO final concentration) in background-matched homozygous and WT larvae or crispant and scrambled control-injected larvae. For stable mutant lines, behavioral assays were performed blind to genotype using sibling- or cousin-matched larvae from heterozygous in-crosses with genotyping performed after each experiment. For CRISPR F0 mutants, experiments were performed once with experimenters aware of which larvae were injected with scrambled versus gene-targeting RNPs. Larval movement was quantified blind to genotype using commercial tracking software as described above. Larvae were genotyped after each experiment to confirm the presence of on-target mutations. Mutant × drug behavioral assays were repeated 1–3× for stable mutant lines and 1× for crispants. Sample sizes are as follows: *phf21a* + amiodarone scrambled DMSO *n* = 24, F0 DMSO *n* = 22, F0 AMIO *n* = 23; *phf21a* + fluvoxamine scrambled DMSO *n* = 24, F0 DMSO *n* = 22, F0 FLUVO *n* = 21; *chd2* + pravastatin WT DMSO *n* = 27, HOM DMSO *n* = 21, HOM PRAVA *n* = 23; *kdm6b* + paclitaxel scrambled DMSO *n* = 24, F0 DMSO *n* = 24, F0 PACLI *n* = 24; *kdm6b* + sirolimus scrambled DMSO *n* = 24, F0 DMSO *n* = 24, F0 SIRO *n* = 23; *kmt5b* + paclitaxel WT DMSO *n* = 20, HOM DMSO *n* = 22, HOM PACLI *n* = 26; *kmt5b* + sirolimus WT DMSO *n* = 27, HOM DMSO *n* = 26, HOM SIRO *n* = 20; *ash1l* + sunitinib WT DMSO *n* = 14, HOM DMSO *n* = 13, HOM SUN *n* = 12; *ash1l* + ezetimibe WT DMSO *n* = 15, HOM DMSO *n* = 12, HOM EZE *n* = 9; *ash1l* + rosuvastatin WT DMSO *n* = 27, HOM DMSO *n* = 22, HOM ROSU *n* = 22; *ash1l* + repaglinide WT DMSO *n* = 11, HOM DMSO *n* = 16, HOM REPAG *n* = 14. Based on our power analysis, we estimate that a sample size of 10–12 fish per group will provide 95% power to detect a 40–43% change in behavior (ANOVA, α set at 0.05), based on our previous pharmaco-behavioral screen^160^.

For behaviors that were nominally significantly different between mutant + DMSO and WT + DMSO (*P* < 0.06), we characterized the effect of the mutant × drug on behavior as: (1) ‘exacerbated’ (significant effect mutant + drug-v-WT > significant effect mutant-v-WT) if mutant behavior *P* ≤ 0.06 and mutant × drug behavior *P* value ≤ mutant behavior *P* value with increased absolute beta values (that is, stronger *P* value with appreciable difference in the magnitude of effect but not direction); (2) ‘unchanged’ (significant effect mutant + drug-v-WT = significant effect mutant-v-WT); (3) ‘partial rescue’ (significant effect mutant + drug-v-WT < significant effect mutant-v-WT) if mutant behavior *P* value ≤ mutant × drug behavior *P* value with reduced effects on the absolute beta value; (4) ‘rescued’ (significant effect mutant-v-WT, no significant effect mutant + Drug-v-WT) if mutant behavior *P* ≤ 0.06 and mutant × drug behavior *P* > 0.06; (5) ‘over-corrected’ (mutant + drug-v-WT opposite direction of significant effect mutant-v-WT) if mutant behavior *P* ≤ 0.06 and mutant × drug behavior *P* ≤ 0.06, with opposing directions of effect.

### Reporting summary

Further information on research design is available in the Nature Portfolio Reporting Summary linked to this article.

## Online content

Any methods, additional references, Nature Portfolio reporting summaries, source data, extended data, supplementary information, acknowledgements, peer review information; details of author contributions and competing interests; and statements of data and code availability are available at 10.1038/s41593-026-02247-7.

## Supplementary information

Supplementary Information Supplementary Figs. 1–24.

Reporting Summary

Supplementary Tables 1–9 Supplementary Tables 1–9.

Supplementary Data 1 Cell-type-specific convergent gene lists based on NDD pooled scCRISPR-KO.

Supplementary Data 2 Convergent sets and drug predictions based on mutant zebrafish behavioral clusters.

Supplementary Data 3 Gene × drug mutant zebrafish behavioral effects.

Supplementary Data 4 Key resource table.

## Source data

Source Data Statistical source data for Figs. 1–7.

## Data Availability

Key Resource Table available at 10.5281/zenodo.19457279. All hiPSCs are available from the Rutgers University Cell and DNA Repository (study 160; http://www.nimhstemcells.org/). Protocols are available from https://www.protocols.io/workspaces/brennand-laboratory. scRNA-seq data reported in this paper are publicly available from the Gene Expression Omnibus (GSE319096). Previously published SCZ CRISPRa screen datasets that were used for external validation of random forest models are available from the GEO (GSE200774) and from Synapse (syn27819129). Secondary summary statistics from the main analysis are available from Synapse (syn72039767). Source data are provided with this paper.

## Code availability

The full analysis pipeline (including code and processed data objects) used for analysis of single-cell CRISPR-KO data, evaluation and characterization of gene-level and network-level convergence, and predictive modeling using random forests are publicly available from Synpase (syn72039767). Custom MATLAB software developed by the Hoffman Lab to analyze visual-startle response parameters is available from GitHub via https://github.com/ehoffmanlab/Weinschutz-Mendes-et-al-2023-behavior; 10.5281/zenodo.7644898 (ref. ^156^). Custom MATLAB software developed by J. Rihel to analyze sleep–wake assays is available from GitHub via https://github.com/JRihel/Sleep-Analysis/tree/Sleep-Analysis-Code; 10.5281/zenodo.7644073 (ref. ^159^).
