# Supplementary material for: Transcriptomic and phenotypic convergence of neurodevelopmental disorder risk genes in vitro and in vivo
Source: Nat Neurosci. 2026 Apr 24;29(5):1079–94. doi: 10.1038/s41593-026-02247-7 (PMC13156037; doi:10.1038/s41593-026-02247-7)
Supplement: Supplementary file 1 — Supplementary Figs. 1–24. [file 41593_2026_2247_MOESM1_ESM.pdf]

nature neuroscience

Supplementary information

https://doi.org/10.1038/s41593-026-02247-7

Transcriptomic and phenotypic convergence of neurodevelopmental disorder risk genes in vitro and in vivo

In the format provided by the authors and unedited

Inventory of Supplemental Material

Supplemental Tables

SI Table 1. gRNA sequences for each NDD target gene.

SI Table 2. Cell maturity and subtype markers used to correct for cellular heterogenity and remove subtype clusters in ECCITEseq.

SI Table 3. Survival scores for ASD target Genes for Tian et al. 2020 compared with the number of successfully perturbed cells and average abundance of gRNAs by MiSeq.

SI Table 4. Disorder and behavioral associations of top convergent up and down-regulated genes by cell-type from MalaCards, OMIM, & GWAS catalogue.

SI Table 5. Disorder and behavioral associations of top nodes by cell-type

SI Table 6. Convergent nodes that overlap with CNV and rare variant target genes for each cell-type

SI Table 7. Generation of stable mutant zebrafish lines.

SI Table 8. Design and generation of F0 mutant zebrafish lines.

SI Table 9. Disorder and behavioral associations of top nodes by cell-type and behavioral set from MalaCards, OMIM, and GWAS catalogue**.**

Supplemental Figures

SI Figure 1. Characterization of 29 NDD rare variant target genes.

SI Figure 2. Characterization of gene expression of 29 NDD rare variant target genes in hiPSC neurons and the developing brain.

SI Figure 3. NDD pooled scCRISPR-KO screen validation.

SI Figure 4. NDD scCRISPR-KO screen in hiPSC derived iNPCs, iGLUTs, and iGABAs.

SI Figure 5. Validation of CRISPR-induced genome editing on selected genes with bulk RNAseq.

SI Figure 6. Characterization of perturbed cells and concordance between single-cell and pseudobulk differential gene expression analysis.

SI Figure 7. Degree of down-regulation of KO expression does not significantly correlate with downstream transcriptomic impacts.

SI Figure 8. Stronger correlations between individual KOs in mature neurons are replicated after random sub-setting of controls.

SI Figure 9. GWAS enrichments for individual KO effects across cell-types.

SI Figure 10. NDDs show little transcriptomic convergence across cell-types and maturity.

SI Figure 11. Gene-level convergence across 9 NDDs is unique across cell-types and increases with maturity in iGLUT neurons.

SI Figure 12. Gene-level convergence across 9 NDDs is enriched for pathways involved in neural development, mitochondrial function, and translational regulation.

SI Figure 13. Gene-level and network-level convergence across 21 NDDs in iGLUT and iGABA neurons.

SI Figure. 14. Schema of random forest training model and external validation.

SI Figure 15. Random forest training data characteristics and node frequency across trees.

SI Figure. 16. NDD risk genes with strong ASD associations resolve greater convergence across cell-types.

SI Figure. 17. Analysis of LNCTP convergent genes.

SI Figure. 18. Impact of NDD KO on proliferation and neurogenesis in NPCs and neurons.

SI Figure. 19. Pooled CRISPR screen to uncover the NDD KO effects on cortical organoid development.

SI Figure 20. High resolution microscopy captures diversity of mitochondrial morphology in iGLUTs and is sensitive to mitochondrial pharmacological insults.

SI Figure. 21. NDD KOs converge on mitochondrial function differently in iNPCs and mature iGLUTs.

SI Figure 22. Phenotypic clustering of 15 NDD genes in mutant zebrafish.

SI Figure 23. Transcriptomic clustering of 15 NDD genes in mature neurons with paired behavioral data in mutant zebrafish.

SI Figure 24. Drug Reversal of Behavioral Phenotypes in mutant zebrafish.

Supplemental Data

SI Data 1. Cell-type-specific convergent gene lists based on NDD pooled scCRISPR-KO.

SI Data 2. Convergent sets and drug predictions based on mutant zebrafish behavioral clusters.

SI Data 3. Gene x drug mutant zebrafish behavioral effects.

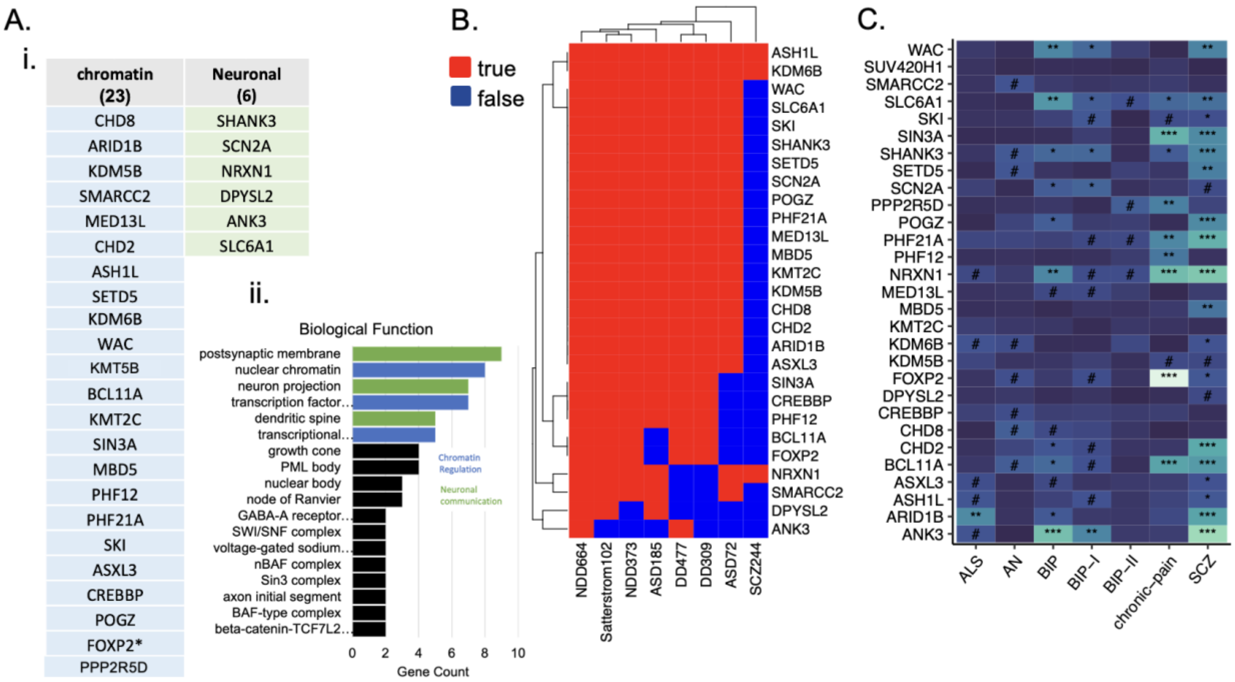

SI Figure 1. Characterization of 29 NDD rare variant genes. (A) (i) Broad functional classification of genes selected for NDD scCRISPR-KO pool screen based on **(ii)** over-representation analysis of gene-ontology biological function gene sets. **(B)** Heatmap of gene-disorder associations from exome-sequencing studies across neurodevelopmental disorders corresponding with **SI Table 1** (Developmental Delay (DD), Neurodevelopmental disorders (NDD), autism spectrum disorder (ASD), and schizophrenia (SCZ). **(C)** Corresponding individual gene-GWAS associations from one-sided MAGMA gene set enrichment analysis with FDR-based multiple testing correction. #nominal p-value<0.05, *FDR<0.05, **FDR<0.01, *FDR<0.001.

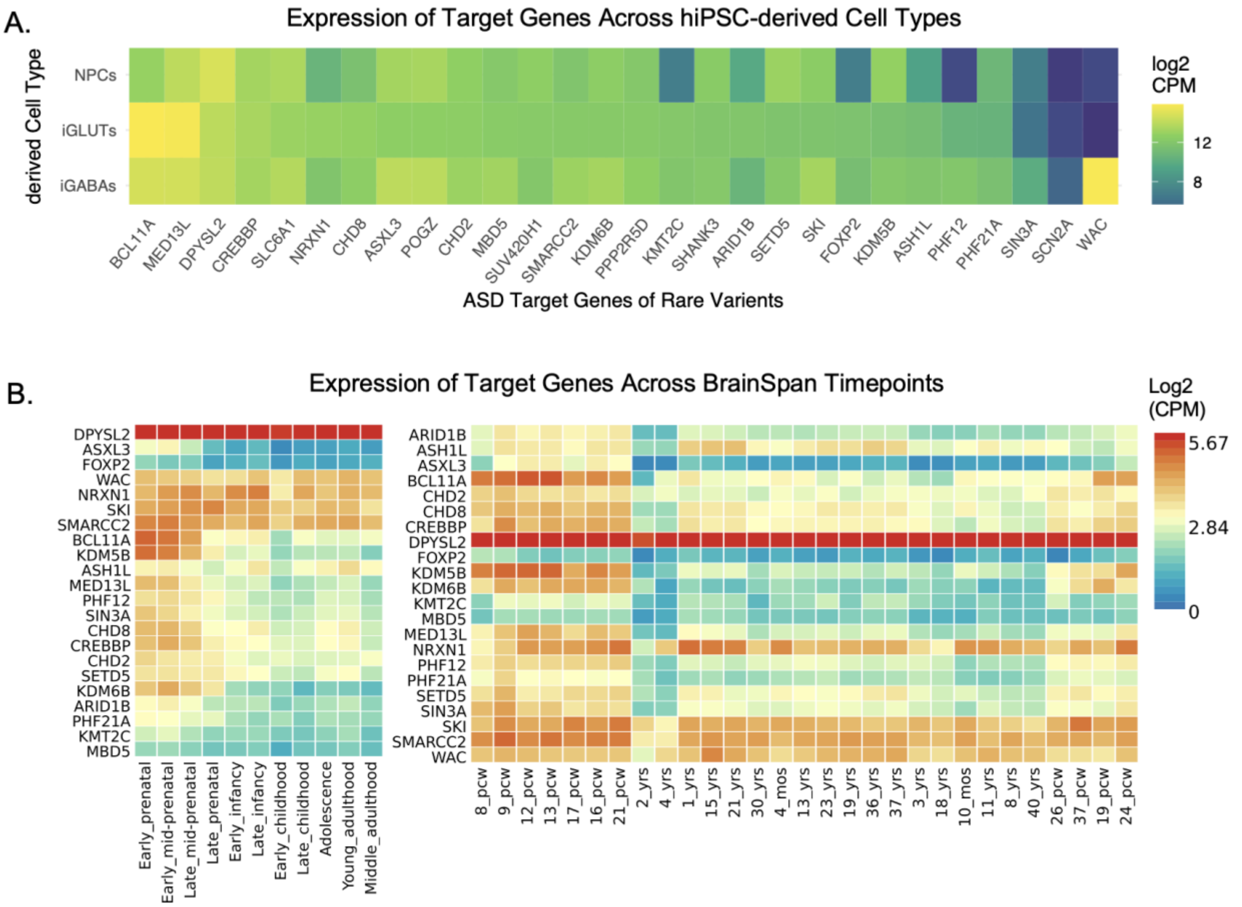

SI Figure 2. Characterization of gene expression of 29 NDD rare variant target genes in hiPSC neurons and the developing brain. (A) Normalized log2(CPM) of 29 NDD risk genes across NPCs, iGLUTs, and iGABAs (ordered by average expression in iGLUTs) and **(B)** BrainSpan developmental stages pulled using FUMA**.**

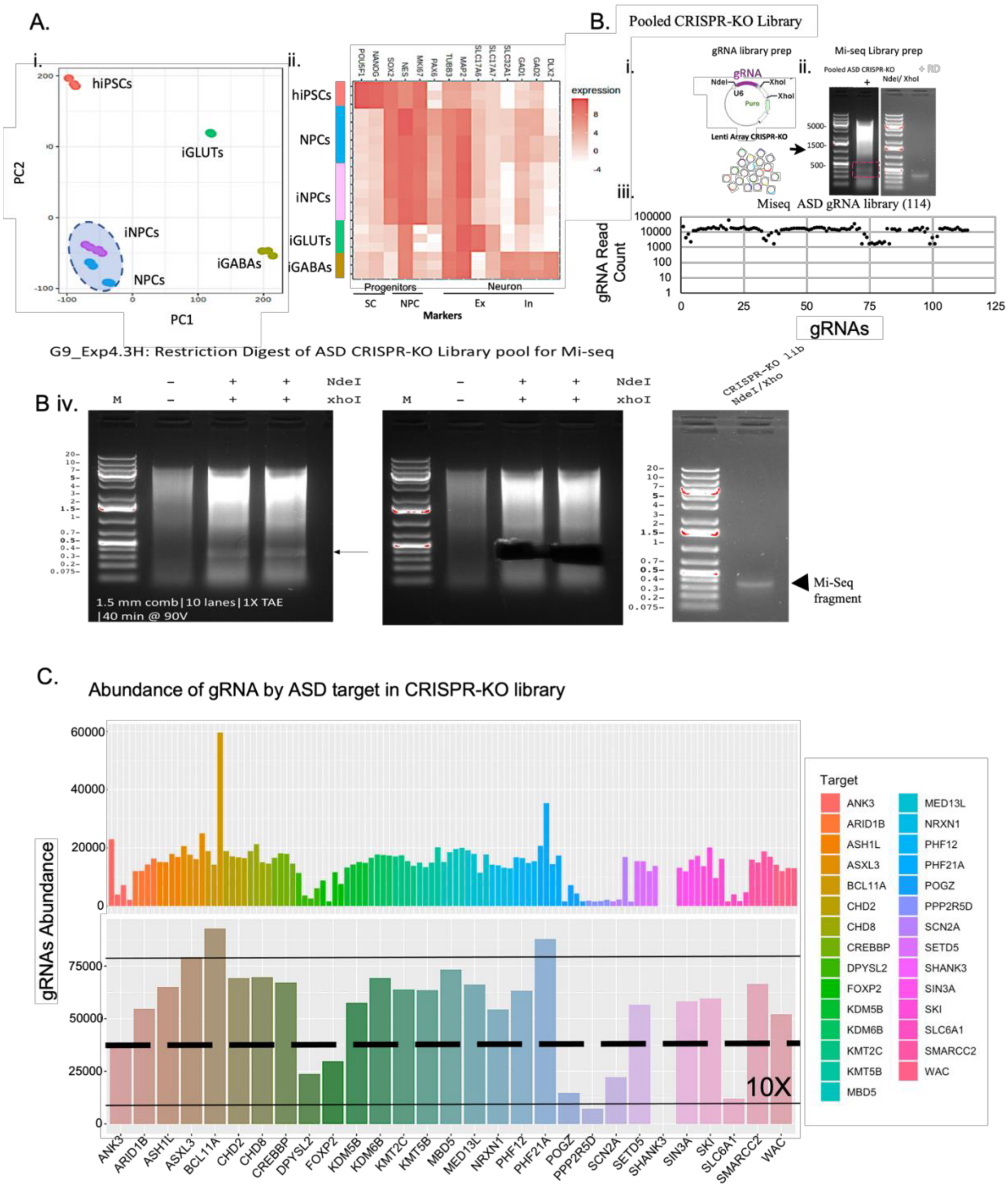

SI Figure 3. NDD pooled scCRISPR-KO screen validation. (A) Characterization of gene expression from clonalized lines of hiPSCs, NPCs/iNPCs, iGLUTs, and iGABAs by **(i)** PCA and **(ii)** average expression of cell-type specific markers. **(B)** Diagram of the gRNA plasmid insertion site and gel electrophoresis of cloned pooled library restriction digested (RD) for Mi-seq validation. **(C)** Frequency of ASD pooled gRNA (counts) in library by Mi-seq.

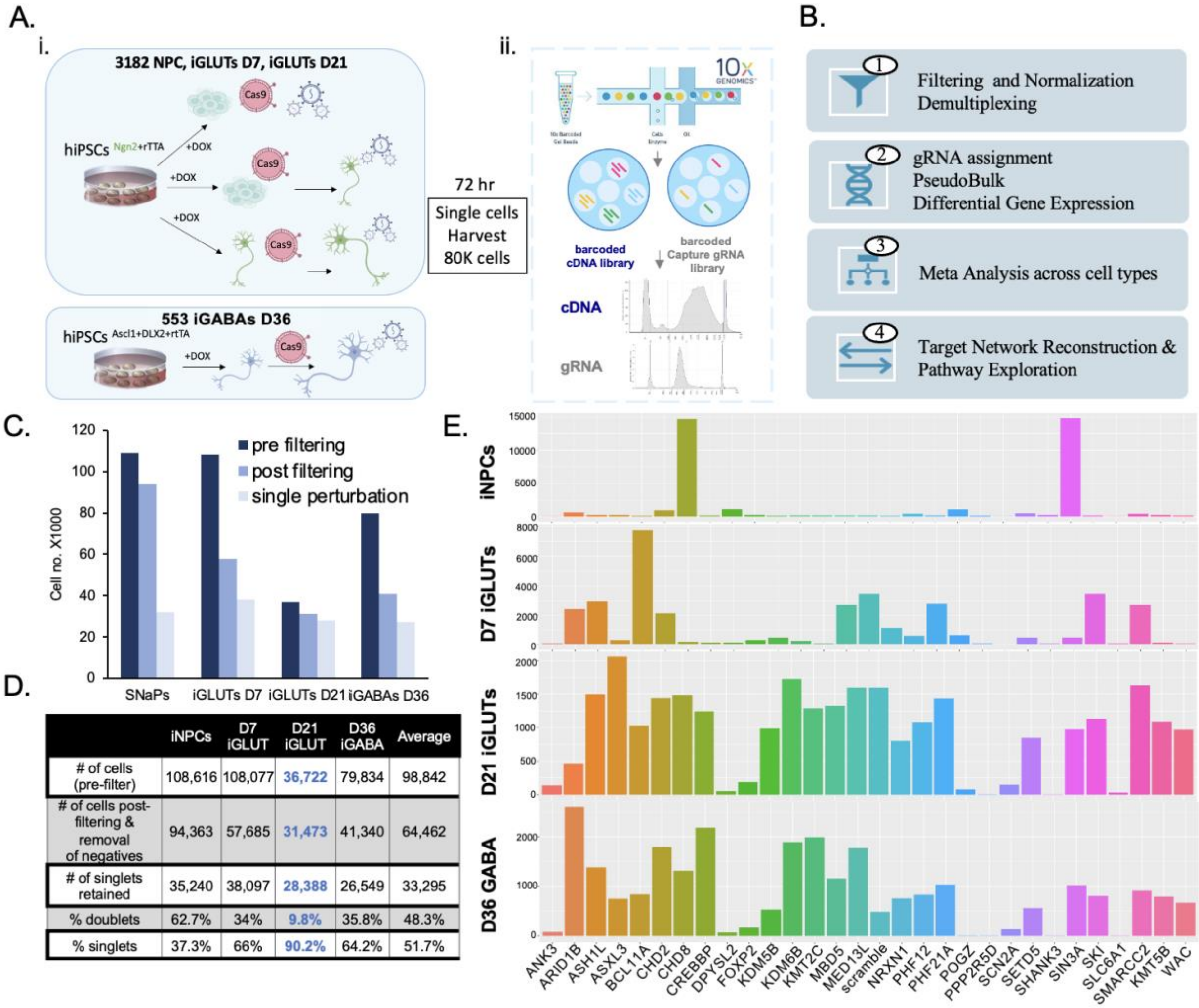

SI Figure 4. NDD scCRISPR-KO screen in hiPSC-derived iNPCs, iGLUTs, and iGABAs. (A) Illustration scCRISPR-KO screen experimental set-up. Note iNPCs and iGLUTs in control 1 (3182-3-clone5) and iGABAs in control 2 (553-3-clone34). **(B)** Analytical process following 10x sequencing. **(C)** Number of cells sequences (pre-filtering), post-filtering, and post selection for single gRNA demultiplexing across cell-types. **(D)** Table of number of cells – including percent singlets and doublets by experiment. **(E)** Frequency of cell gRNA identity by experiment.

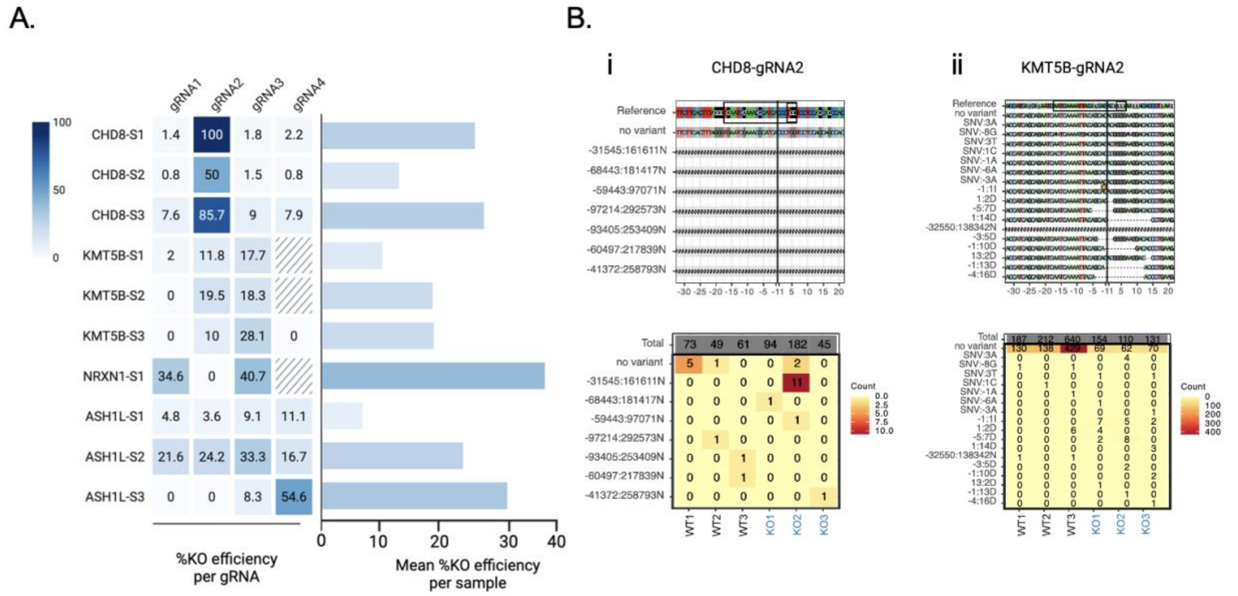

SI Figure 5. Validation of CRISPR-induced genome editing on selected genes with bulk RNAseq. **(A)** CRISPR-editing efficiency of gRNAs across 4 NDD genes using bulk RNAseq. Mean efficiency was calculated by average over all non-zero gRNAs of corresponding sample. Max efficiency was estimated assuming all editing events occur independently. **(B)** Representative variant plots of highest editing efficiency gRNAs for each (i) *CHD8* and (ii) *KMT5B*. Top panel, variants of gRNA-targeting loci, color-coded by nucleotide. Variants were identified based on CIGAR strings (including gaps) and are annotated on the left. The gRNA targeting site and PAM sequence are highlighted with brackets. The x-axis represents the relative position to the predicted Cas9 cut site. Bottom panels show the counts of each variant site in Cas9-induced (KO) and non-induced (WT) samples

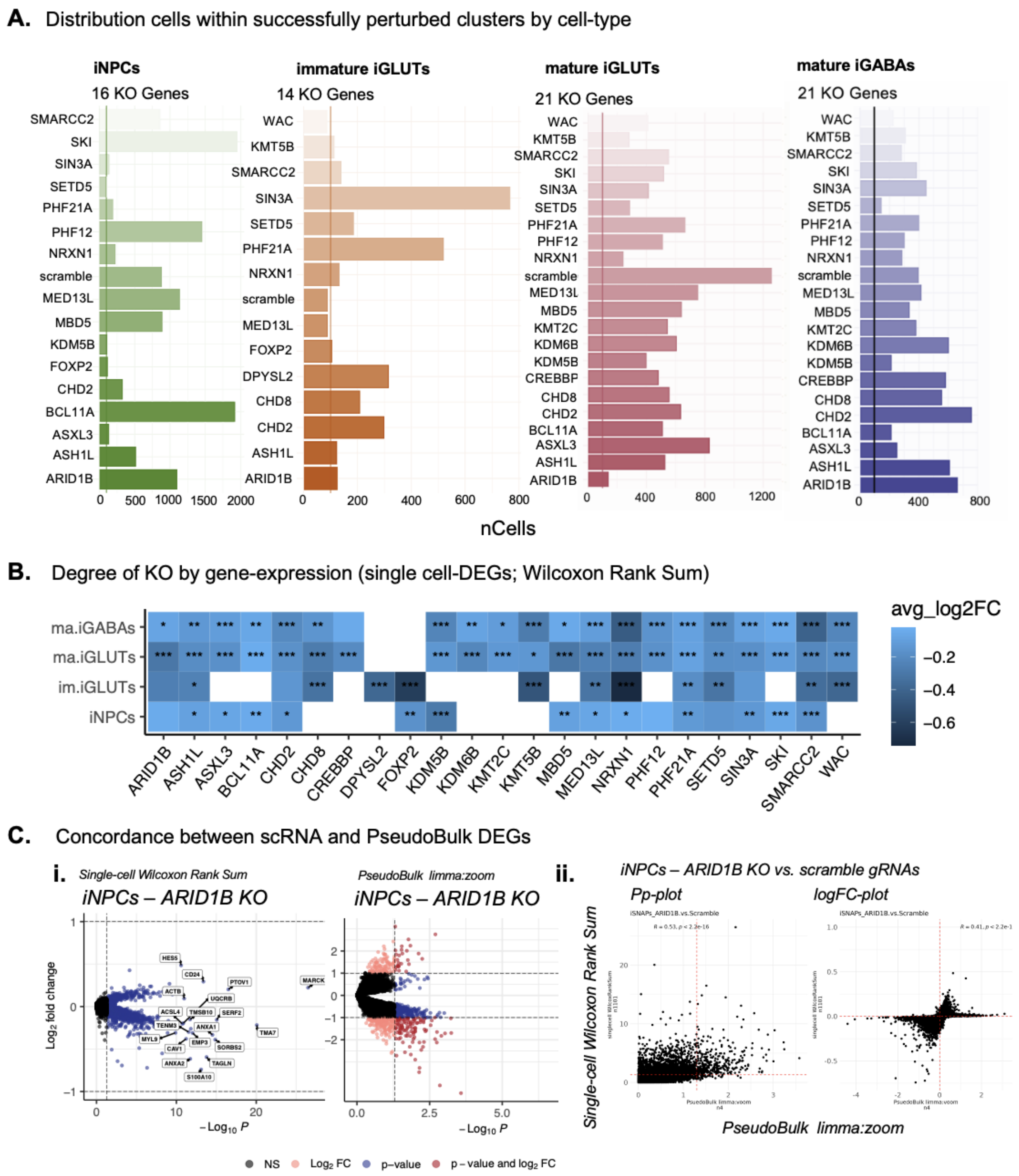

SI Figure 6. Characterization of perturbed cells and concordance between single-cell and pseudobulk differential gene expression analysis. (A) Number of singlet cells assigned as perturbed by gRNA per cell-type after performing WNN and filtering clusters distinct from non-targeting scramble control clusters. **(B)** Degree of logFC downregulation of target genes across cell-types from Wilcoxon and auROC DEG analysis in single-cell data shows that perturbed cells have lower expression of the targeted NDD gene. **(C)** Examples of the concordance of differential gene expression analysis using Wilcoxon Rank Sum test in single-cell data and limma:voom in pseudo-bulked data. **(i)** Volcano

plots of differential gene expression in *ARID1B* KO versus non-targeting samples in NPCs. **(ii)** Plots comparing p-values and logFC between differential gene expression analysis results at the single-cell level and pseudo-bulk level for *ARID1B*, *NRXN1*, and *CHD2* (Pearson's correlation, Holm's adjusted P-values).

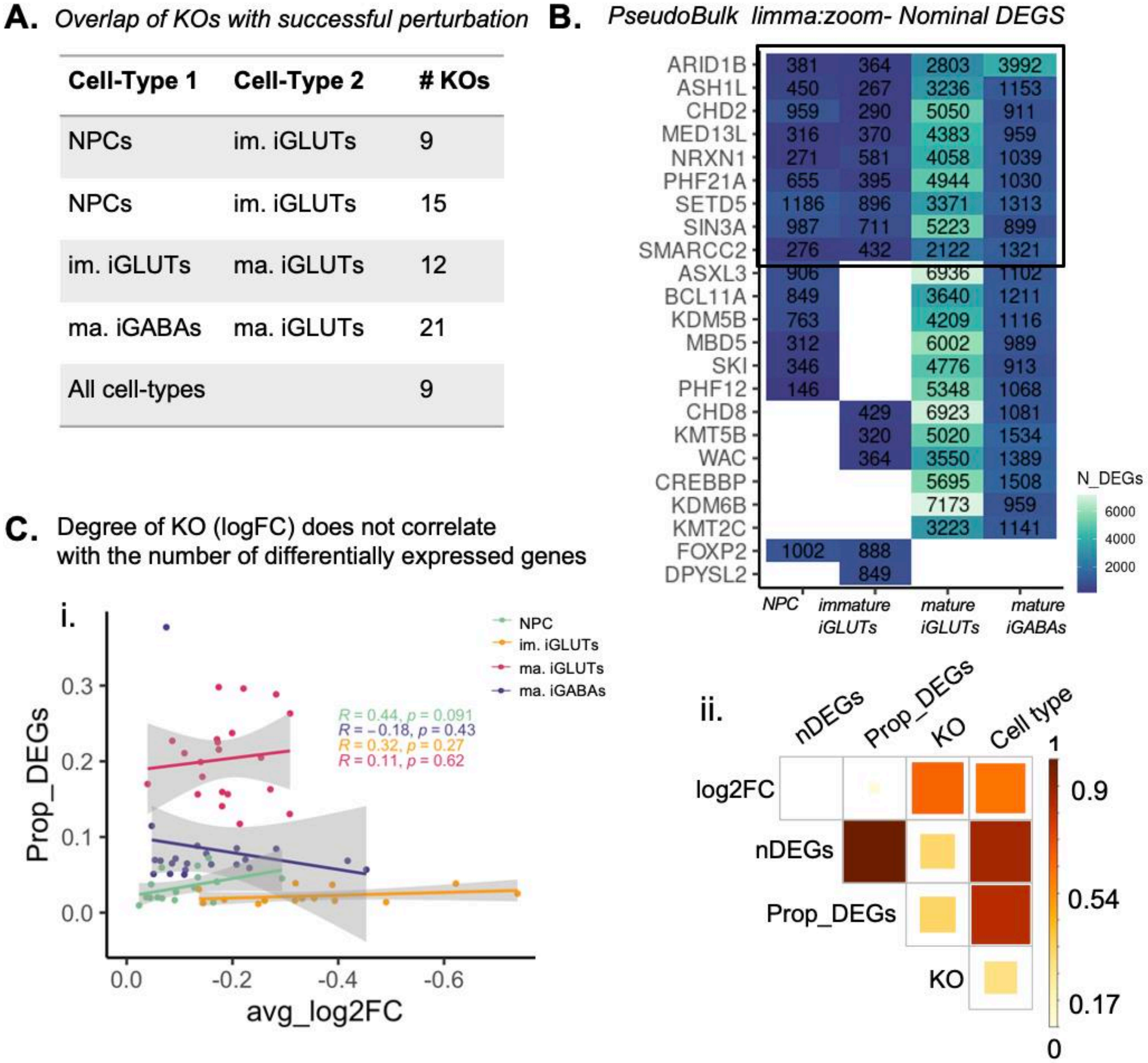

Cell-Type 1	Cell-Type 2	# KOs
NPCs	im. iGLUTs	9
NPCs	im. iGLUTs	15
im. iGLUTs	ma. iGLUTs	12
ma. iGABAs	ma. iGLUTs	21
All cell-types		9

SI Figure 7. Degree of down-regulation of KO expression does not significantly correlate with downstream transcriptomic impacts. (A) The number of KOs successfully resolved shared between cell-types - and thus allowing for direct comparison. **(B)** Transcriptomic impact of ASD/NDD gene KO across cell-type specific screens represented as the number of nominally significant ($p<0.01$) differentially expressed genes (DEGs). proportion of nominally significant DEGs in any cell-type (Pearson's correlation, Holm's adjusted P-values)**. (C)** There was no significant correlation between the average logFC of the target KO gene and the number of significantly differentially expressed genes. (i) Scatter plot of the average log2FC and proportion of differentially expressed genes (relative to the number of overall expressed gene) for each NDD KO colored by cell-type annotated with linear correlation coefficient and p-values. (ii) Heatmap of correlation coefficients across Cell type, KO gene, proportion of DEGS (prop_DEGs), absolute number of DEGs (nDEGs), and the log2FC of the corresponding KO gene.

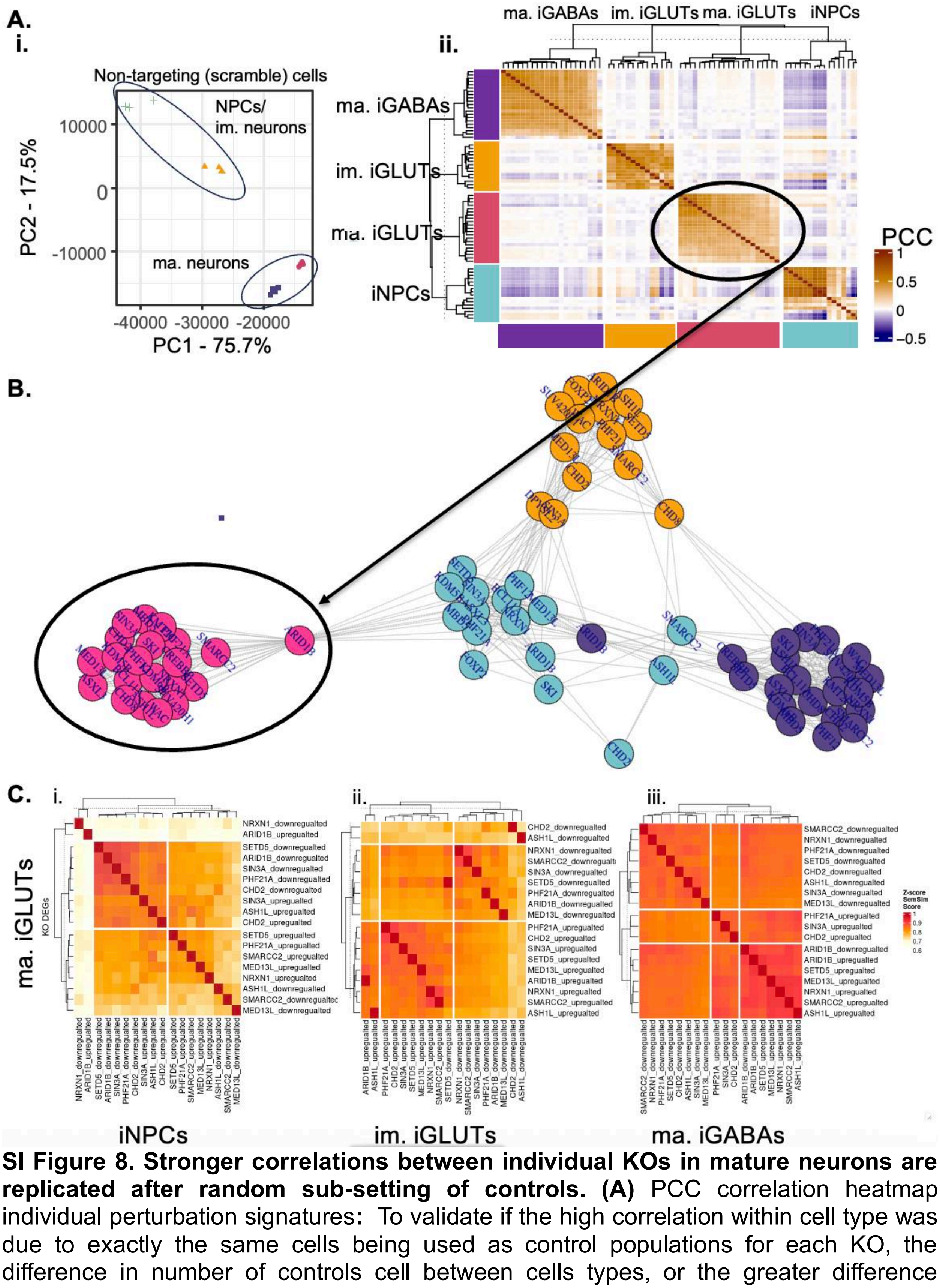

SI Figure 8. Stronger correlations between individual KOs in mature neurons are replicated after random sub-setting of controls. (A) PCC correlation heatmap individual perturbation signatures**:** To validate if the high correlation within cell type was due to exactly the same cells being used as control populations for each KO, the difference in number of controls cell between cells types, or the greater difference

between controls in immature cell-types as indicated by PCA **(i) d**ifferential expression was performed between KOs and randomly selected scramble controls. Briefly, for each gene, 50% (if number of pseudobulked sample cells > 50) or 80% (if number of pseudobulked sample cells < 50) of scramble cells were randomly selected differential expression analysis re-run. The process was repeated three times to avoid random selection bias and median of each gene logFC was used as the final logFC. Average overlap of random scramble cells across different genes is approximately 50%. (**ii)** Pearson's correlation matrix of log2FC across all KOs was then calculated and plotted as heatmap. Greatest similarity remained within same cell type as when using all scramble controls. (**B)** PCC correlation network of individual perturbation signatures: Cross cell-type correlation network diagram across KO perturbations. Mature iGLUTs remained most isolated from the other three cell types, similar to what was observed in DEGs using all scramble controls. **(C)** Heatmap of z-score values of semantic similarity based on biological pathway membership of the significantly up and down-regulated DEGs for each KO compared. (iii) Mature iGABA and iGLUT KO target DEGs are the most functionally similar while (i) iNPC and mature iGLUTs KO DEGs are the least functionally similar.

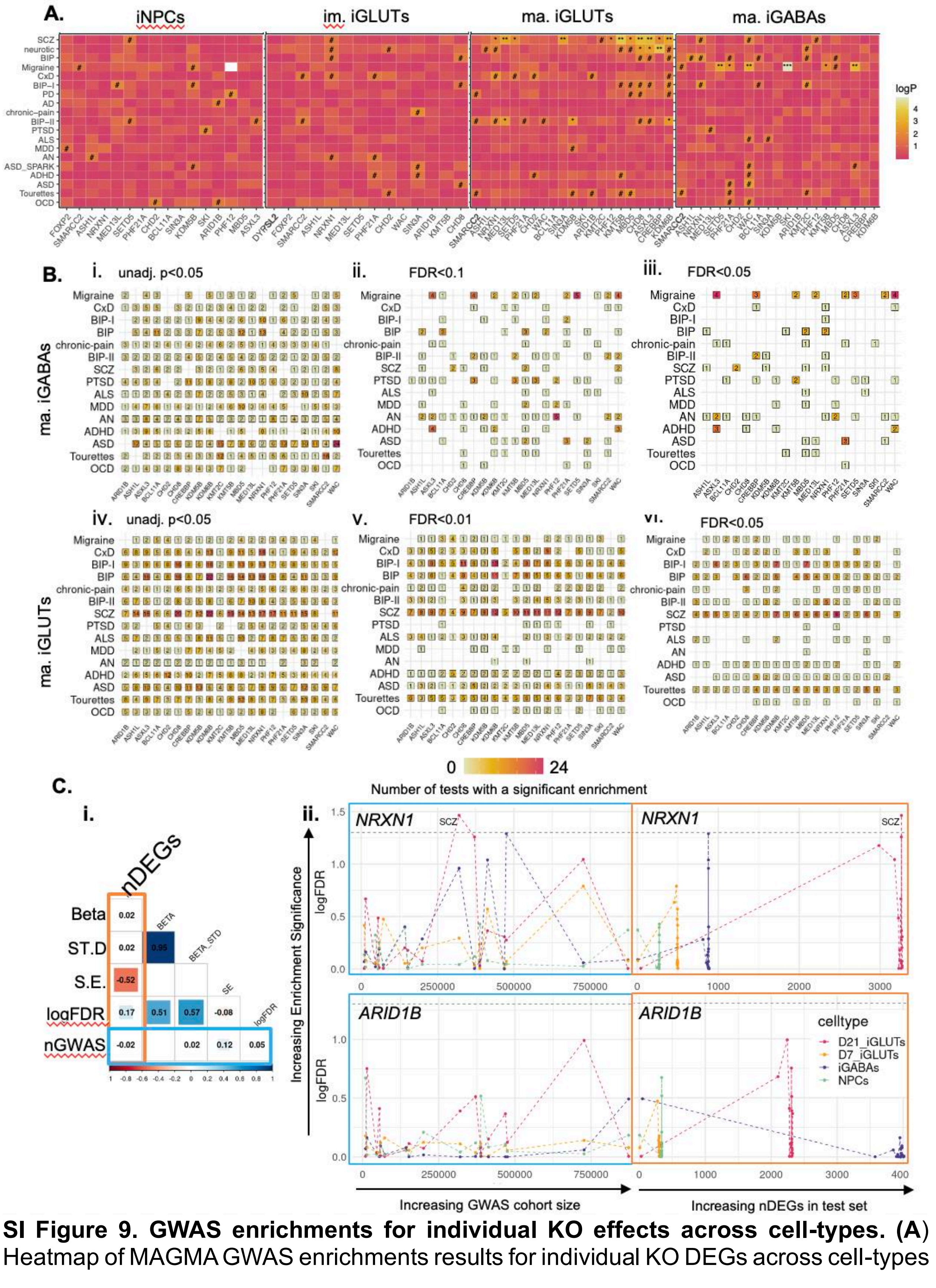

SI Figure 9. GWAS enrichments for individual KO effects across cell-types. (A) Heatmap of MAGMA GWAS enrichments results for individual KO DEGs across cell-types

reveals convergence on SCZ common variant risk genes in mature iGLUTs. **(B)** To test if this effect was due to SCZ risk genes being generally enriched in neurons or due to the size of the genesets for each GWAS – GWAS sets were filtered for genes expressed in each cell-type prior to enrichment testing and enrichment tests were performed after randomly down-sampling GWAS GeneSets to 100, 250, 500, 750, and 1000 genes. This process was then performed 10 times within each set size (i.e 50 tests for each GWAS). Strong associations with individual KO effects and SCZ and Bipolar genes remained across enrichments tests in mature iGLUTs. While strong association with migraine remained in iGABAs. Frequency tables showing the number of randomly down-sampled enrichment tests that were significant between KO DEGs and GWAS genesets across three significance thresholds in mature iGABAs and IGLUTs: **(i,iv)** unadjusted p-value <0.05, **(ii,v)** FDR <0.01, and **(iii,vi)** FDR<0.05. GWAS labels on the y-axis are order by the overall sample size from the GWAS studies. **(C)** **(i)** Since it is possible that GWAS sample size (nGWAS) and the number of DEGs (nDEGs) in a set may affect enrichment testing, we tested correlation between enrichment results (beta, standard deviation (ST.D), standard error (S.E) and found no significant correlations between enrichment significance and GWAS or gene set size. **(ii)** Scatter line plots visualizing relationships between GWAS cohort size and number of DEGs in enrichment sets for *NRXN1* and *ARID1B*.

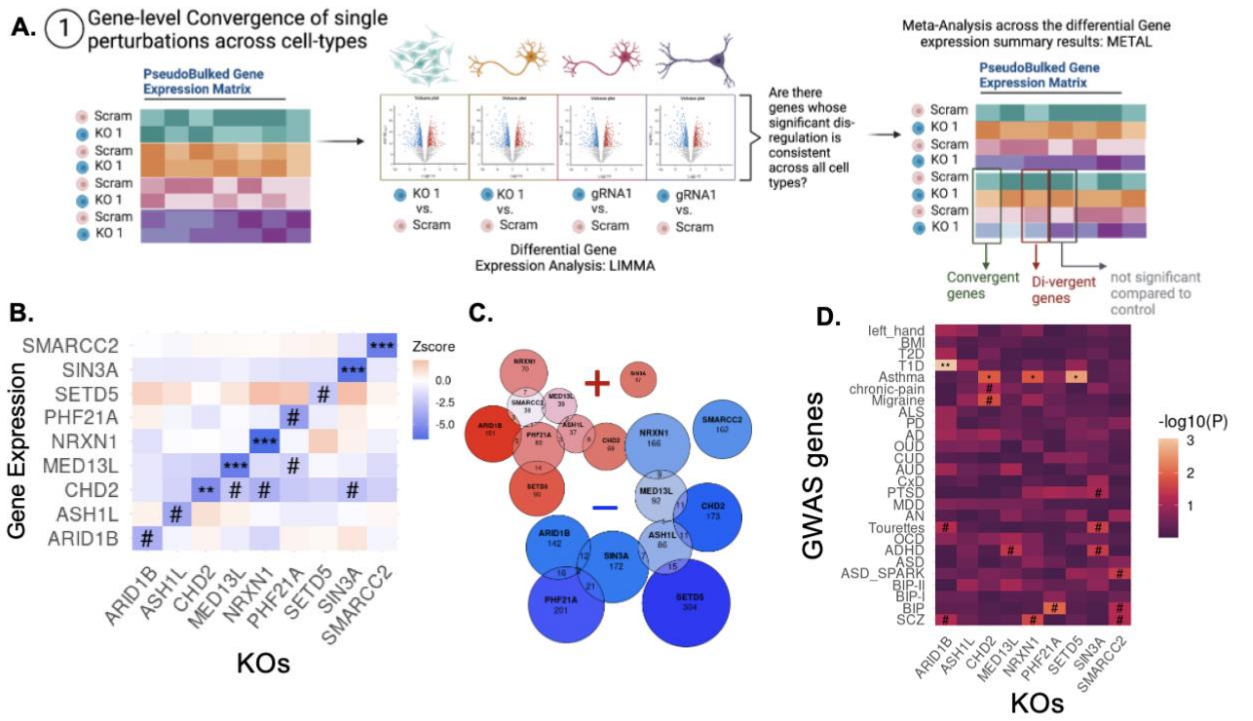

SI Figure 10. NDDs show little transcriptomic convergence across cell-types and maturity. (A) Schematic explaining KO-specific cross cell-type convergence at the individual gene level using differential gene expression meta-analysis. Created in BioRender. Townsley, K. (2026) https://BioRender.com/efkzzf6. **(B)** Heatmap of meta-analyzed z-score effects on gene expression of targeted KO eGenes across perturbation phenotypes (#, unadjusted p-value =<0.05, *FDR<=0.05, **FDR<0.01, ***FDR<0.001). **(C)** Euler diagrams of overlapping convergent genes by eGene KO phenotype (nominal p-value <=0.05; Cochran's Heterogeneity p-value > 0.05; positive (red) or (negative) direction of effect across all cell types). **(D)** MAGMA enrichment analysis of KO-specific cross-cell type convergence and GWAS-risk associated genes.

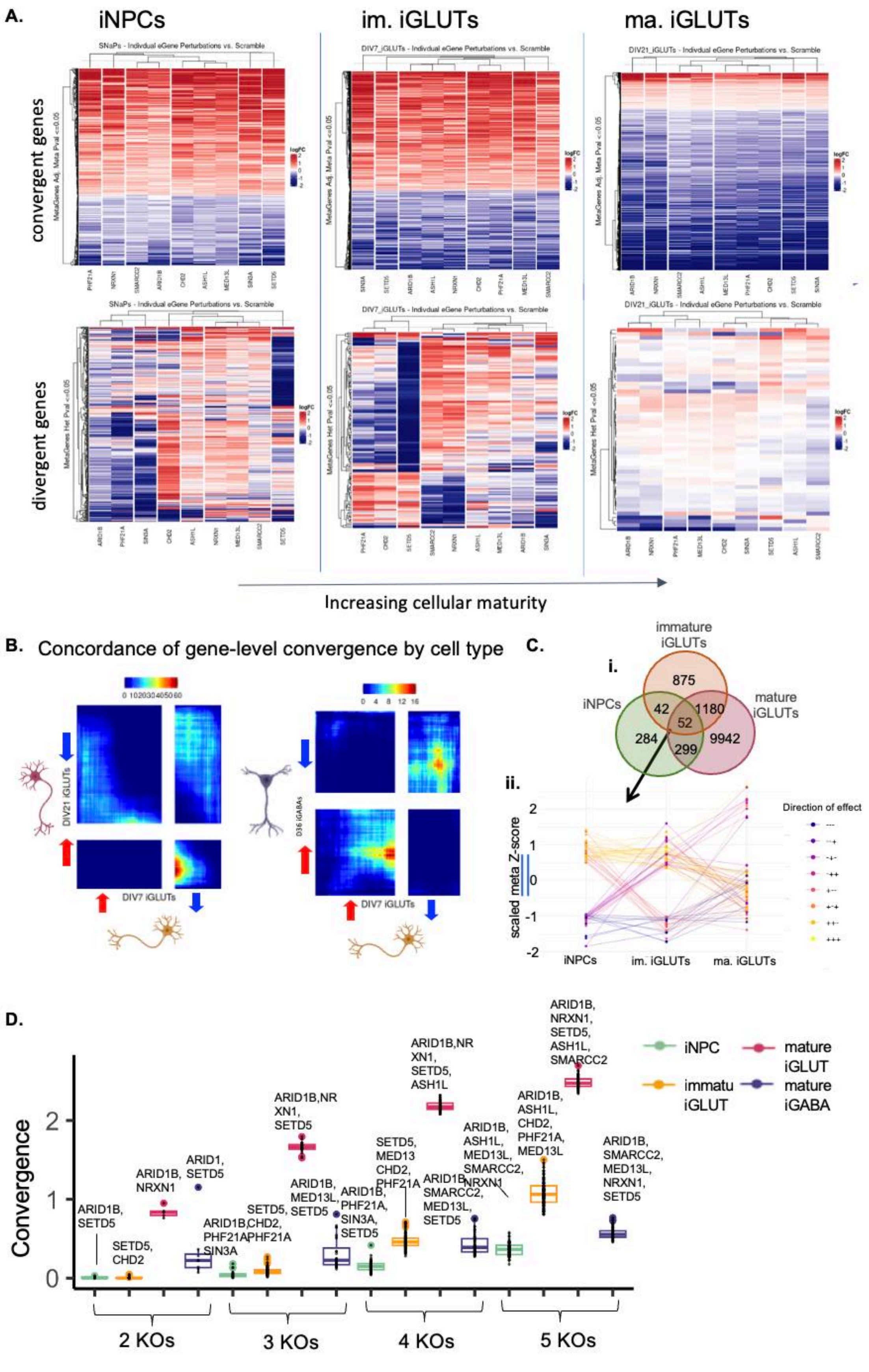
A.
iNPCs
im. iGLUTs
ma. iGLUTs
convergent genes
divergent genes
Increasing cellular maturity
B.
Concordance of gene-level convergence by cell type
DIV21 iGLUTs
DIV7 iGLUTs
D36 iGABAs
C.
i.
immature iGLUTs
iNPCs
mature iGLUTs
875
42
1180
52
284
299
9942
ii.
scaled meta Z-score
Direction of effect
iNPCs
im. iGLUTs
ma. iGLUTs
D.
Convergence
iNPC
immatu iGLUT
mature iGLUT
mature iGABA
2 KOs
3 KOs
4 KOs
5 KOs

SI Figure 11. Gene-level convergence across 9 NDDs is unique across cell-types and increases with maturity in iGLUT neurons. (A) Heatmaps of convergent and divergent genes across 9 individual KOs in NPCs, immature iGLUTs, and mature iGLUTs). **(B)** Comparison over transcriptomic wide meta pFDR and z-scores using RRHO correlation across immature neurons (iGLUTs) and mature neurons (iGLUTs and iGABAs). **(C)** (i) Overlap of convergent genes across NPCs, immature iGLUT, and mature iGLUTs and (ii) their direction of effect in each cell-type. Scaled Z-scores of the 52 overlapping convergent genes across cell-type show that they have unique directions of effect. **(D)** The top-most convergent KO combinations were unique to each cell-type. Boxplot of convergence magnitude for every tested combination of NDD genes ranging for sets of 2KOs to 5 KOs with the top convergent sets annotated.

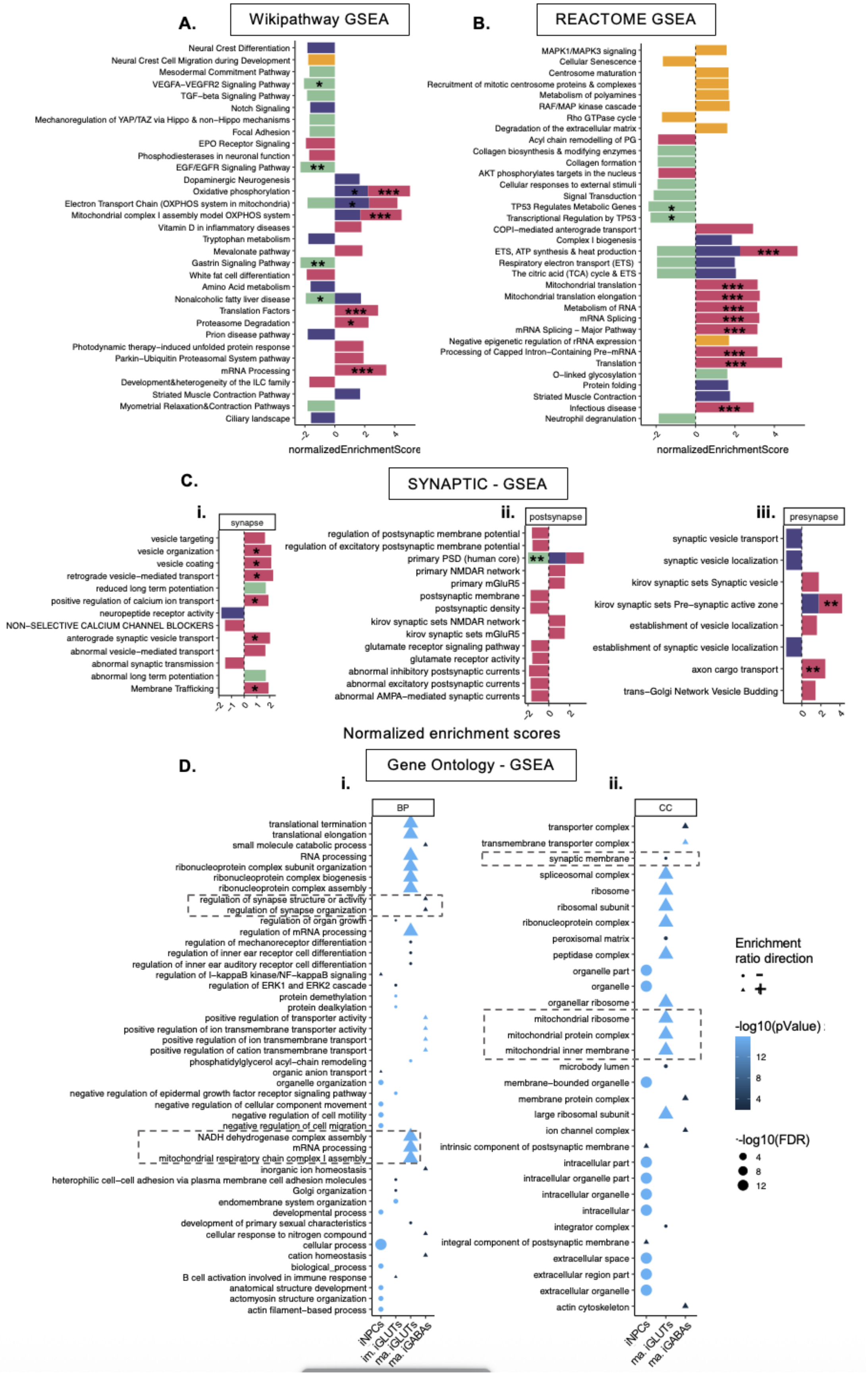

A.
Wikipathway GSEA
Neural Crest Differentiation
Neural Crest Cell Migration during Development
Mesodermal Commitment Pathway
VEGFA−VEGFR2 Signaling Pathway
TGF−beta Signaling Pathway
Notch Signaling
Mechanoregulation of YAP/TAZ via Hippo & non−Hippo mechanisms
Focal Adhesion
EPO Receptor Signaling
Phosphodiesterases in neuronal function
EGF/EGFR Signaling Pathway
Dopaminergic Neurogenesis
Oxidative phosphorylation
Electron Transport Chain (OXPHOS system in mitochondria)
Mitochondrial complex I assembly model OXPHOS system
Vitamin D in inflammatory diseases
Tryptophan metabolism
Mevalonate pathway
Gastrin Signaling Pathway
White fat cell differentiation
Amino Acid metabolism
Nonalcoholic fatty liver disease
Translation Factors
Proteasome Degradation
Prion disease pathway
Photodynamic therapy−induced unfolded protein response
Parkin−Ubiquitin Proteasomal System pathway
mRNA Processing
Development&heterogeneity of the ILC family
Striated Muscle Contraction Pathway
Myometrial Relaxation&Contraction Pathways
Ciliary landscape
normalizedEnrichmentScore
B.
REACTOME GSEA
MAPK1/MAPK3 signaling
Cellular Senescence
Centrosome maturation
Recruitment of mitotic centrosome proteins & complexes
Metabolism of polyamines
RAF/MAP kinase cascade
Rho GTPase cycle
Degradation of the extracellular matrix
Acyl chain remodelling of PG
Collagen biosynthesis & modifying enzymes
Collagen formation
AKT phosphorylates targets in the nucleus
Cellular responses to external stimuli
Signal Transduction
TP53 Regulates Metabolic Genes
Transcriptional Regulation by TP53
COPI−mediated anterograde transport
Complex I biogenesis
ETS, ATP synthesis & heat production
Respiratory electron transport (ETS)
The citric acid (TCA) cycle & ETS
Mitochondrial translation
Mitochondrial translation elongation
Metabolism of RNA
mRNA Splicing
mRNA Splicing − Major Pathway
Negative epigenetic regulation of rRNA expression
Processing of Capped Intron−Containing Pre−mRNA
Translation
O−linked glycosylation
Protein folding
Striated Muscle Contraction
Infectious disease
Neutrophil degranulation
normalizedEnrichmentScore
C.
SYNAPTIC - GSEA
i.
synapse
vesicle targeting
vesicle organization
vesicle coating
retrograde vesicle−mediated transport
reduced long term potentiation
positive regulation of calcium ion transport
neuropeptide receptor activity
NON−SELECTIVE CALCIUM CHANNEL BLOCKERS
anterograde synaptic vesicle transport
abnormal vesicle−mediated transport
abnormal synaptic transmission
abnormal long term potentiation
Membrane Trafficking
ii.
postsynapse
regulation of postsynaptic membrane potential
regulation of excitatory postsynaptic membrane potential
primary PSD (human core)
primary NMDAR network
primary mGluR5
postsynaptic membrane
postsynaptic density
kirov synaptic sets NMDAR network
kirov synaptic sets mGluR5
glutamate receptor signaling pathway
glutamate receptor activity
abnormal inhibitory postsynaptic currents
abnormal excitatory postsynaptic currents
abnormal AMPA−mediated synaptic currents
iii.
presynapse
synaptic vesicle transport
synaptic vesicle localization
kirov synaptic sets Synaptic vesicle
kirov synaptic sets Pre−synaptic active zone
establishment of vesicle localization
establishment of synaptic vesicle localization
axon cargo transport
trans−Golgi Network Vesicle Budding
Normalized enrichment scores
D.
Gene Ontology - GSEA
i.
BP
translational termination
translational elongation
small molecule catabolic process
RNA processing
ribonucleoprotein complex subunit organization
ribonucleoprotein complex biogenesis
ribonucleoprotein complex assembly
regulation of synapse structure or activity
regulation of synapse organization
regulation of organ growth
regulation of mRNA processing
regulation of mechanoreceptor differentiation
regulation of inner ear receptor cell differentiation
regulation of inner ear auditory receptor cell differentiation
regulation of I−kappaB kinase/NF−kappaB signaling
regulation of ERK1 and ERK2 cascade
protein demethylation
protein dealkylation
positive regulation of transporter activity
positive regulation of ion transmembrane transporter activity
positive regulation of ion transmembrane transport
positive regulation of cation transmembrane transport
phosphatidylglycerol acyl−chain remodeling
organic anion transport
organelle organization
negative regulation of epidermal growth factor receptor signaling pathway
negative regulation of cellular component movement
negative regulation of cell motility
negative regulation of cell migration
NADH dehydrogenase complex assembly
mRNA processing
mitochondrial respiratory chain complex I assembly
inorganic ion homeostasis
heterophilic cell−cell adhesion via plasma membrane cell adhesion molecules
Golgi organization
endomembrane system organization
developmental process
development of primary sexual characteristics
cellular response to nitrogen compound
cellular process
cation homeostasis
biological_process
B cell activation involved in immune response
anatomical structure development
actomyosin structure organization
actin filament−based process
iNPCs
im. iGLUTs
ma. iGLUTs
ma. iGABAs
ii.
CC
transporter complex
transmembrane transporter complex
synaptic membrane
spliceosomal complex
ribosome
ribosomal subunit
ribonucleoprotein complex
peroxisomal matrix
peptidase complex
organelle part
organelle
organellar ribosome
mitochondrial ribosome
mitochondrial protein complex
mitochondrial inner membrane
microbody lumen
membrane−bounded organelle
membrane protein complex
large ribosomal subunit
ion channel complex
intrinsic component of postsynaptic membrane
intracellular part
intracellular organelle part
intracellular organelle
intracellular
integrator complex
integral component of postsynaptic membrane
extracellular space
extracellular region part
extracellular organelle
actin cytoskeleton
iNPCs
ma. iGLUTs
ma. iGABAs
Enrichment ratio direction
−
+
-log10(pValue)
12
8
4
-log10(FDR)
4
8
12

SI Figure 12. Gene-level convergence across 9 NDDs is enriched for pathways involved in neural development, mitochondrial function, and translational regulation. Two-sided gene set enrichment analysis of gene-level convergence across 9 NDD KOs (*ARID1B, ASH1L, CHD2, MED13L, NRXN1, PHF21A, SETD5, SIN3A, SMARCC2*) for each cell-type was performed using the **(A)** WikiPathway gensets, **(B)** Reactome genesets, **(C)** curated synaptic genesets, and **(D)** gene ontology pathways. Enrichments were filtered for nominal significance (unadjusted p-value <=0.05); FDR significance is indicated through annotations [FDR<=0.05*. FDR<=0.01**, FDR<=0.001***] (A-C) or size of the points.

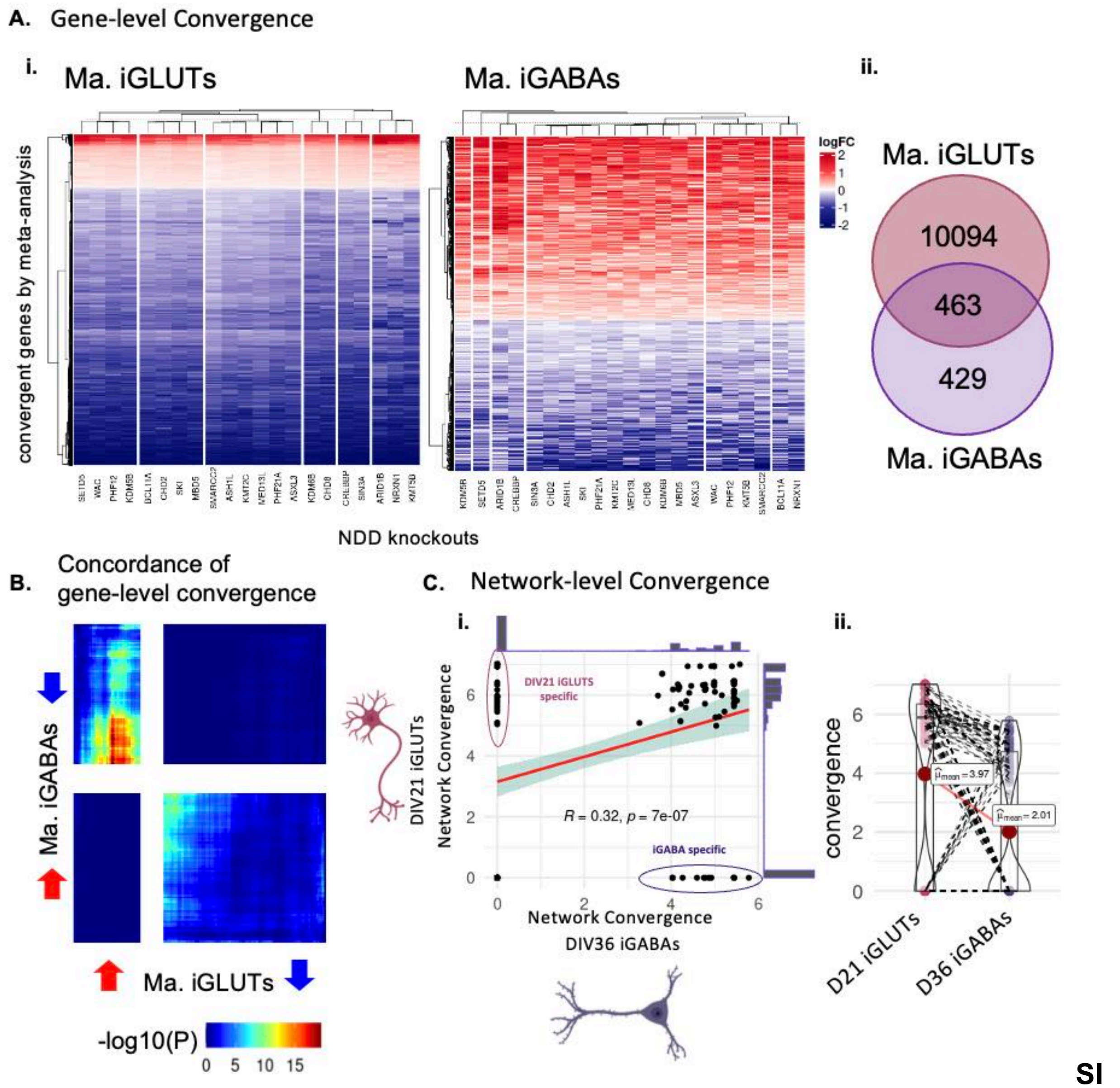

SI

Figure 13. Gene-level and network-level convergence across 21 NDDs in iGLUT and iGABA neurons. (A) (i) Heatmaps of convergent and divergent genes across 21 NDD KOs between mature iGLUTs and iGABAs **(ii)** Overlap of convergent genes across mature iGLUTs and iGABAs. **(B)** Comparison over transcriptomic wide meta pFDR and z-scores using RRHO correlation between mature iGLUTs and iGABAs. **(C)** Comparison of the strength of network level convergence across different KO combinations – **(i)** While network convergence was generally strongly correlated (2-tailed Pearson's correlation: Pearson's R=0.32, $p=7\times10^{-7}$) between neuronal types, some cell-type specific networks exist and **(ii)** mature iGLUTs had stronger mean convergence across all networks (paired Student's T-test).

A.

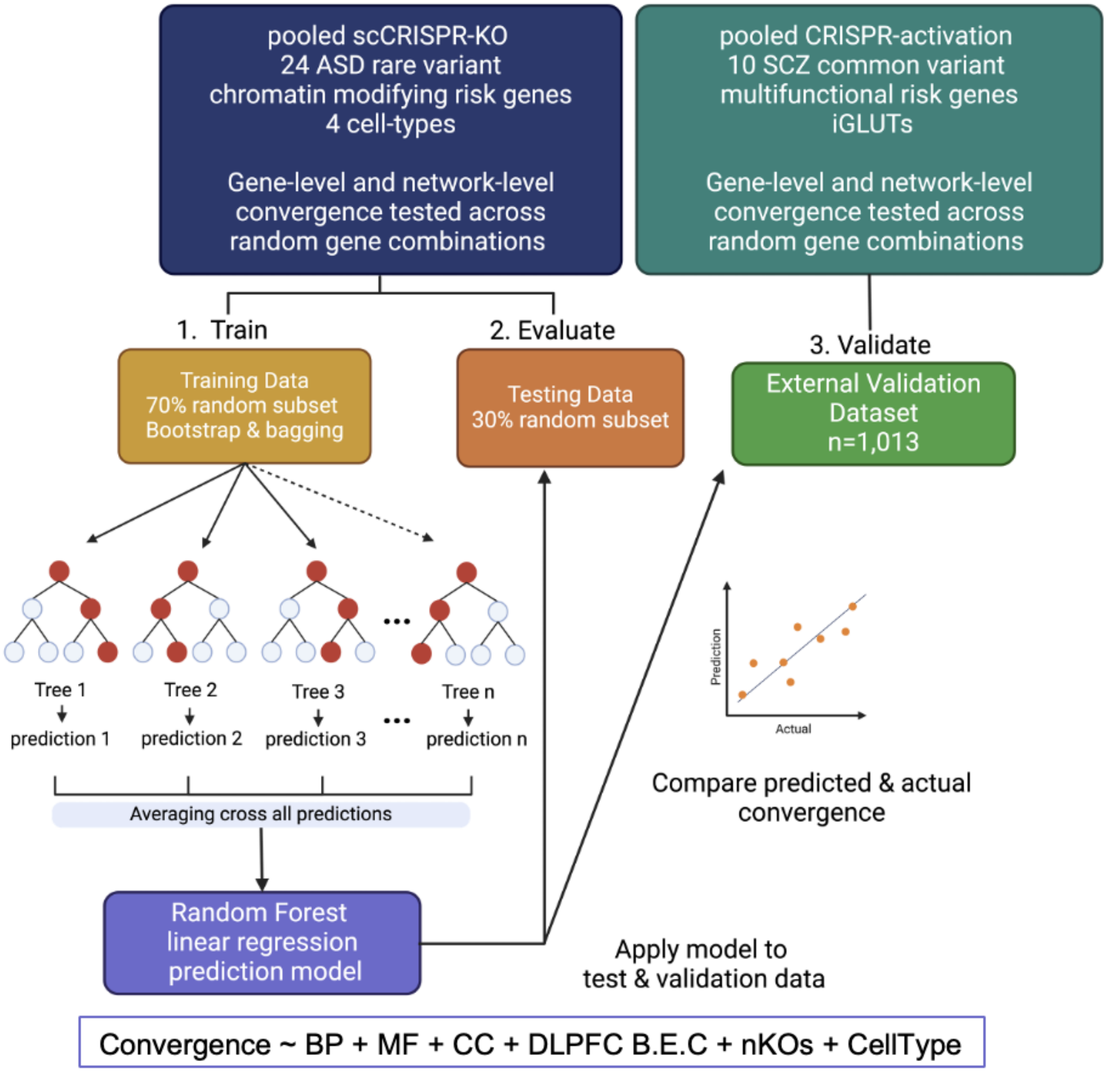

B. Number of unique NDD KO combinations tested for gene and network-level convergence

Cell-Type	# random network combinations tested (n2-10 KOs)	# network combinations resolved (n2-10 KOs)	# random gene-level combinations tested (n2-5 KOs)
NPCs	900	149 (16.6%)	2724
Immature iGLUTs	900	594 (66%)	3458
mature iGLUTs	1800	1314 (73%)	6150
mature iGABAs	1800	1150 (64%)	7906

SI Figure 14. Schema of random forest training model and external validation. (A) Training random forest models for gene and network-level convergence. Created in BioRender. Townsley, K. (2026) https://BioRender.com/5xlpxkm. **(B)** Number of unique ASD KO combinations tested for gene and network-level convergence and used for random forest predictions. (***B.P score*** = semantic similarity of gene ontology (GO): Biological Process membership between KO genes; ***C.C. score*** = semantic similarity of GO: Cellular Component membership between KO genes; ***M.F score*** = semantic similarity of GO: Molecular functions membership between KO genes; ***B.E.C*** =

dorsolateral prefrontal cortex expression correlations between KO genes; ***nKOs*** = number of KO genes tested for convergence). Corresponds with the analysis in **Figure 5**.

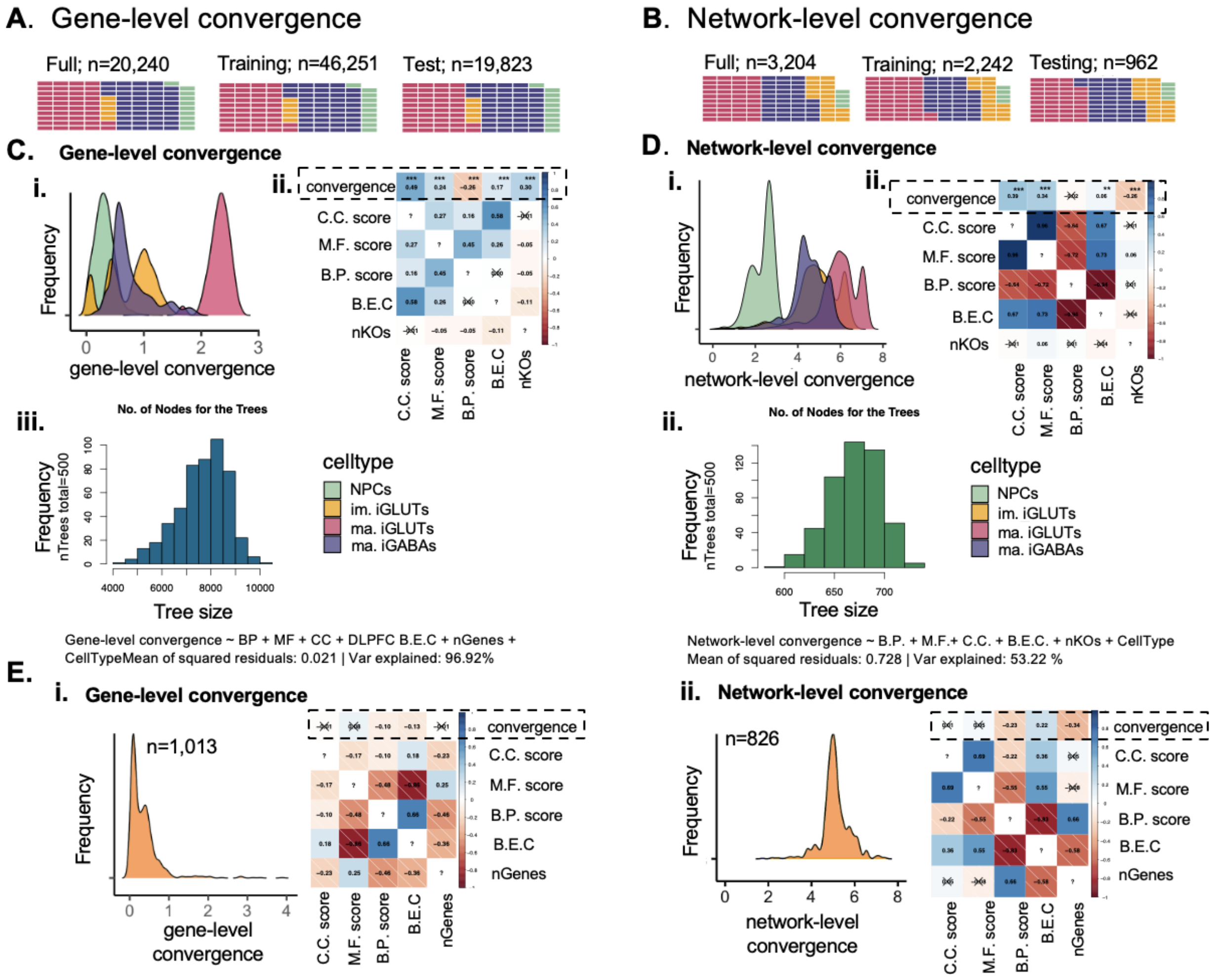

SI Figure 15. Random forest training data characteristics and node frequency across trees. (A-B) Functional similarity, brain co-expression, cell-type, and the number of KOs assayed strongly predicted gene-level convergence (98% variance explained; mean of squared residuals=0.02) and moderately predicted network-level convergence (53% variance explained; mean of squared residuals=0.73). Correlation of predictor variables and gene-level **(A-B)** The proportion of cell-types is balanced across the training sets and the testing sets and is representative the full data. **(C, D)** Distribution of gene-level **(C,i)** and network-level **(D,i)** convergence scores for each cell-type. Correlation of predictor variables and gene-level **(C,ii)** and network-level **(D,ii)** convergence. Number of nodes per tree in the random forest models (nTrees total=500) for **(C,iii)** gene-level convergence and for **(D,iii)** network-level convergence. **(E)** Validation of predictor models in an independent scCRISPRa screen of SCZ target genes. Distribution of convergence scores and their correlation with predictor variables at the gene **(E,i)** and network-level **(E,ii).** (***B.P score*** = semantic similarity of gene ontology (GO): Biological Process membership between KO genes; ***C.C. score*** = semantic similarity of GO: Cellular Component membership between KO genes; ***M.F score*** = semantic similarity of GO: Molecular functions membership between KO genes; ***B.E.C*** = dorsolateral prefrontal cortex expression correlations between KO genes; ***nKOs*** = number of KO genes tested for convergence). Data corresponds with the analysis in **Figure 5**.

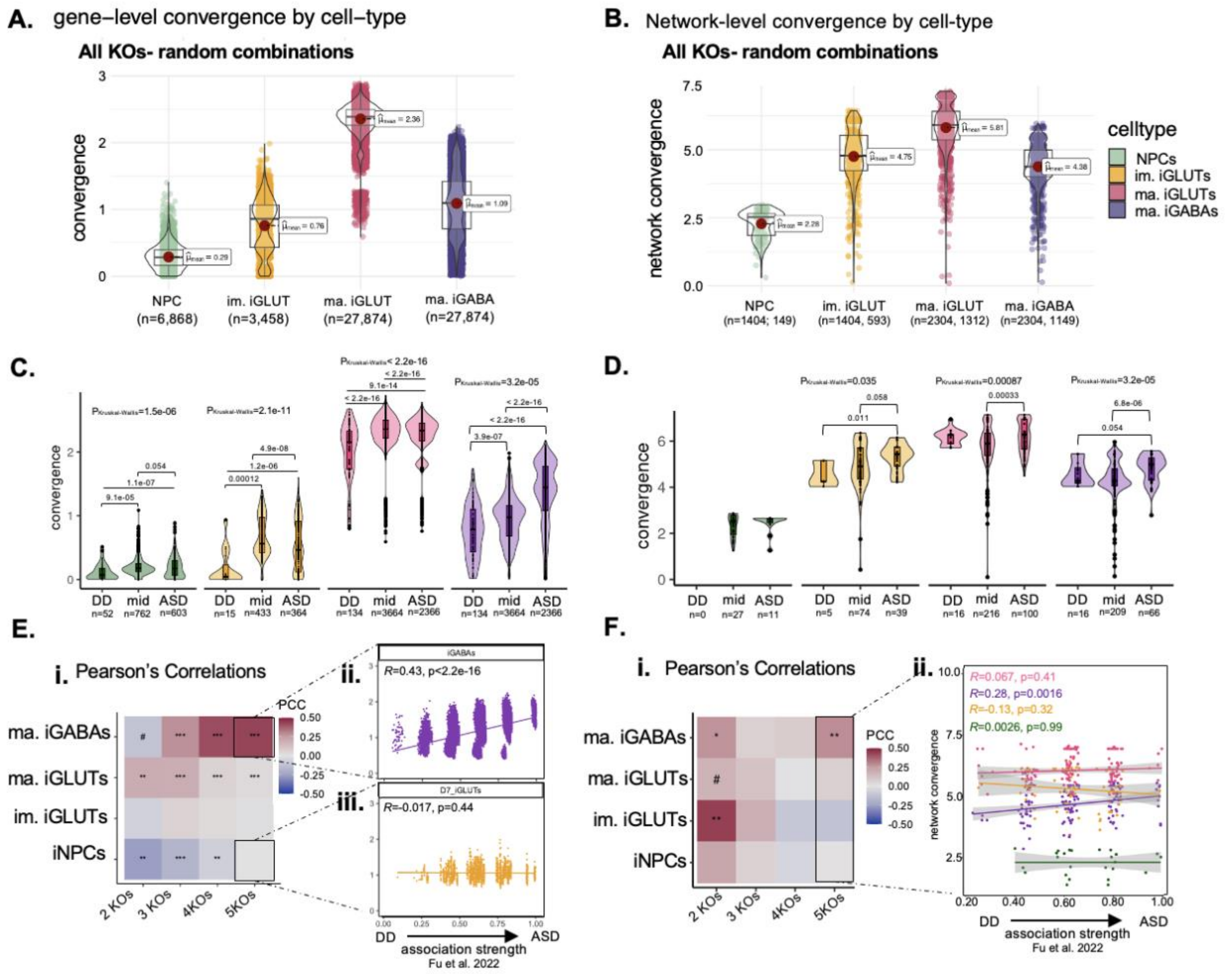

SI Figure 16. NDD risk genes with strong ASD associations resolve greater convergence across cell-types. (A) Gene-level and **(B)** network-level convergence remain highest in mature iGLUTs when looking across all NDD gene perturbations. **(C,D)** Transcriptomic convergence is greater between KOs with stronger associations with ASD compared to DD across all cell-types. Associations between the ASD risk specificity and convergence was performed by calculating the mean ASD posterior probability (p.p.) from Fu et al. 2022 across KOs in a set; KO sets were stratified into three groups based on these scores (DD = mean ASD p.p. <=0.1; ASD = mean ASD p.p. >=0.9; mid= mean ASD p.p. <=0.55 & >=0.45) and differences in gene-level **(C)** and network-level **(D)** convergence tested using non-parametric Kruskal-Wallis test, with pairwise comparisons performed with Wilcoxon's test. For network-level convergence in iNPCs, comparisons between DD and ASD sets were not possible as none of the DD KO sets resolved convergent networks –an observation further suggesting that DD sets are less convergent. **(E,F)** Associations between ASD risk specificity and convergence by cell-type and the number of KOs in a set as assessed through linear correlation (Pearsons Correlation Coefficient (PCC) or "R"). **(E)** ASD p.p was significantly positively associated with gene-level convergence in mature neurons, especially iGABAs, but this was influenced by the number of KOs in a set. For example, gene-level convergence across 5 KOs was significantly positively correlated with ASD specificity in **(E, ii)** iGABAs

(R=0.43, p<2.2e-16) but not in **(E, iii)** immature iGLUTs (R=0.017, p=0.44). Likewise, network-level convergence in sets of 5 KOs was positively correlated with ASD specificity in **(F, i-ii)** iGABAs (R=0.28, p=0.0016) but not immature iGLUTs (R=-0.13, p=0.32). The lack of clear correlation patterns suggest that while mean convergence is higher across genes with stronger ASD associations **(C,D),** this relationship is not linear and likely influenced by other shared factors between risk genes.

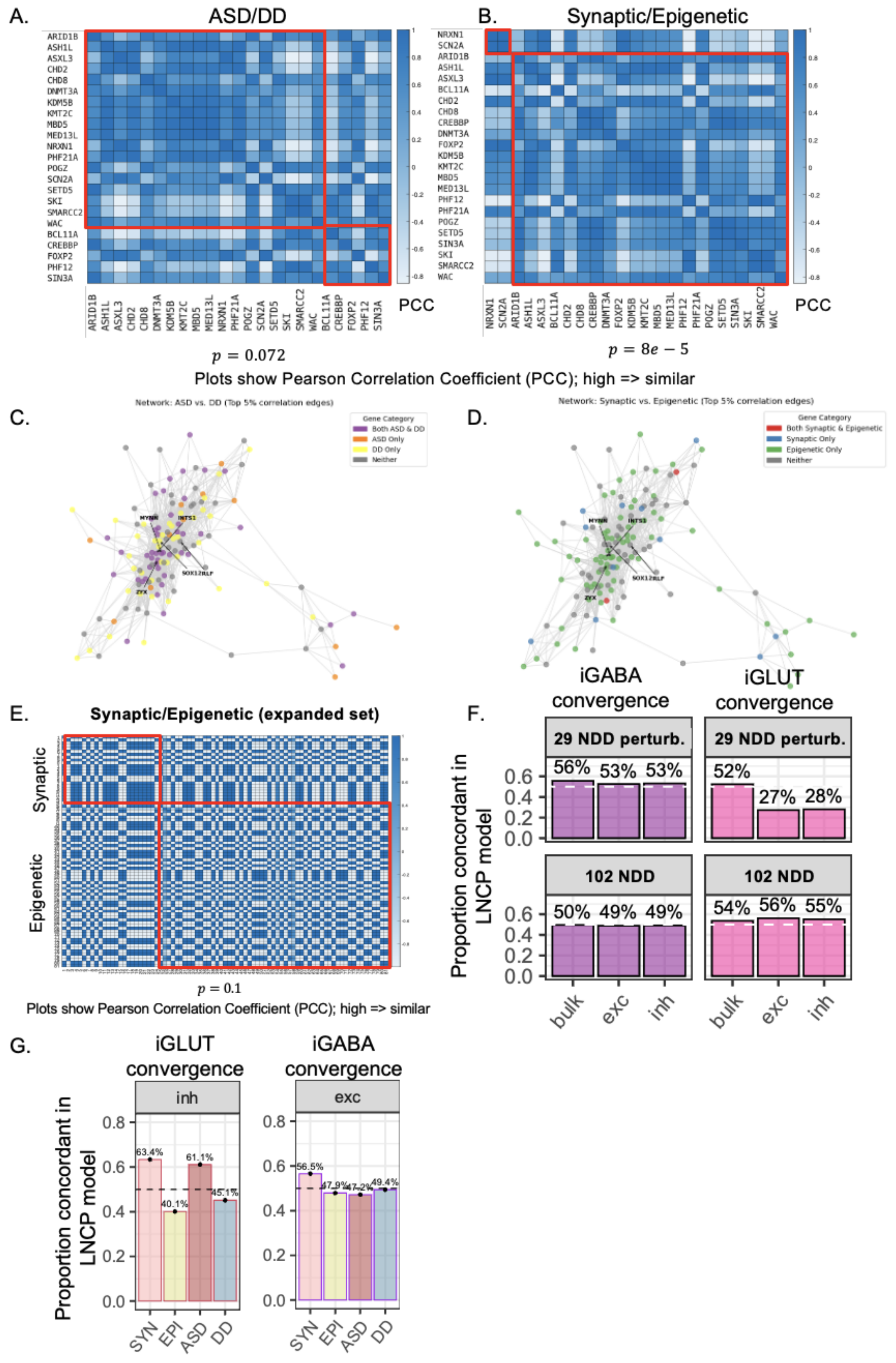

A.
ASD/DD
ARID1B
ASH1L
ASXL3
CHD2
CHD8
DNMT3A
KDM5B
KMT2C
MBD5
MED13L
NRXN1
PHF21A
POGZ
SCN2A
SETD5
SKI
SMARCC2
WAC
BCL11A
CREBBP
FOXP2
PHF12
SIN3A
PCC
$p = 0.072$
B.
Synaptic/Epigenetic
NRXN1
SCN2A
ARID1B
ASH1L
ASXL3
BCL11A
CHD2
CHD8
CREBBP
DNMT3A
FOXP2
KDM5B
KMT2C
MBD5
MED13L
PHF12
PHF21A
POGZ
SETD5
SIN3A
SKI
SMARCC2
WAC
PCC
$p = 8e - 5$
Plots show Pearson Correlation Coefficient (PCC); high => similar
C.
Network: ASD vs. DD (Top 5% correlation edges)
Gene Category
Both ASD & DD
ASD Only
DD Only
Neither
MYNN
INTS1
SOX12RLF
ZYX
D.
Network: Synaptic vs. Epigenetic (Top 5% correlation edges)
Gene Category
Both Synaptic & Epigenetic
Synaptic Only
Epigenetic Only
Neither
MYNN
INTS1
SOX12RLF
ZYX
E.
Synaptic/Epigenetic (expanded set)
Synaptic
Epigenetic
$p = 0.1$
Plots show Pearson Correlation Coefficient (PCC); high => similar
F.
iGABA convergence
iGLUT convergence
29 NDD perturb.
29 NDD perturb.
56% 53% 53%
52% 27% 28%
102 NDD
102 NDD
50% 49% 49%
54% 56% 55%
Proportion concordant in LNCP model
0.6
0.4
0.2
0.0
bulk
exc
inh
bulk
exc
inh
G.
iGLUT convergence
iGABA convergence
inh
exc
Proportion concordant in LNCP model
0.8
0.6
0.4
0.2
0.0
63.4%
40.1%
61.1%
45.1%
56.5%
47.9%
47.2%
49.4%
SYN
EPI
ASD
DD
SYN
EPI
ASD
DD

SI Figure 17. Further analysis of LNCTP convergent genes. Predicted *in silico* perturbation effects were significantly more correlated within categories defined by **(A)** disorder association (ASD – DD; p=0.072) and **(B)** function (synaptic – epigenetic; p=8x10-5). Heatmaps show the correlations of the LNCTP predicted *in silico* log fold-changes across PsychENCODE individuals for ASD/DD and Synaptic/Epigenetic CRISPR perturbations respectively (p-values, 1-tailed t-test for decreased correlation between perturbation categories). **(C,D)** Network visualization of the proximity of functional classes of *in silico* convergent genes, where adjacency represents co-expression of bulk expression across PsychENCODE subjects. Colored by disorder association **(C)** or functional association **(D)**. **(E)** Repeat of analysis in (A,B) with the expanding list of 102 NDD perturbations (p=0.1). **(F)** Repeat of the analysis in Fig. 5B, using all 102 NDD perturbations. **(G)** Repeat of the analysis in Fig. 5C, using all 102 NDD perturbations showing concordance between *in silico* convergence in inhibitory neurons with convergence in iGLUTs and *in silico* convergence in excitatory neurons with convergence in iGABAs.

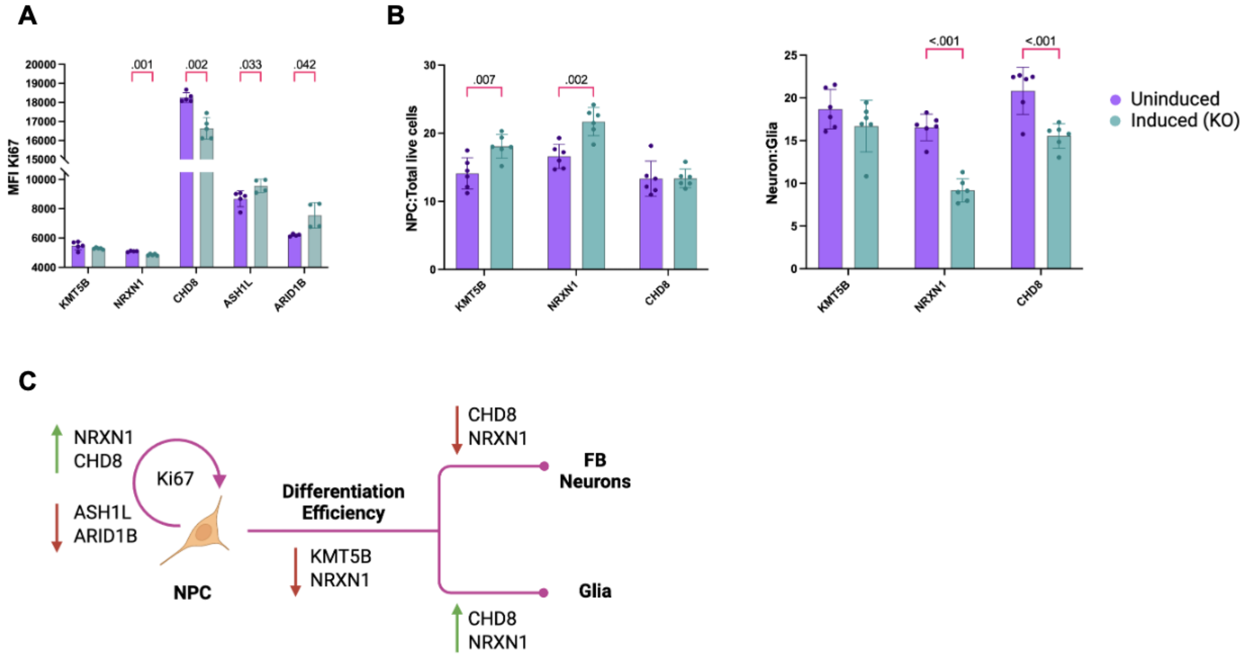

SI Figure 18. Impact of NDD KO on proliferation and neurogenesis in NPCs and neurons. (**A**) Proliferation assessment of NPCs using Ki-67 staining. NPCs were transduced with a pooled gRNA library containing four gRNAs per target gene targeting selected NDD genes (*KMT5B, NRXN1, CHD8, ASH1L, ARID1B*) and analyzed 7 days after Cas9 activation with doxycycline (dox). Flow cytometry analysis revealed significant changes in the median fluorescence intensity (MFI) of Ki-67-FITC. (**B**) Neurogenesis and gliogenesis assessment in differentiated forebrain cultures. NPCs were differentiated into neurons and glia, and the ratio of NPCs to total live cells and neurons to glia was quantified by flow cytometry. Error bars represent standard deviation (SD). Each dot represents an independent replicate. Unpaired t-test with Welch correction; p-values corrected for multiple comparisons using FDR. (**C**) Summary schematic of the findings. CHD8 and NRXN1 KO promote gliogenesis and NPC proliferation, while ASH1L and ARID1B KO reduce NPC proliferation. KMT5B KO reduces differentiation efficiency but does not significantly alter neuron-to-glia ratios. Created in BioRender. Pmnd, P. (2026) https://BioRender.com/zb0nt5t.

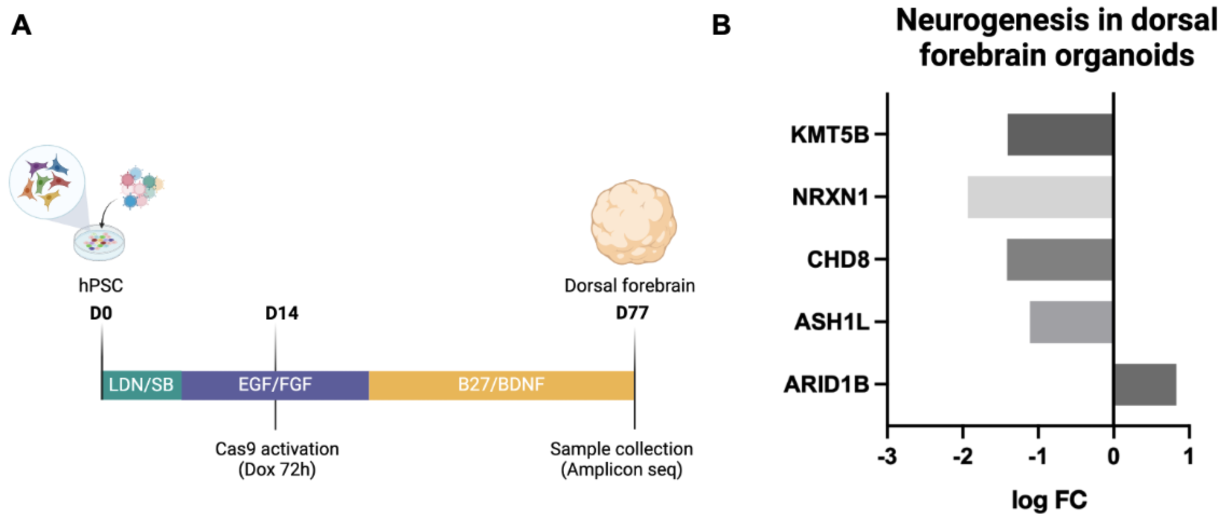

SI Figure 19. Pooled CRISPR screen to uncover the NDD KO effects on cortical organoid development. (A) Schematic representation of the experimental workflow. H1-hESC-iCas9 cells were transduced with a pooled gRNA library targeting five NDD genes (*KMT5B, NRXN1, CHD8, ASH1L, ARID1B*) and 20% non-targeting controls. Cas9 was activated on Day 14 via doxycycline (2 µg/mL) for 72 hours and maintained in culture for ~80 days. Organoids were subsequently processed for gRNA enrichment analysis using PCR amplicon sequencing to assess the relative abundance of gRNAs targeting each gene. Created in BioRender. Pmnd, P. (2026) https://BioRender.com/zb0nt5t. **(B**) Bar plot showing the log fold change (logFC) of gRNA abundance relative to non-targeting controls. Negative logFC values indicate depletion of gRNAs targeting genes associated with reduced neurogenesis or cell survival (e.g., CHD8 and KMT5B), while positive logFC values (e.g., ARID1B) indicate enhanced proliferation or survival. These results demonstrate distinct roles for NDD genes in regulating organoid developmental dynamics, with ARID1B associated with increased proliferation and KMT5B and CHD8 contributing to neuronal depletion.

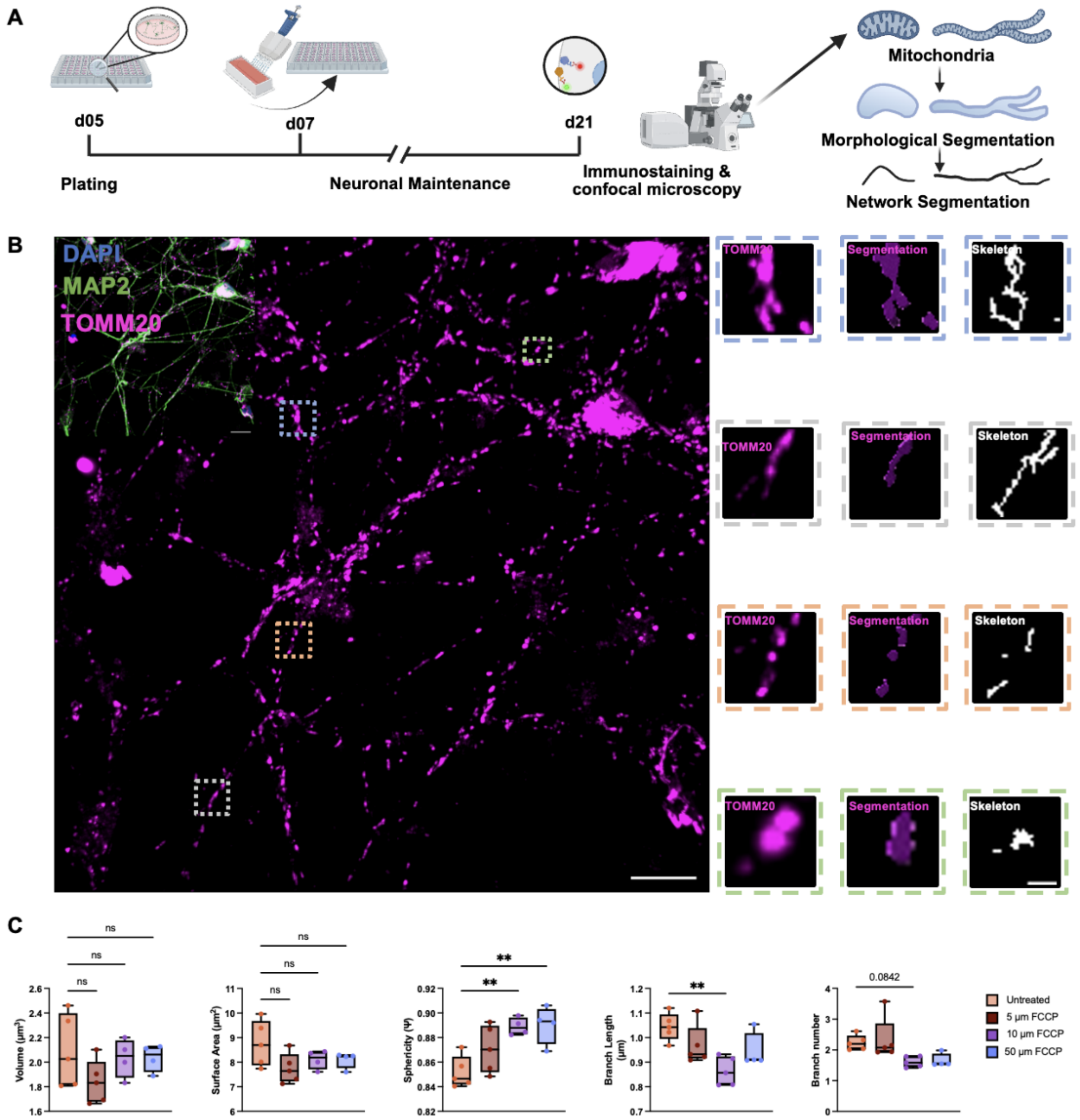

SI Figure 20. High resolution microscopy captures diversity of mitochondrial morphology in iGLUTs and is sensitive to mitochondrial pharmacological insults. A) Timeline of culture and microscopy experiments **B)** Representative image of iGLUTs labelled with mitochondria (TOMM20, magenta). Inset: merged representative image of iGLUTs with MAP2 (green) and DAPI (blue). Representative images of networked (blue), fused(grey), fragmented (yellow) and swollen mitochondria (green). Mitochondria are volumetrically segmented and skeletonized for morphological and network analysis. Scale 10 µm, 1 µm**. C)** Microscopy pipeline is sensitive, capturing dose-dependent mitochondrial fragmentation following FCCP treatment. FCCP treatment causes dose-dependent mitochondrial fragmentation (decreases in mitochondrial sphericity and

increases in branch length) independent of changes in mitochondrial volume and surface area.

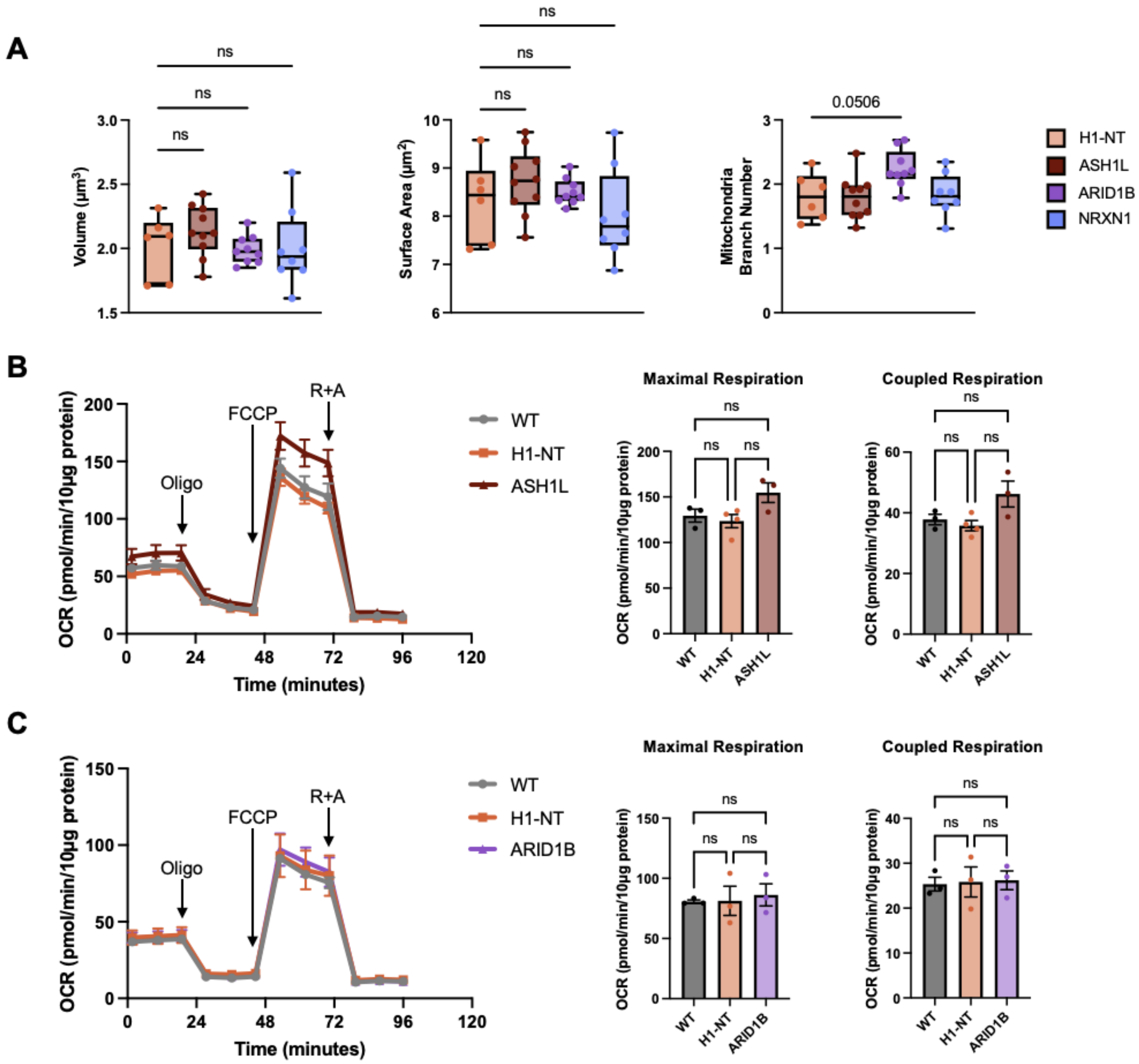

SI Figure 21. NDD KOs converge on mitochondrial function differently in mature iGLUTs. (A) High resolution, high-throughput microscopy of mitochondrial morphology of segmented MAP2-positive dendrites labelled for TOMM20-positive mitochondria. Boxplots indicate minimum, maximum and mean values. Each datapoint indicates one well of a 96-well, representing hundreds of µm^2 of neuronal area and tens of thousands of individual mitochondria (*adjusted $p<0.05$, ** adjusted $p<0.01$). **(B–C)** Seahorse assay results for maximal and coupled respiration in ASH1L (B) and ARID1B (C) KO iGLUTs. Oligo: oligomycin; FCCP: carbonyl cyanide 4-(trifluoromethoxy) phenylhydrazone; R+A: rotenone and antimycin A. Data are presented as mean ± SEM. Statistical analysis was performed using one-way ANOVA. Each datapoint represents one well of a 24-well Seahorse assay plate. The experiment was independently replicated twice.

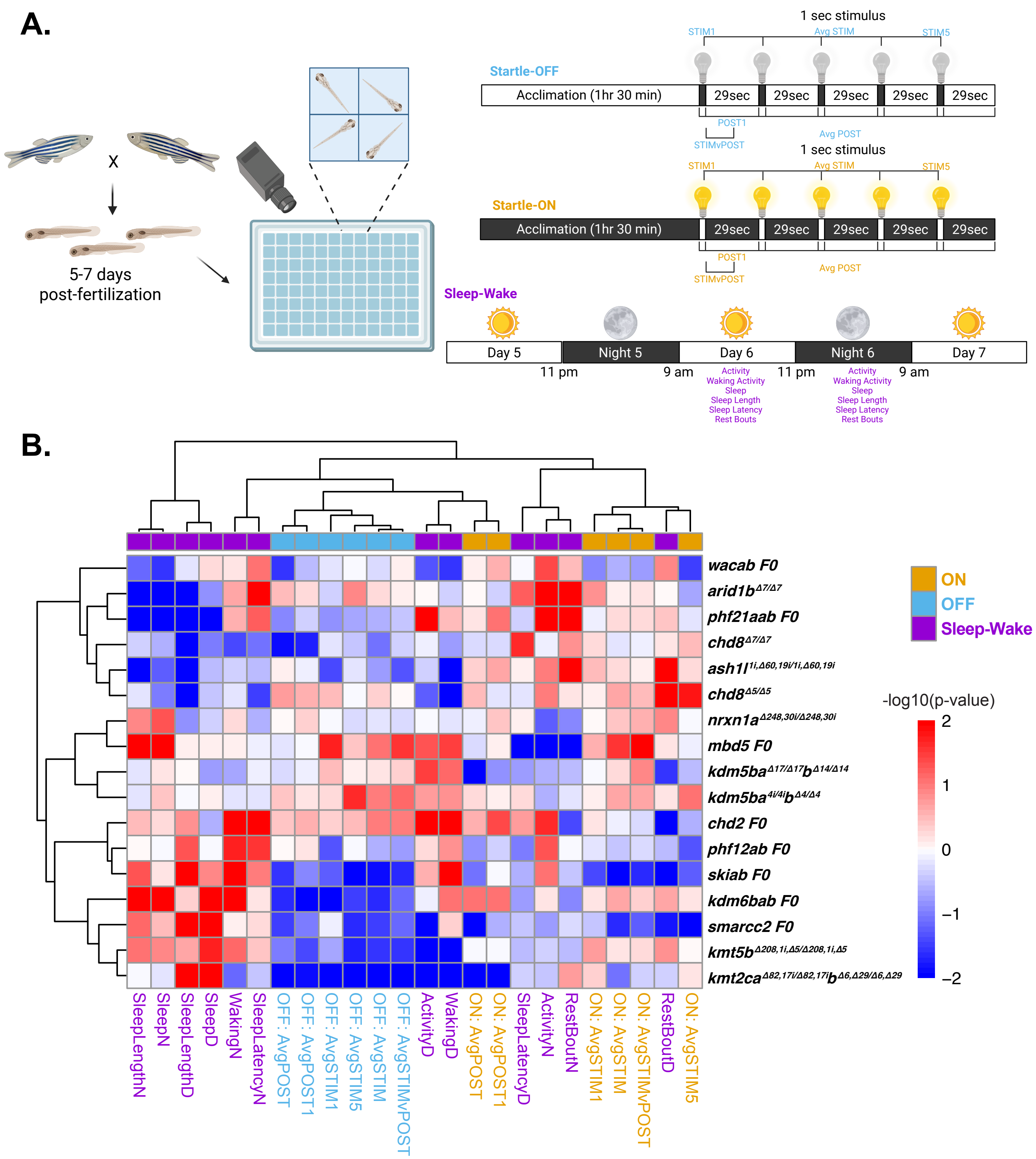

SI Figure 22. Phenotypic clustering of 15 NDD genes in mutant zebrafish. (A) Experimental setup and behavioral paradigms for visual-startle and sleep-wake assays. Larval zebrafish mutants are assessed for basic arousal and sensorimotor behaviors between 5-7 days post-fertilization (dpf). Individual larvae are placed in a 96-well plate and behavior is tracked with a video camera within a Zebrabox. *Visual-startle*: Larvae acclimate to ambient light or darkness and are exposed to five 1-second flashes of lights-OFF or lights-ON stimulus at 29-second intervals at 5 dpf. *Sleep-wake:* Larvae are

exposed to 14h:10h light:dark cycle in which movement, sleep, and activity parameters are measured over 5-7 dpf. **(B)** Hierarchical clustering of mutant behavioral fingerprints across startle-OFF (light blue), startle-ON (orange) and sleep-wake (purple) parameters. Each box represents the signed -$\log_{10}$-transformed p-values from linear mixed models (LMM) comparing behaviors in stable mutant and background-matched wild-type fish and F0 mutant and scrambled control (red, increased in mutant; blue decreased in mutant).

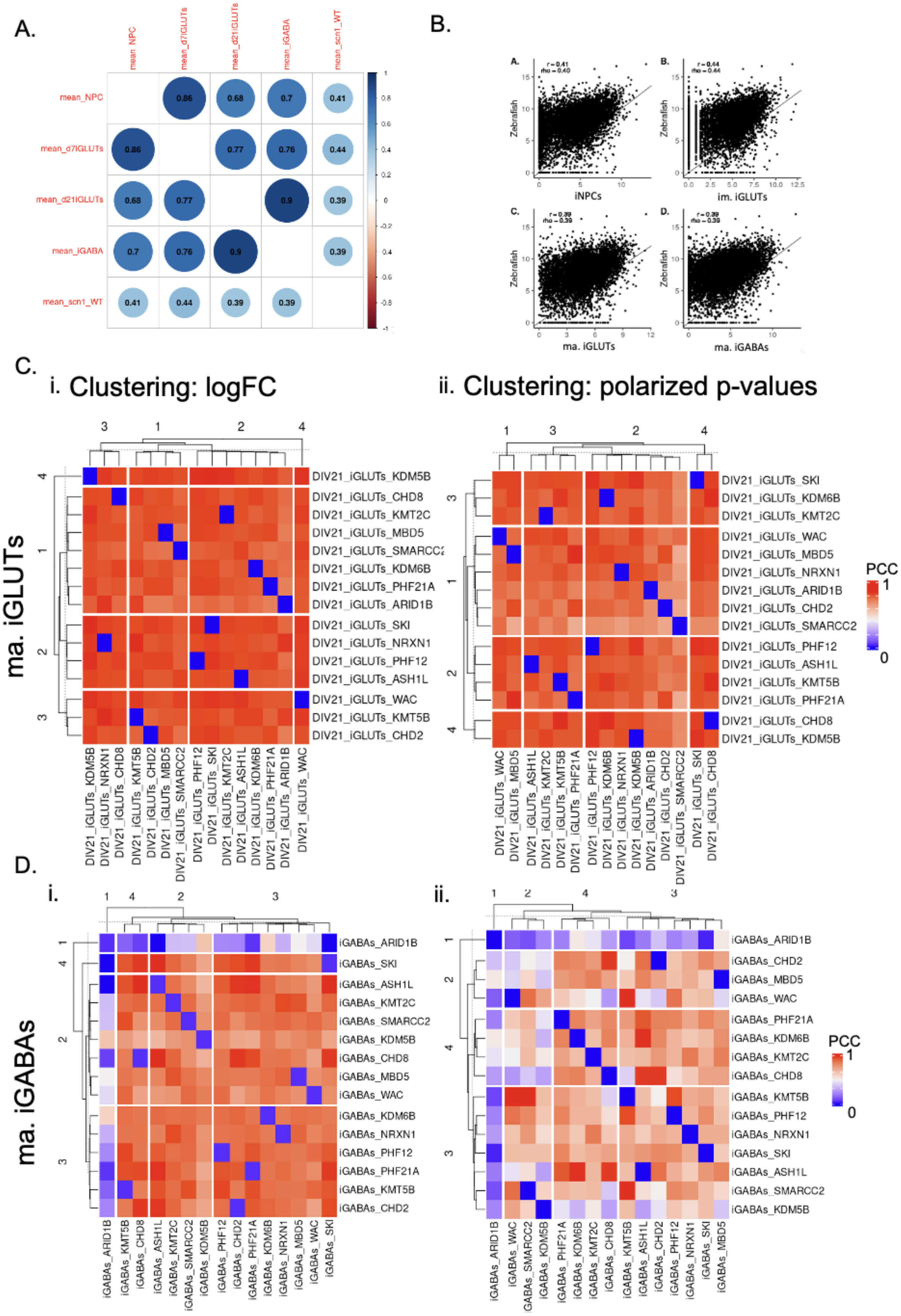

A.
mean_NPC
mean_d7iGLUTs
mean_d21iGLUTs
mean_iGABA
mean_scn1_WT
0.86
0.68
0.7
0.41
0.77
0.76
0.44
0.9
0.39
B.
Zebrafish
iNPCs
im. iGLUTs
ma. iGLUTs
ma. iGABAs
C.
i. Clustering: logFC
ii. Clustering: polarized p-values
ma. iGLUTs
DIV21_iGLUTs_KDM5B
DIV21_iGLUTs_CHD8
DIV21_iGLUTs_KMT2C
DIV21_iGLUTs_MBD5
DIV21_iGLUTs_SMARCC2
DIV21_iGLUTs_KDM6B
DIV21_iGLUTs_PHF21A
DIV21_iGLUTs_ARID1B
DIV21_iGLUTs_SKI
DIV21_iGLUTs_NRXN1
DIV21_iGLUTs_PHF12
DIV21_iGLUTs_ASH1L
DIV21_iGLUTs_WAC
DIV21_iGLUTs_KMT5B
DIV21_iGLUTs_CHD2
PCC
1
0
D.
i.
ii.
ma. iGABAs
iGABAs_ARID1B
iGABAs_SKI
iGABAs_ASH1L
iGABAs_KMT2C
iGABAs_SMARCC2
iGABAs_KDM5B
iGABAs_CHD8
iGABAs_MBD5
iGABAs_WAC
iGABAs_KDM6B
iGABAs_NRXN1
iGABAs_PHF12
iGABAs_PHF21A
iGABAs_KMT5B
iGABAs_CHD2

SI Figure 23. Transcriptomic clustering of 15 NDD genes in mature neurons with paired behavioral data in mutant zebrafish. (A-B) Two-tailed Pearson's correlations of expression of non-targeting cells in iNPCs, iGLUTs, and iGABAs significantly correlates with expression of gene homologs and orthologs in the wild-type zebrafish brain. **(C-D)** Heatmap of KO two-tailed Pearson's correlations based on logFC **(i)** and polarized –log10 pvalues **(ii)** in mature iGLUTs **(C)** and iGABAs **(D).** Differential gene expression results from individual KOs were filtered for genes with direct zebrafish gene homologs and orthologs using the R package orthogene. Pearson's correlations across logFC and polarized pvalues (-log10 pvalues multiple by -1 if logFC was below 0) were used to subset KOs into 4 K-means clusters for each cell-type.

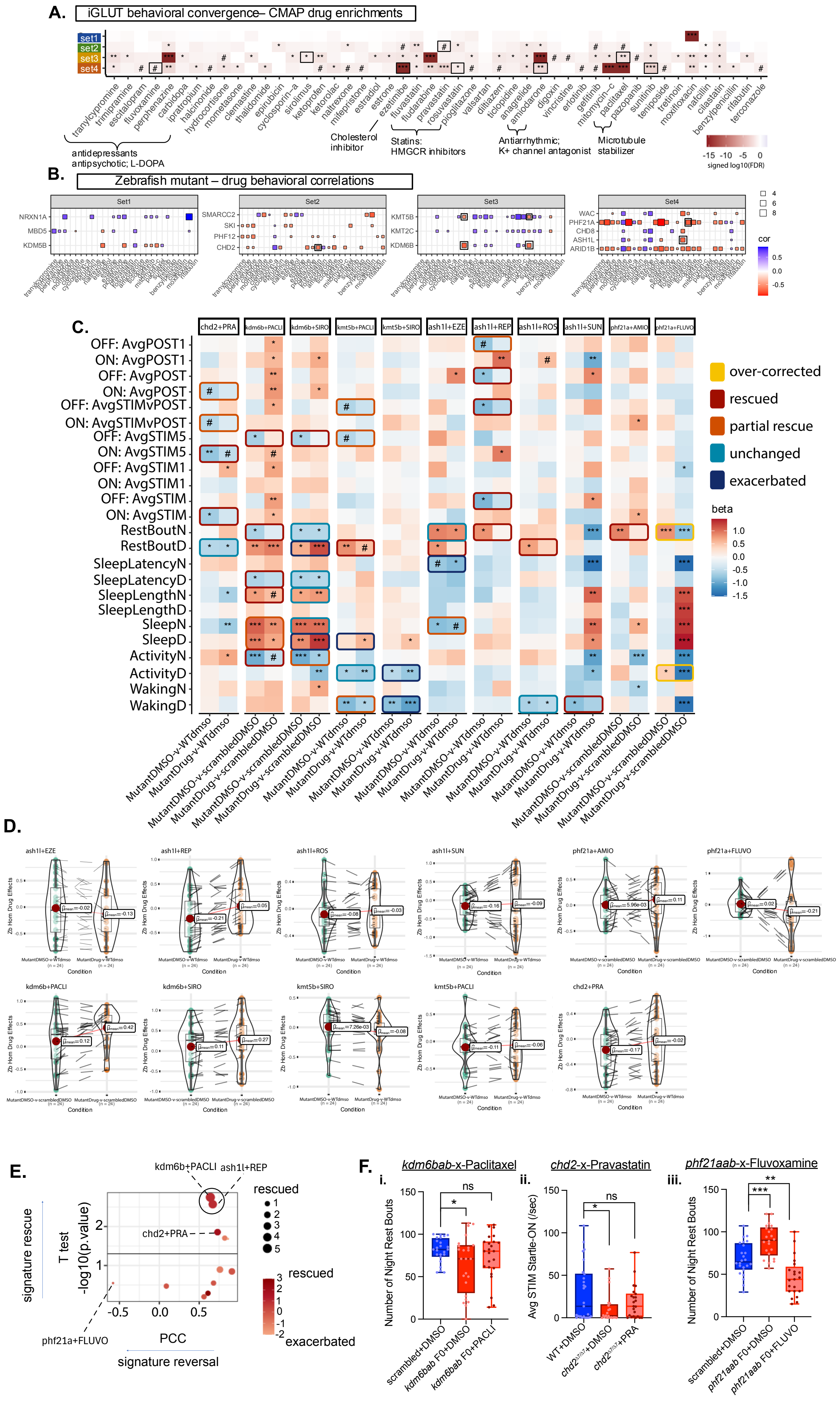
A. iGLUT behavioral convergence– CMAP drug enrichments
set1
set2
set3
set4
antidepressants
antipsychotic; L-DOPA
Cholesterol inhibitor
Statins: HMGCR inhibitors
Antiarrhythmic; K+ channel antagonist
Microtubule stabilizer
signed log10(FDR)
B. Zebrafish mutant – drug behavioral correlations
Set1
Set2
Set3
Set4
cor
C.
over-corrected
rescued
partial rescue
unchanged
exacerbated
beta
D.
E.
kdm6b+PACLI
ash1l+REP
chd2+PRA
phf21a+FLUVO
signature rescue
T test -log10(p.value)
PCC
signature reversal
rescued
exacerbated
F.
kdm6bab-x-Paclitaxel
chd2-x-Pravastatin
phf21aab-x-Fluvoxamine
Number of Night Rest Bouts
Avg STIM Startle-ON (/sec)
scrambled+DMSO
kdm6bab F0+DMSO
kdm6bab F0+PACLI
WT+DMSO
phf21aab F0+DMSO
phf21aab F0+FLUVO

SI Figure 24. Drug reversal of behavioral phenotypes in mutant zebrafish. **(A)** Drug enrichments using cMAP were calculated for cell-type specific convergence across sets and filtered for drugs included in a screen of 376 compounds for behavioral effects in zebrafish**.** Prediction of drugs targeting these convergent signals in mature neurons identify drug reversers that target shared convergence across the sets (e.g., antipsychotic perphenazine in iGLUTs) and some specific to unique convergence (e.g., valsartan only in Set 3 iGABAs: **SI Data 2**). The heatmap is colored by the polarized (- or + based on direction of enrichment) unadjusted p-values. Significance after multiple testing corrections is indicated by the following: #nominal p-value<0.05, *FDR<0.05, **FDR<0.01, *FDR<0.001). Notably phenotypic and drug enrichments were only FDR significant in iGLUTs, reflecting that convergent signatures in mature iGLUTs across NDD sets are especially relevant for downstream phenotypes. **(B)** For top predicted drugs from convergence analysis in mature iGLUTs, wild-type zebrafish behavior following exposure to the drug was compared to behavior in individual NDD mutants. **(C)** Heatmap of beta values for each tested behavior between the effect of the mutant alone (homozygous mutant or crispant+DMSO-v-wild-type or scrambled+DMSO) and for the effect of the interaction of the mutant with drug treatment compared to WT vehicle (homozygous mutant or crispant+drug-v-wild-type or scrambled+DMSO). **(D)** Comparison of the magnitude of effect (beta) on behavior between the mutant compared to Mutant+Drug groups for each empirical test. In the boxplots, the median is represent by the line (centre) and the mean as the red point. The lower and upper hinges correspond to the first and third quartiles (the 25th and 75th percentiles). The upper and lower whiskers extend from the hinge to the largest or smallest value up to 1.5 * IQR (inter-quartile range). All data points are plotted individually. **(E)** Visualization of mutant-x-drug tests, comparing the extent of behavioral signature rescue to signature reversal. **(F)** Representative parameters that were either rescued (i-ii) or over-corrected (iii) in MutantxDrug combinations. (i) Decreased nighttime sleep bouts in *kdm6b* mutants were rescued by paclitaxel. (Unpaired t test with Welch's correction: crispant+DMSO vs scrambled+DMSO p=0.0289, t=2.291, df=31.12; crispant+PACLI vs scrambled+DMSO p=0.2485) (ii) Decreased response to lights-ON stimulus in *chd2* mutants was rescued by pravastatin. (Unpaired t test with Welch's correction: $chd2^{\Delta7/\Delta7}$+DMSO vs WT+DMSO p=0.0206, t=2.409, df=40.47; $chd2^{\Delta7/\Delta7}$+PRA vs WT+DMSO p=0.1909) (iii) Nighttime sleep bouts were increased in *phf21a* mutants but significantly decreased beyond wildtype levels with fluvoxamine (Unpaired t test with Welch's correction crispant+DMSO vs scrambled+DMSO p=0.0004, t=3.808, df=43.86; crispant+FLUVO vs scrambled+DMSO p=0.0016, t=3.392, df=39.59).
