# Supplementary material for: Transcriptomic and phenotypic convergence of neurodevelopmental disorder risk genes in vitro and in vivo
Source: Nat Neurosci. 2026 Apr 24;29(5):1079–94. doi: 10.1038/s41593-026-02247-7 (PMC13156037; doi:10.1038/s41593-026-02247-7)
Supplement: Supplementary file 2 — Reporting Summary [file 41593_2026_2247_MOESM2_ESM.pdf]

nature portfolio

Corresponding author(s): Kristen Brennand

Last updated by author(s): 02/04/2026

Reporting Summary

Nature Portfolio wishes to improve the reproducibility of the work that we publish. This form provides structure for consistency and transparency in reporting. For further information on Nature Portfolio policies, see our Editorial Policies and the Editorial Policy Checklist.

Statistics

For all statistical analyses, confirm that the following items are present in the figure legend, table legend, main text, or Methods section.

n/a	Confirmed	
☐	☒	The exact sample size (*n*) for each experimental group/condition, given as a discrete number and unit of measurement
☐	☒	A statement on whether measurements were taken from distinct samples or whether the same sample was measured repeatedly
☐	☒	The statistical test(s) used AND whether they are one- or two-sided<br>*Only common tests should be described solely by name; describe more complex techniques in the Methods section.*
☐	☒	A description of all covariates tested
☐	☒	A description of any assumptions or corrections, such as tests of normality and adjustment for multiple comparisons
☐	☒	A full description of the statistical parameters including central tendency (e.g. means) or other basic estimates (e.g. regression coefficient) AND variation (e.g. standard deviation) or associated estimates of uncertainty (e.g. confidence intervals)
☐	☒	For null hypothesis testing, the test statistic (e.g. *F*, *t*, *r*) with confidence intervals, effect sizes, degrees of freedom and *P* value noted<br>*Give P values as exact values whenever suitable.*
☐	☒	For Bayesian analysis, information on the choice of priors and Markov chain Monte Carlo settings
☐	☒	For hierarchical and complex designs, identification of the appropriate level for tests and full reporting of outcomes
☐	☒	Estimates of effect sizes (e.g. Cohen's *d*, Pearson's *r*), indicating how they were calculated

Our web collection on statistics for biologists contains articles on many of the points above.

Software and code

Policy information about availability of computer code

Data collection

single cell cDNA and gRNA sequencing was collected from cells were loaded into 10X in 4 lanes per cell type, targeting 20,000 cells per lane for a total of ~80,000 targeted cells per cell type. scRNA-seq was performed at Yale Genomics Core with the 10X single cell 5' v2 HT with CRISPR barcode kit

Automated, high-throughput, quantitative behavioral profiling of larval zebrafish measured arousal and sensorimotor processing as a readout of circuit-level deficits resulting from gene perturbation and/or drug exposure. Individual larvae were added to each well of a 96 well plate containing 650µl of standard embryo water and, for drug exposure experiments, a 5mM stock solution of the drug of interest was pipetted directly into each well at a final concentration of 10 µM (0.1% DMSO final concentration). We quantified 24 visual-startle and sleep-wake parameters. Visual-startle OFF and Visual-startle ON assays measured 6 parameters each: (i) average intensity of all startle responses; (ii) average post-stimulus activity; (iii) average activity after first stimulus; (iv) stimulus versus post-stimulus activity; (v) intensity of responses to the first stimulus; (vi) intensity of responses to the final stimulus. Sleep-wake assays measured 6 parameters in daytime and nighttime: : (i) total activity; (ii) total sleep; (iii) waking activity; (iv) rest bouts; (v) sleep length; (vi) sleep latency.

Data analysis

Detailed data analysis is outlined in the methods; full code for the analysis workflow will be uploaded to Synapse (syn72039767) and made publicly available prior to publication.

All RNA-sequencing FASTQ files underwent matching analytical procedures. In brief, mRNA sequencing reads were mapped to the GRCh38 reference genome using the Cellranger Software. To generate count matrices for HTO and GDO libraries, the kallisto indexing and tag extraction (kite) workflow were used. Count matrices were used as input into the R/Seurat package to perform downstream analyses, including QC, normalization, cell clustering, and gRNA and HTO/GDO demultiplexing. Data was transformed into pseudobulked samples (by

lane) land transformed into og2CPM using VOOM (Limma), tested for differential expression using EdgeR and Limma, and co-expression networks resolved by Bayesian bi-clustering using BicMix.

The full analysis pipeline (including code and processed data objects) used for analysis of single-cell CRISPR-KO data, evaluation and characterization of gene-level and network level convergence, and predictive modeling using random forest are publicly available through Synppase (syn72039767).
Custom MATLAB software developed by the Hoffman Lab to analyze visual-startle response parameters is available on github at https://github.com/ehoffmanlab/Weinschutz-Mendes-et-al-2023-behavior; https://doi.org/10.5281/zenodo.7644898. Custom MATLAB sofyware developed by Jason Rihel to analyze sleep-wake assays is available on github at https://github.com/JRihel/Sleep-Analysis/tree/ Sleep-Analysis-Code; https://doi.org/10.5281/zenodo.7644073.

kallisto/0.46.1
bustools/0.40.0
kb_python/0.26.4
python/ 2.x and 3.x
cellRanger/v7.2
Metal/version release 2018-08-28
MAGMA/v1.10
FUMA/v1.5.2
WebGestalt/version release 2024
cMAP Query Tool and LINCs 2020 updated 11/23/2021

R version 4.1.0-4.2.0

R packages:
Seurat v5.1.0 was used for all analysis of single-cell CRISPR screens prior to pseudo-bulking.

```
 [1] MASS_7.3-60               randomForest_4.7-1.1
 [3] GGally_2.1.2              ggExtra_0.10.0
 [5] ggstatsplot_0.9.4         WebGestaltR_0.4.6
 [7] factoextra_1.0.7          lsr_0.5.2
 [9] GOSemSim_2.22.0           DOSE_3.22.0
[11] enrichplot_1.16.1         GeneNet_1.2.16
[13] fdrtool_1.2.17            longitudinal_1.1.13
[15] corpcor_1.6.10            clusterProfiler_4.4.4
[17] org.Hs.eg.db_3.15.0       AnnotationDbi_1.58.0
[19] ggpubr_0.4.0              venn_1.11
[21] ggrepel_0.9.1             scuttle_1.6.2
[23] psych_2.2.5               statmod_1.4.36
[25] Glimma_2.6.0              pamr_1.56.1
[27] survival_3.5-7            cluster_2.1.4
[29] sva_3.44.0                genefilter_1.78.0
[31] mgcv_1.8-40               nlme_3.1-157
[33] RColorBrewer_1.1-3        corrplot_0.92
[35] doParallel_1.0.17         iterators_1.0.14
[37] foreach_1.5.2             variancePartition_1.26.0
[39] BiocParallel_1.32.6       forcats_1.0.0
[41] stringr_1.5.1             purrr_1.0.1
[43] readr_2.1.2               tidyr_1.2.0
[45] tibble_3.2.1              tidyverse_1.3.1
[47] cowplot_1.1.1             presto_1.0.0
[49] data.table_1.14.2         Rcpp_1.0.10
[51] edgeR_3.38.1              limma_3.54.2
[53] ggpointdensity_0.1.0      DropletUtils_1.18.1
[55] SingleCellExperiment_1.18.1 SummarizedExperiment_1.28.0
[57] Biobase_2.56.0            GenomicRanges_1.48.0
[59] GenomeInfoDb_1.34.9       IRanges_2.32.0
[61] S4Vectors_0.36.2          BiocGenerics_0.44.0
[63] MatrixGenerics_1.10.0     matrixStats_0.62.0
[65] glmGamPoi_1.10.2          Matrix_1.6-4
[67] BUSpaRse_1.16.0           reshape2_1.4.4
[69] scales_1.3.0              sctransform_0.4.1
[71] dplyr_1.1.2               viridis_0.6.2
[73] viridisLite_0.4.2         patchwork_1.2.0.9000
[75] ggplot2_3.5.0.9000        devtools_2.4.3
[77] usethis_2.1.6             Seurat_5.0.1
[79] SeuratObject_5.0.1        sp_1.5-0
[81] BicMix
```

For zebrafish behavioral assays: Custom MATLAB code is available at https://github.com/ehoffmanlab/Weinschutz-Mendes-et-al-2023-behavior and https://github.com/JRihel/Sleep-Analysis/tree/Sleep-Analysis-Code; DOI: 10.5281/zenodo.7644073.

For manuscripts utilizing custom algorithms or software that are central to the research but not yet described in published literature, software must be made available to editors and reviewers. We strongly encourage code deposition in a community repository (e.g. GitHub). See the Nature Portfolio guidelines for submitting code & software for further information.

Data

Policy information about availability of data

All manuscripts must include a data availability statement. This statement should provide the following information, where applicable:

- Accession codes, unique identifiers, or web links for publicly available datasets
- A description of any restrictions on data availability
- For clinical datasets or third party data, please ensure that the statement adheres to our policy

All source donor hiPSCs have been deposited at the Rutgers University Cell and DNA Repository (study 160; http://www.nimhstemcells.org/).
sc-RNA sequencing data reported in this paper is available on Gene Expression Omnibus (GSE319096). Previously published SCZ-CRISPRa screen datasets that were used for external validation of random forest models are available on the GEO (GSE200774) and on Synapse (syn27819129). Raw data for the main figures, as well as secondary summary statistics from main analysis are available through Synapse (syn72039767)

Research involving human participants, their data, or biological material

Policy information about studies with human participants or human data. See also policy information about sex, gender (identity/presentation), and sexual orientation and race, ethnicity and racism.

Reporting on sex and gender	Two human-induced pluripotent stem cell-lines were used in this study - one XX and one XY karyotipic sex. However, this study is not powered to make any claims related to sex differences.
Reporting on race, ethnicity, or other socially relevant groupings	NA
Population characteristics	hiPSCs (human-induvded pluripotent stem cells) were derived from an XX and XY donor, representing neurotypical controls with no history of psychiatric disorders and who are of European ancestry.
Recruitment	NA. Recruitment of donors was not a part of this study. All lines were selected from a previously reported case/control hiPSC cohort of childhood onset SZ (COS) (Hoffman et al, Nat Comm 2017)
Ethics oversight	Yale University Institutional Review Board waived ethical approval for this work. Ethical approval was not required because the hiPSC lines, lacking association with any identifying information and widely accessible from a public repository, are thus not considered to be human subjects research. Post-mortem data are similarly lacking identifiable information and are not considered human subjects research.

Note that full information on the approval of the study protocol must also be provided in the manuscript.

Field-specific reporting

Please select the one below that is the best fit for your research. If you are not sure, read the appropriate sections before making your selection.

☒ Life sciences ☐ Behavioural & social sciences ☐ Ecological, evolutionary & environmental sciences

For a reference copy of the document with all sections, see nature.com/documents/nr-reporting-summary-flat.pdf

Life sciences study design

All studies must disclose on these points even when the disclosure is negative.

Sample size

For each KO target 3-4 gRNAs were used. The number of cells for each scCRISPR screen in each celltype was 20,000 cells per lane for a total of ~80,000 targeted cells. Following demultiplexing and QC a minimum of 75 cells was necessary for analysis with an average of 474 cells per sgRNA - single-cell data was pseudobulked across Lanes to establish 4 samples per gene KO used in pseudobulk differential gene expression analysis.

For zebrafish baseline behavioral screening, sample sizes are as follows: kmt5b WT n=62, HOM n=71; mbd5 scrambled n=39, crispant n=56; kdm5b 4i:del4 WT n=42, HOM n=55; kdm5b del17:del14 n=68, HOM n=69; phf12ab scrambled n=32, crispant n=15; skiab scrambled n=45, crispant n=33; chd2 scrambled n=43, crispant n=40; smarcc2 scrambled n=36, crispant n=35; kdm6bab scrambled n=40, crispant n=34; kmt2cab WT n=38, HOM n=20; wacab scrambled n=32, crispant n=32; arid1b WT n=81, HOM n=81; phf21a scrambled n=52, crispant n=44; chd8 del7 WT n=180, HOM n=164; chd8 del5 WT n=143, HOM n=167; ash1l WT n=19, HOM n=23; nrxn1a WT n=24 HOM n=28.

For zebrafish gene-x-drug experiments, sample sizes are as follows: phf21a + amiodarone scrambled DMSO n=24, F0 DMSO n=22, F0 AMIO n=23; phf21a + fluvoxamine scrambled DMSO n=24, F0 DMSO n=22, F0 FLUVO n=21; chd2 + pravastatin WT DMSO n=27, HOM DMSO n=21, HOM PRAVA n=23; kdm6b + paclitaxel scrambled DMSO n=24, F0 DMSO n=24, F0 PACLI n=24; kdm6b + sirolimus scrambled DMSO n=24, F0 DMSO n=24, F0 SIRO n=23; kmt5b + paclitaxel WT DMSO n=20, HOM DMSO n=22, HOM PACLI n=26; kmt5b + sirolimus WT DMSO n=27, HOM DMSO n=26, HOM SIRO n=20; ash1l + sunitinib WT DMSO n=14, HOM DMSO n=13, HOM SUN n=12; ash1l + ezetimibe WT DMSO n=15, HOM DMSO n=12, HOM EZE n=9; ash1l + rosuvastatin WT DMSO n=27, HOM DMSO n=22, HOM ROSU n=22; ash1l + repaglinide WT DMSO n=11,

HOM DMSO n=16; HOM REPAG n=14.

Data exclusions	Cells were excluded based on poor cell quality based on the overall percent of mitochondrial, hemoglobin, and ribosomal genes expressed in each cell and lack of sufficient read depth. After demultiplexing for gRNA dentity, cells with more than one (singlet)n gRNA identity assigned were excluded from downstream analysis. False positive (cells with gRNA identity but no impact on the transcriptome) identified based on gRNA an transciptomic clustering using weighted nearest neighbor analysis , cells that did not cluster with other cells of the same gRNA identity were removed and clusters were evaluated from global KO of the targeted gene.<br><br>For zebrafish larvae, heterozygous mutants were not included in the analysis.
Replication	For each CRISPR-KO screen in each cell-type, 12 technical replicates were used and pooled together. Then submitted as 20,000 cells per lane across 4 lanes which represented the technical replicates in the downstream analyses.<br><br>For heterozygote zebrafish in-crosses, behavioral assays were repeated 2-4 times.
Randomization	For both experiments, technical replicate wells of iNPCs, iGLUTs, and iGABAs were pooled into a single library, randomly separated into 4 batches sequenced across 4 lanes (10x Genomics scRNA sequencing).<br><br>For zebrafish heterozygous in-crosses, larvae were exposed to experimental conditions and genotyped after the conclusion of the experiment.
Blinding	Following stem cell reprogramming, the clinical origin of each sample was blinded to those performing neuronal differentiations, RNA purification, and RNA sequencing in the validation of the cell lines. For the experiment, gRNA identity and proportion is inherently unknown - technical replicates were pooled and randomly divided across 4 lanes.<br>Experimenters were blind to genotype for behavioral heterozygous in-crosses until after the experiment concluded.

Reporting for specific materials, systems and methods

We require information from authors about some types of materials, experimental systems and methods used in many studies. Here, indicate whether each material, system or method listed is relevant to your study. If you are not sure if a list item applies to your research, read the appropriate section before selecting a response.

Materials & experimental systems

n/a	Involved in the study
☐	☒ Antibodies
☐	☒ Eukaryotic cell lines
☒	☐ Palaeontology and archaeology
☐	☒ Animals and other organisms
☒	☐ Clinical data
☒	☐ Dual use research of concern
☒	☐ Plants

Methods

n/a	Involved in the study
☒	☐ ChIP-seq
☒	☐ Flow cytometry
☒	☐ MRI-based neuroimaging

Antibodies

Antibodies used	Antibody Species Vendor Catalog # Dilution<br>anti-MAP2 chicken Invitrogen, Abcam PA1-10005, ab5392 1:1000<br>anti-Nestin rabbit Millipore ABD69 1:200<br>anti-vGLUT1 rabbit Synaptic systems 135-303 1:200<br>anti-GABA rabbit Sigma-Aldrich A2052 1:200<br>TOMM20 mouse Santa Cruz Biotechnology sc-17764 1:200<br>Total OXPHOS n/a Abcam AB-317270 1:500<br>anti-mouse donkey Jackson ImmunoResearch 715-605-151 1:500<br>anti-rabbit donkey Jackson ImmunoResearch 711-545-152 1:500<br>anti-chicken donkey Jackson ImmunoResearch 715-605-150, 703-545-155 1:500
Validation	All antibodies were previously validated (in Brennand et al 2011, Brennand et al 2015, and Ho et al 2017) for immunocytochemistry in human cells.<br>Validation and references for MAP2 antibody provided at https://www.abcam.com/map2-antibody-ab5392.html<br>Validation and references for Synapsin1 antibody provided at https://sysy.com/product/106011

Eukaryotic cell lines

Policy information about cell lines and Sex and Gender in Research

Cell line source(s)	Cultured fibroblast-reprogrammed hiPSCs<br>Validated control hiPSCs for CRISPRa/RNAi were selected from a previously reported case/control hiPSC cohort of childhood onset SZ (COS) (Hoffman et al, Nat Comm 2017). The following controls were used for CRISPRa/RNAi (hiPSC NPCs NSB553(XY), NSB3182(XX).

	Commercial cell lines:<br>HEK293T cells for virus generation: Verma Lab (https://jvi.asm.org/content/73/1/576)
Authentication	Samples were confirmed to key karyotypically normal using the Illumina Core Exome Genotyping Chip (Illumina, 20030770) and cnvPartition 3.2.0 (Illumina, Genome Studio). No cell lines displayed karyotypic abnormalities (no reported CNVs ?2.5 MB in size). All reported CNVs are shown in each certificate of analysis. hiPSC lines were confirmed to be viable post-thaw, achieving a minimum of 50% confluency within 10 days). Sample identity testing was performed using the SNPTrace assay, confirming correct sample association between parental fibroblast and hiPSC line. Gene expression analysis was using a custom Nanostring panel27 to confirm expression of pluripotency markers such as POU5F1, NANOG, and SOX2, and lack of expression of early differentiation markers such as AFP (Mesoderm), SOX17 (Endoderm), and NR2F2 (Ectoderm). A scorecard panel was used to confirm propensity to differentiate27. All hiPSC lines used in this study passed the above QC and have a certificate of analysis.
Mycoplasma contamination	As part of the hiPSC validation process, all samples were tested for the absence of Mycoplasma (Lonza, LT07-710) and confirmed to be sterile (Hardy Diagnostics, K82). NPCs were tested for mycoplasma monthly with all test being negative.
Commonly misidentified lines<br>(See ICLAC register)	N/A

Animals and other research organisms

Policy information about studies involving animals; ARRIVE guidelines recommended for reporting animal research, and Sex and Gender in Research

Laboratory animals	Zebrafish larvae were raised at 28°C on a 14:10 hour light:dark cycle. Larvae were grown in 150 mm Petri dishes in blue water (0.3g/L Instant Ocean, 1 mg/L methylene blue, pH 7.0) at a density of 60-80 larvae per dish. Behavioral assays were conducted in zebrafish larvae at 5-7 dpf.
Wild animals	NA
Reporting on sex	At these developmental stages, sex is not yet determined.
Field-collected samples	NA
Ethics oversight	All procedures involving zebrafish were conducted in accordance with Institutional Animal Care and Use Committee (IACUC; Protocol #2024-20054) regulatory standards at Yale University.

Note that full information on the approval of the study protocol must also be provided in the manuscript.

Plants

Seed stocks	NA
Novel plant genotypes	Na
Authentication	NA
